# Priority Setting and Influential Factors on Acceptance of Pharmaceutical Recommendations in Collaborative Medication Reviews in an Ambulatory Care Setting – Analysis of a Cluster Randomized Controlled Trial (WestGem-Study)

**DOI:** 10.1371/journal.pone.0156304

**Published:** 2016-06-02

**Authors:** Olaf Rose, Hugo Mennemann, Carina John, Marcus Lautenschläger, Damaris Mertens-Keller, Katharina Richling, Isabel Waltering, Stefanie Hamacher, Moritz Felsch, Lena Herich, Kathrin Czarnecki, Corinna Schaffert, Ulrich Jaehde, Juliane Köberlein-Neu

**Affiliations:** 1 Clinical Pharmacy, Institute of Pharmacy, University of Bonn, Bonn, Germany; 2 Department of Health Care Management and Public Health, Schumpeter School of Business and Economics, University of Wuppertal, Wuppertal, Germany; 3 Department of Social Sciences, University of Applied Science Muenster, Muenster, Germany; 4 Department of Pharmaceutical and Medicinal Chemistry, Clinical Pharmacy, Westfaelische Wilhelms-University, Muenster, Germany; 5 Institute of Medical Statistics, Informatics and Epidemiology (IMSIE), University of Cologne, Koeln, Germany; 6 Elefanten-Apotheke, Steinfurt, Germany; University of Padova, ITALY

## Abstract

**Background:**

Medication reviews are recognized services to increase quality of therapy and reduce medication risks. The selection of eligible patients with potential to receive a major benefit is based on assumptions rather than on factual data. Acceptance of interprofessional collaboration is crucial to increase the quality of medication therapy.

**Objective:**

The research question was to identify and prioritize eligible patients for a medication review and to provide evidence-based criteria for patient selection. Acceptance of the prescribing general practitioner to implement pharmaceutical recommendations was measured and factors influencing physicians’ acceptance were explored to obtain an impression on the extent of collaboration in medication review in an ambulatory care setting.

**Methods:**

Based on data of a cluster-randomized controlled study (WestGem-study), the correlation between patient parameters and the individual performance in a medication review was calculated in a multiple logistic regression model. Physician’s acceptance of the suggested intervention was assessed using feedback forms. Influential factors were analyzed.

**Results:**

The number of drugs in use (p = 0.001), discrepancies between prescribed and used medicines (p = 0.014), the baseline Medication Appropriateness Index score (p<0.001) and the duration of the intervention (p = 0.006) could be identified as influential factors for a major benefit from a medication review, whereas morbidity (p>0.05) and a low kidney function (p>0.05) do not predetermine the outcome. Longitudinal patient care with repeated reviews showed higher interprofessional acceptance and superior patient benefit. A total of 54.9% of the recommendations in a medication review on drug therapy were accepted for implementation.

**Conclusions:**

The number of drugs in use and medication reconciliation could be a first rational step in patient selection for a medication review. Most elderly, multimorbid patients with polymedication experience a similar chance of receiving a benefit from a medication review. Longitudinal patient care should be preferred over confined medication reviews. The acceptance of medication reviews by physicians supports further implementation into health care systems.

**Trial Registration:**

ISRCTN ISRCTN41595373

## Introduction

Medication reviews are recognized by the World Health Organization and the International Pharmaceutical Federation as a patient-oriented pharmaceutical care service and are offered in many countries worldwide to improve the quality of drug therapy [1,2]. The Pharmaceutical Care Network Europe (PCNE) defines a medication review as “a structured evaluation of patients’ medicines with the aim of optimizing medicine use and improving health outcomes. This entails detecting drug-related problems and recommending interventions” [3]. Various studies and reviews have shown the benefit of medication reviews in enhancing the quality of therapy and in reducing costs [4–9]. Through these medication reviews, a reduction in the number of drug-related problems (DRPs) was demonstrated in specific diseases and settings [10–12]. Improvements in the reduction of LDL-cholesterol, HbA_1c_ and blood pressure could be attained, as well as enhanced medication adherence and quality of life [13–20]. To date, the selection of patients for a medication review is primarily conducted on assumptions, through certain diagnosis, polymedication or predetermined by third-party payers, such as health insurers [21–24]. A study by Isaksen et al. tried to identify risk factors for drug related problems for prioritization of pharmaceutical services [25]. Medication reviews became the most prominent pharmaceutical patient oriented service in many countries [2]. Several national health care systems are considering their implementation or have already done so [2,26]. Patient selection for a medication review is done mainly by the pharmacist (“pull referral”) or by the health insurances (“push referral”) [24]. In Switzerland and Australia, a medication review is typically initiated by the pharmacist, whenever drug related problems are detected [22,27]. The Australian Residential Medication Management Review (RMMR) on the other hand needs to be initiated by a physician for reimbursement [28,29]. In the United States (US) patients are referred to a medication review mainly through insurance companies [23]. Medication review programs in the US vary and health expenditure might be an unpretentious criterion for patient selection [23]. In Great Britain patients are eligible for a Medicines Use Review (MUR) if they have been prescribed two or more medicines and are regular users of the pharmacy [30]. Given the limited resources in health care, an important consideration is whether there are certain patients who might benefit more from a medication review than others, and to whom such a service should preferentially be offered. Interventions recommended by pharmacists can only reach the patient if they are approved by the decision maker, the physician. A consensus between all health care providers is likely to support therapy [31]. A study by Doucette et al. examining physicians’ acceptance of recommendations made by pharmacists through a medication review found a prescriber response rate of 47.4% over all suggestions in a community setting in the US [32]. Chau et al. obtained an implementation of 46.2% of interprofessional recommendations in a recent study also undertaken in a community setting, this time in the Netherlands [33]. In nursing-home or hospital settings a higher implementation rate of 75.6% and 90.0% respectively could be reached [34,35].

### Objective

The main research question was to provide data to identify eligible patients for a medication review. Therefore, the correlation between patient parameters and the individual performance in a medication review was analyzed. Since acceptance rates may differ with the setting of the national health care system as well as with the primary diagnosis, it was also aimed to generate data on the acceptance of interprofessional collaboration and to assess the acceptance of the pharmaceutical recommendations by the general practitioners (GPs). Furthermore, a closer look at potential influential factors and an in-depth analysis of the accepted interventions might help to recognize and overcome professional barriers. The results of this study could support health care professionals and health insurers to identify patients who are likely to carry a major benefit from a medication review and tailor such a service to achieve optimal outcomes. Findings could exemplify to health care stakeholders how to create an interprofessional, patient-oriented team with minimized medication risks and an improved efficacy of therapy by optimizing medication reviews through prespecified criteria and collaboration.

## Methods

### Design

This analysis was performed as part of a multicenter cluster randomized controlled trial (WestGem study), which was conducted from 2012 to 2015 under outpatient care conditions in Northwest Germany in order to evaluate the efficacy of a comprehensive medication review. The quality of therapy was assessed using the Medication Appropriateness Index (MAI). The MAI or MAI score is a tool to measure the quality of drug therapy. It was developed by Hanlon et al. and modified to a summated and weighted score by Samsa et al. [36,37]. The original MAI score consists of 10 questions on drug therapy as shown in Table 1 [36].

**Table 1:** The 10 Items of the Medication Appropriateness Index [36] and its weighting [37].

	Item	Weighting
**1**	Is there an indication for the drug?	3
**2**	Is the medication effective for the condition?	3
**3**	Is the dosage correct?	2
**4**	Are the directions correct?	2
**5)**	Are the directions practical?	1
**6**	Are there clinically significant drug-drug interactions?	2
**7**	Are there clinically significant drug-disease/condition interactions?	2
**8**	Is there unnecessary duplication with other drug(s)?	1
**9**	Is the duration of therapy acceptable?	1
**10**	Is this drug the least expensive alternative compared to others of equal utility?	1

**Fig 1:**
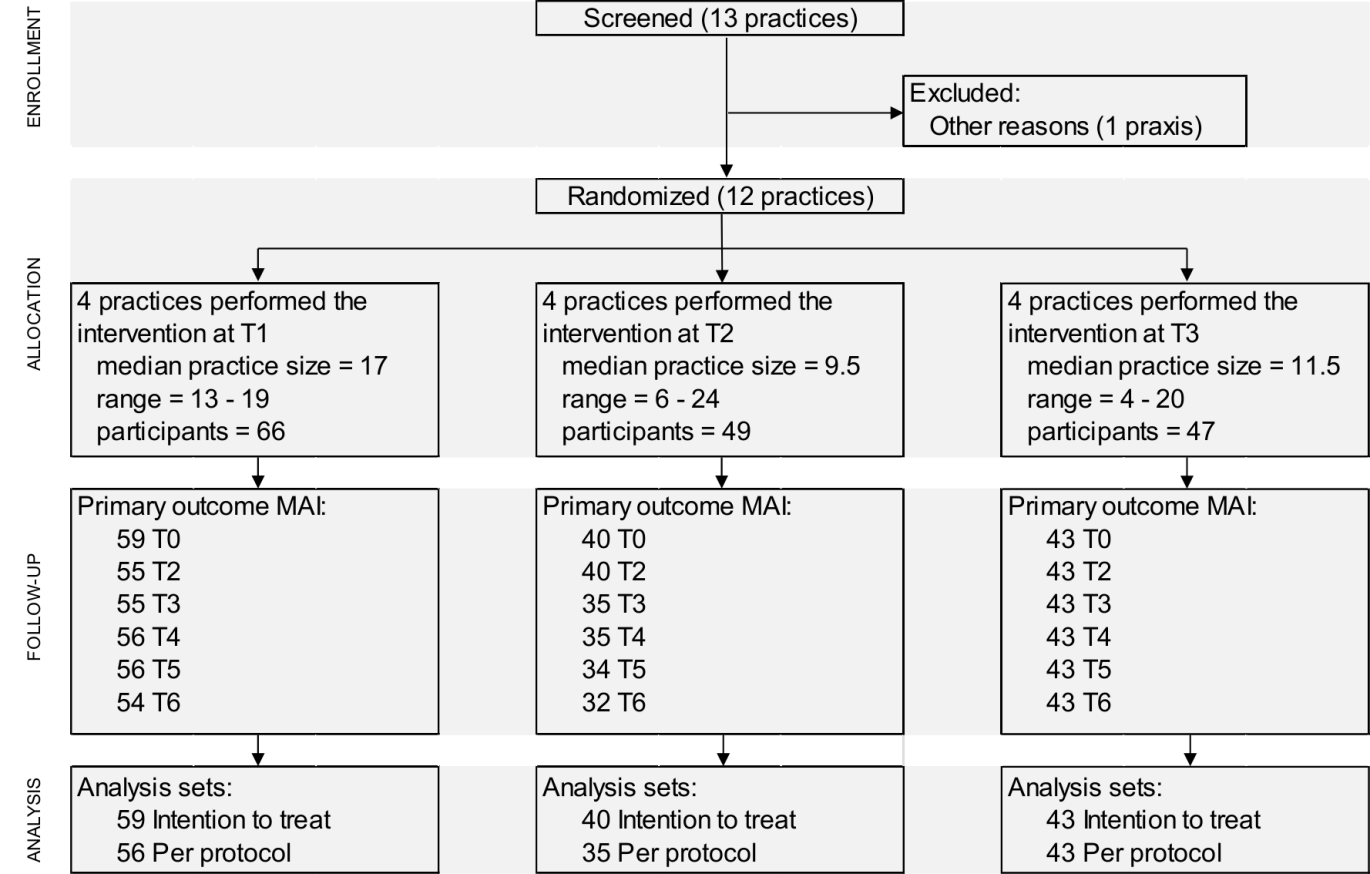
CONSORT flow diagram of the WestGem-study.

All questions need to be answered for each drug. Depending on the item, a weighted score of 1, 2 or 3 is rated if a drug related problem is detected and the scores of all items are summated [37]. Table 1 assigns the weighting scores to the 10 items. The study design of the WestGem-study was developed in line with the CONSORT statement extension for a cluster randomized controlled trials (RCT). The CONSORT flow diagram is shown in Fig 1.

The study protocol and all study forms were approved by the ethics committee of the Medical Association of Westphalia-Lippe (Aerztekammer Westfalen-Lippe), approval number AKZ-2013-292-f-s and conducted to the principles of the World Medical Association (WMA) Declaration of Helsinki. Written informed consent was obtained from all individual participants included in the study. The written statement was obtained from the patient by the GP. One copy was archived by the general practitioner; one copy was handed to the patient. Clinical research associates proved obtainment of the written informed consent statement during clinical on-site monitoring. The ethics committee of the Medical Association of Westphalia-Lippe has approved this procedure. The study is registered at the ISRCTN registry DOI 10.1186/ISRCTN41595373. The significance level was set to 0.05 (5%) for all analyses. A more detailed description of the methodology was published in the WestGem study protocol [38].

### Participants and setting

The study was conducted in ambulatory care. Inclusion criteria for the patients were age 65 years or older, ≥ 3 chronic diseases out of 2 different organ systems with at least one cardiovascular disease, use of 5 or more systemically available drugs, given formal consent of participation in the study and a history of ≥ 1 visit to the GP during each of the past 3 quarters of the year (January-March, April-June, July-September and October-December). Patients with an insufficient ability to speak or read German, participation in other studies or with severe, end-stage diseases (e.g. end-stage cancer or end-stage kidney disease) that would likely lead to death within 12 months, as based on the physician’s estimation, were excluded from the study. The number of patients was calculated according to Woertman et al. [39]. Based on a power of 1-β = 80%, a two-sided significance level α = 5%, an effect size of Cohens d = 0.25 and regarding further parameters (ICC = 0.05), recruitment of 240 patients was planned [38]. Practices were allocated to medication reviews using a stepped wedged design. A cluster randomization was performed on the level of the primary care units. Accordingly, all GPs were initially assigned to the control group. After a 6-month observation period, GPs randomly entered one of three groups, representing the three clusters. Physicians of cluster one switched to the intervention phase in January 2014 with their patients, the remaining physicians of cluster two and three stayed in the control phase. Three months later the physicians of cluster two left the control phase and entered the intervention phase. Cluster three followed with a lag of another three months. The intervention phase with the first medication reviews was implemented between January and September 2014. A biometrician who was not involved in the field work, randomly selected the clinics. To avoid changes in physician’s prescription behavior, random lists remained concealed until each allocation date. Patients recruited by the GPs received standard care during the control period. At intervention time, the first comprehensive medication review was performed by the blinded pharmacists based on the medical records and the results of a standardized, comprehensive patient interview. The results of the medication review were provided to the GP in a SOAP-based protocol. The patient interview and the medication review were repeated after 6 months between June 2014 and March 2015. The GP was free to accept or refuse any suggestions and kept unrestricted individual freedom of choice at all times during the study period.

### Interventions

The intervention consisted of a collaborative, comprehensive medication management and involved the three professions: physicians, home care specialists and pharmacists. The participating 12 physicians were individual GPs in their own clinic and had no former experience with comprehensive medication reviews. The three home care specialists worked for the county or for an organization that was responsible for the county’s obligation to provide independent health care consulting. Six study pharmacists, all specialized in clinical pharmacy and pharmacotherapy, appeared as researchers independent from a pharmacy. Study patients were recruited in the GPs’ practices. The GPs provided all medical data to the home care specialists who visited the patients at home, performed their assessments and acquired the required data for the pharmacists’ medication review according to a standardized questionnaire. All medical and drug-related data was anonymized and provided to the blinded pharmacists who conducted a repeated comprehensive medication review (Pharmaceutical Care Network Europe medication review “type 3”, or “advanced medication review”), which was supplied to the GPs for decision-making and potential therapeutic changes [40]. According to the PCNE definition, an advanced medication review is based on medication history, patient information and clinical information [40]. It was performed by a minimum of two study pharmacists and consisted of medication reconciliation, a drug-drug interaction check, an assessment on pharmacotherapy and current medical treatment guidelines, considerations of potential adverse drug reactions, medical goals, patient goals and drug safety aspects (as correct drug administration, handling, timing and dosing). Treatment costs were considered. The patient’s medical history and current laboratory data were assessed by the pharmacists. Potentially inappropriate medication (PIM) in the elderly was identified using the PRISCUS list [41]. Recommendations on therapeutic monitoring and a new medication plan were provided. Suggested interventions were numbered and mentioned again at the end of the SOAP-based review. The medication review was repeated after six months.

### Outcome: priority setting

#### Endpoint

To search for patient characteristics that might allow for priority setting in medication reviews, the potential to have a major benefit from a medication review must be defined as preexisting criteria with a definite cutoff. The MAI score was calculated for all study patients. There are indeed several studies utilizing the MAI score as an endpoint; however, to our knowledge, no randomized controlled study has defined a major or modest benefit from a medication review. MAI scores vary widely with diagnosis and setting. The level of the baseline MAI score determines a potential reduction through a medication review [42,43]. To avoid a mere arbitrary MAI score cutoff number in defining a major benefit, we chose a Cochrane Review by Patterson et al. as a benchmark, which identified 5 studies of medication reviews as being of better quality [4]. The analysis found a mean reduction in the MAI score of 3.88 points for these studies. As the included studies carry a high relevance, we defined patients of our study with a reduction of ≥3.88 points in the MAI score as having a major benefit from the intervention.

#### Influential factors

To analyze whether certain patient groups in our study population had a major benefit from the medication review and hence might be prioritized, we tested several patient parameters and searched for suitable indicators. The influence of the parameters on receiving a greater benefit status was analyzed in a univariate logistic regression followed by a multiple logistic regression model with backward selection (LR method). Possible cutoff values for quantitative parameters were computed with receiver operating characteristic (ROC). The glomerular filtration rate of all patients at baseline was calculated according to the Cockcroft-Gault equation. For patients with a BMI of ≥ 30 kg/m², body weight was corrected and the lean body mass was used [44]. The study-population for this assessment consisted of all patients, for whom a MAI score at study entry (T0) and at the end of the study (T6) was available and hence differed from the original ITT population (comprising of patients with a MAI score at study entry and at least 2 MAI scores after the intervention was performed). The odds ratio was calculated. In a first approach, explicit baseline characteristics were analyzed that can be obtained early in the medication review process at the time of data collection and the initial patient interview. These parameters were gender, age, estimated glomerular filtration rate (eGFR), number of drugs in use at baseline, number of differences between the prescribed and used drugs, Cumulative Illness Rating Scale (CIRS-G) severity index, number of diagnoses, number of responsible health care providers (specialists and hospitals) and the number of visits to the general practitioner [45,46]. Results here could lead to a fast selection of eligible patients by the pharmacist or health care professional.

In a second approach, the implicit parameters baseline MAI score and the length of the medication and therapy services (length of the intervention) were tested along with gender, age, estimated glomerular filtration rate and the number of drugs at baseline as prediction factors. Data on the MAI score and the longitudinal service was generated later in the pharmaceutical work up during a medication review.

#### Outcome: acceptance analysis

The acceptance of the pharmaceutical recommendations in the medication review was analyzed based on the GPs appraisal on the feedback form, which included a table enabling the GP to respond to every single recommendation made by the pharmacists. GPs could rate their acceptance in 3 categories of approval: partial/complete, no action/refusal or further information requested. We excluded forms without any feedback and requests for further information and accepted that this might reduce the number of considered patients in this assessment. The feedback was subsequently allocated to one of the three domains of stopping an existing drug, starting a new drug or changing an existing drug’s dose. To identify influential factors of the prescriber’s acceptance of the recommendations, ordinary least squares (OLS) regression with the approval rate as the dependent variable was conducted. In a first approach, we performed univariate analyses and then considered all influential factors within one model. We clustered the standard error at practice level to adjust for correlations within physicians. To find out whether certain influential factors might lead to a higher or lower frequency in the physician’s acceptance of a suggested intervention, influential factors of patient baselines, morbidity, drug therapy and patient-prescriber relation were considered.

In detail, the 3 categories of starting a drug, stopping a drug or changing the dose of a drug were correlated versus the patient’s age, gender, education level, body mass index (BMI), Morbidity (CIRS-G), number of prescribed drugs, number of drug-related events, number of patient-reported adverse events, number of potentially inadequate medications (PIM), number of patient visits to the GP per quarter (3 months), patient reported health (visual analog scale, VAS), social support (FSozu14), cognitive impairment (MMSE), mobility (Tinetti test), daily functioning (activities of daily living, ADL and iADL), and adherence (Morisky score) in an multivariate ordinary least squares (OLS) regression.

Statistical analysis in this study was performed using Stata^®^ statistical software, version 13.1 (StataCorp, College Station, TX) and SPSS Statistics 22 (IBM Corp., Armonk, NY, USA).

## Results

### Priority setting

129 patients of the intention to treat (ITT) population of 142 patients met the criterion with a baseline MAI score at T0 and a MAI score at T6, and were included in the analysis. Patient baseline characteristics are shown in Table 2. 73 patients out of this group had a reduction in MAI score of 3.88 or more and were considered as patients with a major benefit from the medication review.

**Table 2:** Baseline characteristics of the studied patient group (N = 129). Data is presented as mean ± SD unless otherwise indicated.

Parameter		Total	Major benefit	Minor benefit	p-value
**Assessed ITT-collective [N (%)]**		129 (100%)	73 (100%)	56 (100%)	.
**Female Gender [N (%)]**		69 (53.5%)	39 (53.4%)	30 (53.6%)	1.000
**Length of the intervention[N (%)]**	12 months9 months6 months	54 (41.9%)32 (24.8%)43 (33.3%)	37 (50.7%)19 (26%)17 (23.3%)	17 (30.4%)13 (23.2%)26 (46.4%)	0.017
**Age**		76.4 ± 6.3	76.7 ± 6.2	76.1 ± 6.4	0.694
**eGFR**		55.6 ± 21.5	52.6 ± 21.3	59.6 ± 21.3	0.071
**Mean summated baselineMAI score per patient**		31.3 ± 24.8	40.0 ± 26.8	19.9 ± 16.0	<0.001
**Number of drugs**		9.4 ± 3.2	10.5 ± 3.4	8.1 ± 2.3	<0.001
**Number of differences between GP-prescribed and used drugs**		4.5 ± 3.5	5.4 ± 3.7	3.3 ± 2.8	0.001
**CIRS-G severity index**		1.6 ± 0.4	1.7 ± 0.4	1.6 ± 0.4	0.090
**Number of diagnoses**		13.1 ± 5.8	13.5 ± 6.3	12.6 ± 5.1	0.526
**Number of health-care providers(specialists and hospitals)**		3.0 ± 2.1	3.2 ± 2.3	2.6 ± 1.7	0.167
**Number of GP visits (during past 6 months)**		12.3 ± 8.4	12.0 ± 8.9	12.7 ± 7.7	0.396

The multivariable regression in approach 1 on early obtainable parameters was significant for the number of drugs per patient and the number of differences between GP-prescribed drugs and drugs used by the patient (Table 3). For each additional drug in use the chance of having a major benefit from a medication review increases 1.282 times and for each discrepancy between a prescribed drug and what is actually taken at home 1.181 times.

**Table 3:** Results of the multiple logistic regressions after automatic selection, approach 1, early detectable parameters and approach 2, later detectable parameters.

Variable	Comparison	OR	95%-CI	p-value
**Approach 1, early detectable parameters**				
**Number of drugs per patient**	1 difference	1.282	(1.109 to 1.1482)	0.001
**Number of differences in drugs between GP and patient**	1 difference	1.181	(1.034 to 1.350)	0.014
**Approach 2, later detectable parameters**				
**Mean summated baseline MAI score per patient**	1 point higher score	1.061	(1.031 to 1.093)	< 0.001
**Length of the intervention**	9 months vs. 12 months 6 months vs. 12 months	0.2480.211	(0.078 to 0.791) (0.077 to 0.578)	0.006 (overall)0.0180.002
**Number of differences in drugs between GP and patient**	1 difference	1.206	(1.048 to 1.387)	0.009

Multivariable regression in approach 2 of the parameters that are generated later in a medication review was significant for the baseline MAI score (p<0.001), the time of change from the control to the intervention group (overall p = 0.006) and again the discrepancy between prescribed and used drugs (p = 0.009) (Table 3). The chance of benefiting from a medication review rises by 1.061 per 1-point increase in the baseline MAI score. Per each discrepancy between the prescribed and the used drugs the chance to have a major benefit from the medication review increases 1.206 times. Patients who entered the medication review service 3 months later than the first group, and hence experienced a 3-month shorter intervention, had a fourfold-reduced chance of having a major benefit from the medication review. Patients who entered the medication review 6 months later and experienced a 6-month shorter intervention had a 4.7 times lower chance of having a major benefit.

**Fig 2:**
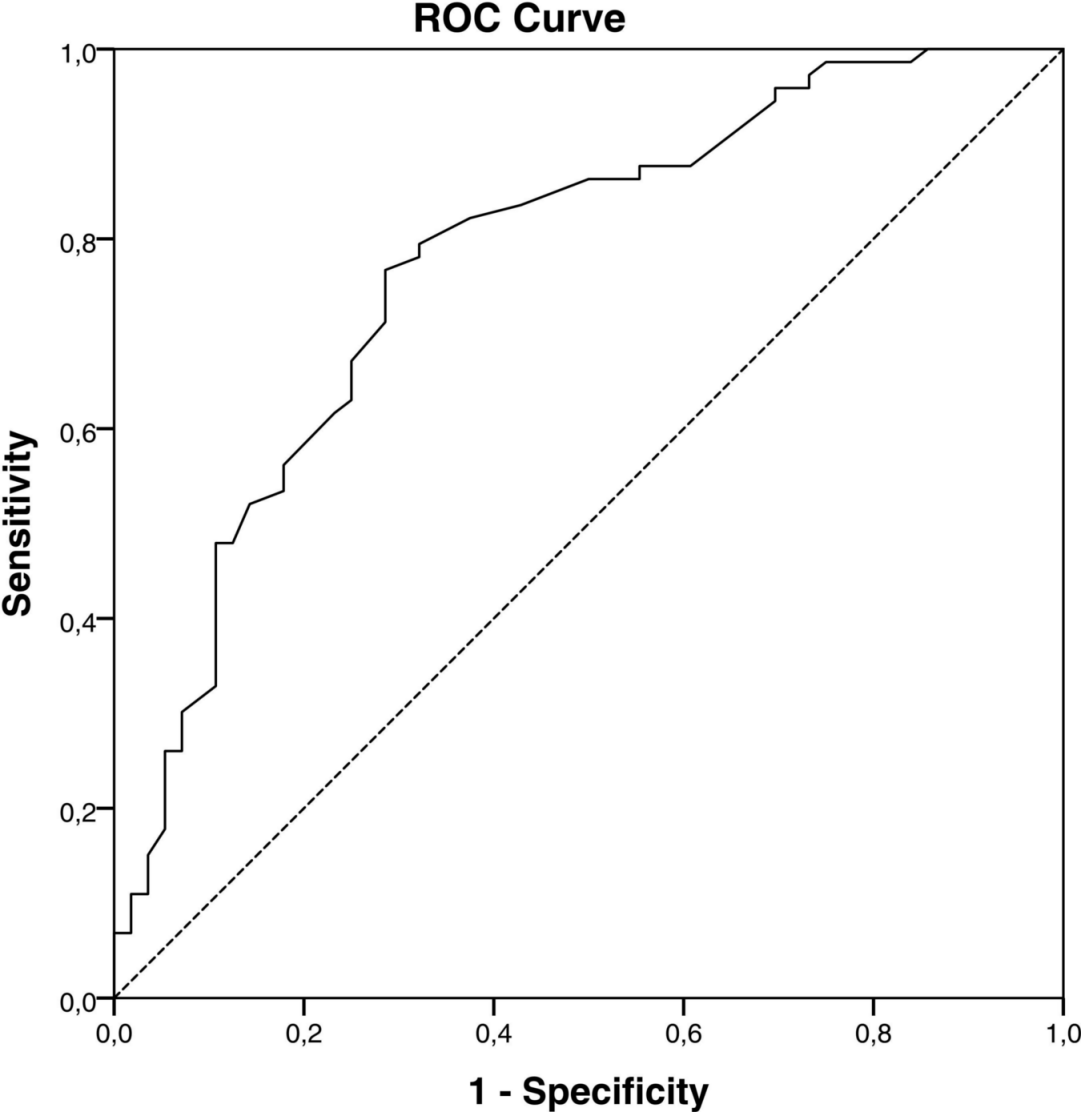
ROC curve for the baseline MAI score.

To search for potential cutoff values, a separate ROC curve was performed on the results of approach 1 and on approach 2. The ROC analysis of the data for the MAI score (Fig 2) found that a potential cutoff to initiate a medication review could be a MAI score of ≥ 24 (AUC = 0.777, 95%-CI = 0.753–0.897) for this patient collective. For this cutoff level the sensitivity is 77% and the specificity is 71%.

The ROC curves for the number of differences in drugs prescribed by the GP and for the length of the intervention did not allow for predicting a reasonable cutoff value.

### Acceptance analysis

As a result of the medication reviews, the pharmacists proposed 1705 intervention recommendations to the physicians for 142 patients. 1082 of these recommendations (63.5%) for 104 patients were rated by the physicians using the feedback form. 667 of these responses for 103 patients could be allocated to the 3 domains of stopping an existing drug, starting a new drug or changing the dose of an existing drug. The other interventions were not drug-related but included recommendations on laboratory data, monitoring or patient education. Table 4 shows the characteristics of the ITT group for the 103 eligible patients for the acceptance analysis.

**Table 4:** Patient characteristics for the ITT group and for eligible patients of the acceptance analysis. Data is presented as mean (SD) unless otherwise indicated.

Parameter	ITT population	Acceptance analysis
**Number of patients**	142	103
**Age (years)**	76.7 (6.3)	76.6 (6.4)
**Gender (female) [N (%)]**	76 (53.5)	67 (55.3)
**BMI (kg/m**^**2**^**)**	28.4 (4.3)	28.4 (4.3)
**Morbidity (CIRS-G)**	1.6 (0.4)	1.7 (0.4)
**No. of diagnoses**	12.7 (5.7)	13.7 (6.1)
**No. of prescribed drugs**	9.4 (3.1)	9.7 (3.3)
**No. of drug-related problems**	7.3 (3.4)	7.1 (3.4)

A total of 366 (54.9%) of the 667 recommendations on drug therapy were implemented by the physicians, 301 (45.1%) were refused (Table 5). Reasons for non-acceptance were the necessity for further information (18%), medical reasons (9%), budgetary reasons (5%) or special aspects in the patient’s treatment history that were unknown to the pharmacist (68%). The in-depth analysis showed that 133 (53.4%) recommendations to stop a drug were implemented and 121 (47.6%) were refused. With 129 (51.8%) implemented recommendations, a similar rate was found for prescribing a new drug, while 120 (48.2%) suggestions were refused. On changing the dose of an existing drug, 104 (63.4%) recommendations were implemented and 60 refused (36.6%) (Table 5).

**Table 5:** Acceptance analysis of pharmacists’ recommendations. Data is presented as n (%).

Suggestions for interventions in pharmacotherapy	Implemented by physicians	Refused by physicians
**To stop prescribing an existing drug**	133 (53.4)	121 (47.6)
**To start prescribing a new drug**	129 (51.8)	120 (48.2)
**To change the dose of an existing drug**	104 (63.4)	60 (36.6)
**Total**	366 (54.9)	301 (45.1)

The chance that a GP accepts recommendations to change a drug’s dose was significantly more likely than to start a new drug (p = 0.041). The difference in accepting recommendations on changing a drug`s dose and stopping a drug in contrast was not significant (p = 0.085). The test of patient baseline characteristics that might influence the GPs acceptance of pharmaceutical suggestions showed that in patients with a lower education level, cognitive impairment and good mobility, the GPs were more likely to stop a drug. In female patients, the GPs were more likely to accept a recommendation to start a new drug and in patients with less GP visits they were less likely to stop a drug. GPs implemented more recommendations on changing a dose if the patient had a high BMI, manifold drug-related problems, good social support, good daily functioning or cognitive impairment. They implemented fewer recommendations on changing a dose as patient age increased and in patients with a good self-reported health status (all parameters p< 0.05).

### Strengths and limitations

To our knowledge, this is the first study aiming to search for patient baseline parameters that help to identify patients who receive a major benefit from a medication review. The patient population of the WestGem study included multimorbid patients with a focus on cardiovascular diseases aged 65 or older with 5 or more drugs in use (polymedication). The inclusion criteria might already narrow down the eligible patients for a medication review and all results must be seen in this context. The cutoff level of a reduction in the MAI score of 3.88 for a major benefit from a medication review cannot be seen as a definite number and might vary with the setting. The effects of a medication review are dependent on the acceptance of the pharmaceutical suggestions by GPs. The results of the acceptance of the collaborative medication review by the physicians derive from quantitative analyses only; a qualitative approach was not analyzed here. Some of the participating 12 physicians responded poorly on the feedback forms of the suggested interventions. The study was conducted as a regional project in two model regions in North Rhine-Westphalia, Germany. The acceptance and effects of a collaborative medication review need to be studied also in different jurisdictions and settings.

## Discussion

Among the parameters that are initially available from the medical record or the laboratory data or that are obtainable by a patient interview, the number of drugs in use and a high discrepancy between drugs prescribed compared to the drugs actually taken at home could be identified as determining factors in having a special benefit from a medication review. Therefore, the number of drugs in use could be a valid and easily accessible criterion in selecting patients for a medication review. In addition, medication reconciliation could be a reasonable first step in a medication review and a high discrepancy could be another decision criterion to initiate longitudinal patient care. To benefit from a medication review patient age, gender, eGFR, CIRS-G severity index, number of diagnoses, number of health care providers and the number of visits to the GP during the last 6 months were not identified as covariates. These parameters should not be considered as useful patient selection criteria for a medication review. The results are quite surprising as morbidity (CIRS-G severity index) and kidney function (eGFR) were regarded as potential risk factors for drug-related problems in a recent qualitative research and thus could be expected to have a correlation to the outcome of a medication review [47]. The absence of other defined patient baseline parameters shows that all elderly patients with cardiovascular disease using 5 or more drugs are eligible for a medication review. Patients in our analysis experienced a major benefit from the medication review if the quality of therapy was very low at baseline (as indicated by a high baseline MAI score) or if they received longitudinal care with repeated medication reviews. Unfortunately, the calculation of the MAI score is very time consuming, and might be regarded as a medication review in itself and hence is not useful for identifying eligible patients in routine care [48]. Otherwise, a MAI score of ≥24 could be suggested for the selection of eligible elderly patients with cardiovascular disease and similar inclusion criteria for a medication review. The effect of the medication review on the quality of therapy increased with the duration that the patient care was performed. Physicians tended to implement pharmaceutical implementations over time. Future medication reviews should emphasize the aspects of longitudinal patient care with repeated rather than on confined pharmaceutical activities. These findings are in contrast to a study by Chinthammit et al. which favors shorter and less comprehensive reviews regarding the cost-effectiveness of a medication review [49].

With 54.9% of the suggested interventions on pharmacotherapy being implemented, both, physicians and pharmacists showed a relatively high level of appreciation for the interprofessional and collaborative medication review in an ambulatory setting. As acceptance increases over time, a longitudinal patient service could be more effective with growing confidence between the professions. Reasons not to implement the therapeutic suggestions were mainly due to the patient’s personal treatment history, which was unknown to the pharmacist. Further communication thus could increase the acceptance. The creditable results in interprofessional collaboration implicates that professional barriers can be overcome.

## Conclusions

Besides the number of drugs in use and the discrepancies between prescribed and actually taken medications, the results of our study indicate that no early obtainable parameters based on medical records or patient interviews can be identified during the initial steps of a medication review in order to predict whether the patient might particularly benefit from the service. Using the number of drugs in use as a criterion in patient selection for a medication review has proven to be significant and is accessible for all health care professionals. Medication reconciliation could be another first step in identifying patients with special circumstances. Otherwise, all elderly multimorbid patients with polymedication and a cardiovascular disease experience a similar chance of receiving a profound benefit from a medication review, whereas morbidity and kidney function are not helpful in predetermining the outcome.

Compared to a single medication review, longitudinal patient care with repeated reviews shows both higher interprofessional acceptance and a superior patient benefit and thus should be preferred by patients and health care stakeholders. The high acceptance of medication reviews by physicians supports the implementation of a collaborative approach into health care systems.

## Supporting Information

S1 CONSORT Statement (DOCX)

S1 Data Data set on the MAI score. (DOCX)

S2 Data Data set on the GPs’ acceptance of the intervention. (DOCX)

S1 Protocol Study protocol publication. (PDF)

S2 Protocol Study protocol German, approved by ethics committee. (PDF)
