# Supplementary material for: Priority Setting and Influential Factors on Acceptance of Pharmaceutical Recommendations in Collaborative Medication Reviews in an Ambulatory Care Setting – Analysis of a Cluster Randomized Controlled Trial (WestGem-Study)
Source: PLoS One. 2016 Jun 2;11(6):e0156304. doi: 10.1371/journal.pone.0156304 (PMC4890849; doi:10.1371/journal.pone.0156304)
Supplement: S1 Data — (DOCX) [file pone.0156304.s002.docx]

Supplement 5: data set on the MAI score

| Study-ID | Gender | Nr. of  drugs | Age | Nr. of  diagnosis | Intervention time | ITT | MAI0 | MAI2 | MAI3 | MAI4 | MAI5 | MAI6 | Priority  analysis set | Benefit |
| --- | --- | --- | --- | --- | --- | --- | --- | --- | --- | --- | --- | --- | --- | --- |
|  | male | 6 | 72 | 13 | intervention after t3 | 1 | 0 | 0 | 0 | 0 | 8 | 8 | yes | minor benefit |
|  | male | 12 | 74 | 10 | intervention after t3 | 1 | 68 | 68 | 68 | 35 | 20 | 20 | yes | major benefit |
|  | female | 11 | 80 | 6 | intervention after t3 | 1 | 40 | 40 | 55 | 71 | 35 | 29 | yes | major benefit |
|  | female | 10 | 83 | 7 | intervention after t3 | 1 | 27 | 27 | 27 | 29 | 4 | 4 | yes | major benefit |
|  | female | 8 | 72 | 15 | intervention after t3 | 1 | 18 | 23 | 23 | 23 | 20 | 20 | yes | minor benefit |
|  | female | 5 | 69 | 7 | intervention after t3 | 1 | 6 | 6 | 6 | 6 | 4 | 4 | yes | minor benefit |
|  | male | 9 | 73 | 11 | intervention after t3 | 1 | 45 | 45 | 45 | 51 | 10 | 10 | yes | major benefit |
|  | male | 7 | 83 | 7 | intervention after t3 | 1 | 19 | 19 | 19 | 19 | 16 | 16 | yes | minor benefit |
|  | male | 13 | 77 | 10 | intervention after t3 | 1 | 59 | 62 | 62 | 62 | 4 | 4 | yes | major benefit |
|  | female | 6 | 71 | 9 | intervention after t3 | 1 | 27 | 27 | 27 | 30 | 21 | 6 | yes | major benefit |
|  | female | 7 | 75 | 8 | intervention after t3 | 1 | 26 | 26 | 26 | 23 | 23 | 23 | yes | minor benefit |
|  | female | 17 |  | 33 | intervention after t0 | 1 | 48 | 13 | 13 | 13 | 13 | 13 | yes | major benefit |
|  | female | 4 | 83 | 10 | intervention after t0 | 1 | 20 | 20 | 20 | 20 | 18 | 18 | yes | minor benefit |
|  | female | 10 | 72 | 13 | intervention after t0 | 1 | 59 | 59 | 45 | 41 | 41 | 13 | yes | major benefit |
|  | female | 11 | 70 | 17 | intervention after t0 | 1 | 20 | 22 | 35 | 30 | 27 | 27 | yes | minor benefit |
|  | female | 14 | 83 | 15 | intervention after t0 | 1 | 32 | 32 | 32 | 29 | 29 | 29 | yes | minor benefit |
|  | female | 12 | 68 | 13 | intervention after t0 | 1 | 6 | 6 | 5 | 5 | 5 | 5 | yes | minor benefit |
|  | female | 8 | 86 | 6 | intervention after t0 | 1 | 29 | 29 | 18 | 18 | 18 | 18 | yes | major benefit |
|  | male | 7 | 67 | 17 | intervention after t0 | 1 | 2 | 2 | 4 | 4 | 4 | 0 | yes | minor benefit |
|  | male | 7 | 81 | 37 | intervention after t0 | 1 | 13 | 18 | 2 | 2 | 2 | 2 | yes | major benefit |
|  | female | 5 | 74 | 14 | intervention after t0 | 1 | 5 | 5 | 0 | 0 | 0 | 0 | yes | major benefit |
|  | male | 9 | 68 | 12 | intervention after t0 | 1 | 42 | 27 | 26 | 26 | 0 | 0 | yes | major benefit |
|  | female | 4 | 74 | 18 | intervention after t0 | 1 | 28 | 28 | 0 | 0 | 0 | 0 | yes | major benefit |
|  | male | 15 | 86 | 21 | intervention after t0 | 1 | 21 | 21 | 12 | 12 | 12 | 12 | yes | major benefit |
|  | female | 7 | 84 | 13 | intervention after t0 | 1 | 45 | 45 | 43 | 29 | 11 | 11 | yes | major benefit |
|  | male | 16 | 84 | 19 | intervention after t0 | 1 | 29 | 7 | 3 | 1 | 1 | 1 | yes | major benefit |
|  | female | 9 | 77 | 12 | intervention after t0 | 1 | 30 | 30 | 30 | 19 | 14 | 14 | yes | major benefit |
|  | male | 12 | 65 | 10 | intervention after t0 | 1 | 61 | 10 | 10 | 12 | 12 | 14 | yes | major benefit |
|  | female | 6 | 83 | 17 | intervention after t0 | 1 | 7 | 7 | 4 | 4 | 4 | 4 | yes | minor benefit |
|  | female | 11 | 74 | 15 | intervention after t2 | 1 | 28 | 28 | 17 | 17 | 1 |  | no |  |
|  | female | 7 | 71 | 5 | intervention after t2 | 1 | 54 | 54 | 30 | 30 | 30 | 30 | yes | major benefit |
|  | female | 9 | 75 | 9 | intervention after t2 | 1 | 19 | 19 | 19 | 17 | 17 | 17 | yes | minor benefit |
|  | female | 9 | 79 | 11 | intervention after t2 | 1 | 14 | 27 | 27 | 14 | 8 | 8 | yes | major benefit |
|  | male | 11 | 87 | 9 | intervention after t2 | 1 | 30 | 30 | 30 | 34 | 34 | 21 | yes | major benefit |
|  | male | 9 | 81 | 6 | intervention after t2 | 1 | 59 | 59 |  |  |  |  | no |  |
|  | male | 14 | 78 | 9 | intervention after t2 | 1 | 121 | 111 | 111 | 39 | 33 | 33 | yes | major benefit |
|  | female | 8 | 66 | 7 | intervention after t2 | 1 | 26 | 26 | 26 | 26 | 26 | 26 | yes | minor benefit |
|  | female | 9 | 89 | 9 | intervention after t2 | 1 | 56 | 56 | 56 | 56 | 56 | 36 | yes | major benefit |
|  | female | 10 | 80 | 5 | intervention after t2 | 1 | 70 | 70 | 70 | 70 | 70 | 70 | yes | minor benefit |
|  | male | 11 | 87 | 10 | intervention after t2 | 1 | 46 | 46 | 45 |  | 19 | 19 | yes | major benefit |
|  | female | 7 | 76 | 5 | intervention after t2 | 1 | 12 | 12 | 12 | 12 | 12 | 12 | yes | minor benefit |
|  | female | 9 | 86 | 8 | intervention after t2 | 1 | 26 | 26 | 26 | 26 | 18 |  | no |  |
|  | male | 15 | 69 | 5 | intervention after t0 | 1 | 29 | 29 | 21 | 21 | 19 | 19 | yes | major benefit |
|  | male | 8 | 69 | 5 | intervention after t0 | 1 | 11 |  |  |  |  |  | no |  |
|  | female | 7 | 81 | 8 | intervention after t0 | 1 | 9 | 9 | 9 | 21 | 13 | 13 | yes | minor benefit |
|  | female | 15 | 66 | 7 | intervention after t0 | 1 | 67 | 36 | 36 | 36 | 36 | 36 | yes | major benefit |
|  | male | 8 | 88 | 5 | intervention after t0 | 1 | 29 | 29 | 16 | 16 | 0 | 0 | yes | major benefit |
|  | male | 8 | 80 | 6 | intervention after t0 | 1 | 68 | 52 | 52 | 52 | 52 |  | no |  |
|  | female | 8 | 77 | 9 | intervention after t0 | 1 | 6 | 6 | 6 | 6 | 6 | 6 | yes | minor benefit |
|  | male | 10 | 67 | 7 | intervention after t0 | 1 | 20 | 20 | 20 | 20 | 16 | 16 | yes | major benefit |
|  | female | 9 |  | 5 | intervention after t0 | 1 | 13 | 4 | 4 | 4 | 4 | 4 | yes | major benefit |
|  | male | 7 | 66 | 9 | intervention after t0 | 1 | 7 | 0 | 0 | 0 | 0 | 0 | yes | major benefit |
|  | male | 10 | 81 | 14 | intervention after t0 | 1 | 46 | 24 | 23 | 23 | 23 | 23 | yes | major benefit |
|  | female | 12 | 76 | 6 | intervention after t0 | 1 | 51 |  |  |  |  |  | no |  |
|  | male | 8 | 71 | 8 | intervention after t0 | 1 | 14 | 16 | 14 | 14 | 14 | 14 | yes | minor benefit |
|  | male | 9 | 78 | 9 | intervention after t0 | 1 | 32 | 30 | 14 | 14 | 5 | 5 | yes | major benefit |
|  | female | 12 | 79 | 6 | intervention after t0 | 1 | 31 | 11 | 14 | 14 | 6 | 6 | yes | major benefit |
|  | male | 7 | 82 | 14 | intervention after t3 | 1 | 14 | 14 | 14 | 14 | 11 | 11 | yes | minor benefit |
|  | female | 8 |  | 14 | intervention after t3 | 1 | 15 | 19 | 19 | 19 | 19 | 19 | yes | minor benefit |
|  | female | 5 | 69 | 11 | intervention after t3 | 1 | 18 | 23 | 23 | 23 | 23 | 23 | yes | minor benefit |
|  | male | 7 | 72 | 15 | intervention after t3 | 1 | 13 | 13 | 13 | 13 | 13 | 13 | yes | minor benefit |
|  | male | 9 | 73 | 8 | intervention after t3 | 1 | 46 | 46 | 46 | 46 | 46 | 46 | yes | minor benefit |
|  | female | 6 | 73 | 9 | intervention after t3 | 1 | 6 | 6 | 6 | 4 | 4 | 4 | yes | minor benefit |
|  | male | 10 | 82 | 13 | intervention after t3 | 1 | 46 | 46 | 46 | 46 | 46 | 46 | yes | minor benefit |
|  | male | 10 | 74 | 13 | intervention after t3 | 1 | 65 | 65 | 65 | 65 | 62 | 62 | yes | minor benefit |
|  | female | 5 | 79 | 8 | intervention after t3 | 1 | 12 | 2 | 6 | 6 | 6 | 6 | yes | major benefit |
|  | male | 8 | 75 | 9 | intervention after t3 | 1 | 19 | 19 | 28 | 28 | 28 | 8 | yes | major benefit |
|  | female | 7 | 86 | 12 | intervention after t3 | 1 | 18 | 18 | 18 | 18 | 18 | 18 | yes | minor benefit |
|  | female | 9 | 75 | 14 | intervention after t3 | 1 | 21 | 21 | 21 | 21 | 21 | 21 | yes | minor benefit |
|  | male | 8 | 65 | 8 | intervention after t3 | 1 | 33 | 49 | 49 | 58 | 58 | 58 | yes | minor benefit |
|  | male | 10 | 81 | 7 | intervention after t3 | 1 | 0 | 0 | 0 | 0 | 0 | 0 | yes | minor benefit |
|  | male | 8 | 77 | 7 | intervention after t3 | 1 | 0 | 0 | 0 | 0 | 0 | 0 | yes | minor benefit |
|  | male | 6 | 66 | 7 | intervention after t3 | 1 | 29 | 29 | 29 | 29 | 29 | 29 | yes | minor benefit |
|  | female | 7 | 71 | 11 | intervention after t3 | 1 | 21 | 21 | 21 | 21 | 16 | 16 | yes | major benefit |
|  | female | 12 | 80 | 7 | intervention after t3 | 1 | 23 | 23 | 23 | 23 | 23 | 23 | yes | minor benefit |
|  | male | 9 | 84 | 17 | intervention after t3 | 1 | 29 | 29 | 29 | 29 | 29 | 29 | yes | minor benefit |
|  | male | 7 | 77 | 18 | intervention after t3 | 1 | 20 | 20 | 20 | 20 | 20 | 20 | yes | minor benefit |
|  | male | 11 | 70 | 26 | intervention after t3 | 1 | 35 | 35 | 41 | 25 | 12 | 12 | yes | major benefit |
|  | male | 11 | 68 | 22 | intervention after t3 | 1 | 54 | 54 | 54 | 54 | 43 | 43 | yes | major benefit |
|  | male | 13 | 75 | 13 | intervention after t3 | 1 | 36 | 34 | 23 | 23 | 23 | 23 | yes | major benefit |
|  | male | 9 | 83 | 32 | intervention after t3 | 1 | 6 | 6 | 6 | 6 | 6 | 6 | yes | minor benefit |
|  | female | 12 | 81 | 26 | intervention after t3 | 1 | 53 | 55 | 53 | 53 | 10 | 10 | yes | major benefit |
|  | male | 7 | 68 | 18 | intervention after t3 | 1 | 9 | 21 | 21 | 21 | 21 | 21 | yes | minor benefit |
|  | female | 14 | 82 | 27 | intervention after t3 | 1 | 26 | 26 | 26 | 26 | 6 | 11 | yes | major benefit |
|  | male | 9 | 70 | 15 | intervention after t3 | 1 | 26 | 26 | 26 | 26 | 8 | 6 | yes | major benefit |
|  | male | 9 | 78 | 15 | intervention after t2 | 1 | 35 | 35 | 35 | 7 | 7 | 7 | yes | major benefit |
|  | female | 6 | 72 | 20 | intervention after t2 | 1 | 49 | 49 | 49 | 67 | 34 | 34 | yes | major benefit |
|  | female | 7 | 85 | 12 | intervention after t2 | 1 | 57 | 70 |  |  |  |  | no |  |
|  | male | 13 | 76 | 10 | intervention after t2 | 1 | 104 | 122 |  |  |  |  | no |  |
|  | female | 6 | 85 | 10 | intervention after t2 | 1 | 15 | 15 | 15 | 15 |  |  | no |  |
|  | female | 7 | 72 | 16 | intervention after t2 | 1 | 55 | 55 | 55 | 25 | 25 | 25 | yes | major benefit |
|  | female | 6 | 77 | 18 | intervention after t2 | 1 | 14 | 14 | 10 | 6 | 6 | 6 | yes | major benefit |
|  | female | 5 | 80 | 12 | intervention after t2 | 1 | 0 | 1 | 0 | 0 | 0 | 0 | yes | minor benefit |
|  | male | 9 | 65 | 9 | intervention after t2 | 1 | 28 | 28 | 28 | 28 | 28 | 28 | yes | minor benefit |
|  | female | 10 | 85 | 12 | intervention after t2 | 1 | 57 | 57 |  |  |  |  | no |  |
|  | female | 15 | 73 | 15 | intervention after t2 | 1 | 147 | 147 | 147 | 55 | 25 | 17 | yes | major benefit |
|  | female | 5 | 90 | 10 | intervention after t2 | 1 | 13 | 13 | 13 | 13 | 13 | 13 | yes | minor benefit |
|  | female | 10 | 81 | 9 | intervention after t2 | 1 | 27 | 27 | 18 | 18 | 18 | 18 | yes | major benefit |
|  | male | 13 | 82 | 12 | intervention after t2 | 1 | 72 | 53 |  |  |  |  | no |  |
|  | male | 8 | 83 | 11 | intervention after t2 | 1 | 51 | 51 | 51 | 51 | 53 | 53 | yes | minor benefit |
|  | female | 9 | 77 | 15 | intervention after t2 | 1 | 31 | 30 | 30 | 30 | 30 | 30 | yes | minor benefit |
|  | male | 10 | 86 | 13 | intervention after t0 | 1 | 29 | 36 | 36 | 34 | 30 | 32 | yes | minor benefit |
|  | male | 8 | 83 | 9 | intervention after t0 | 1 | 36 | 7 | 12 | 17 | 17 | 17 | yes | major benefit |
|  | male | 18 | 77 | 16 | intervention after t0 | 1 | 63 | 24 | 20 | 20 | 19 | 14 | yes | major benefit |
|  | female | 18 | 82 | 19 | intervention after t0 | 1 | 19 | 15 | 14 | 14 | 14 | 14 | yes | major benefit |
|  | female | 8 | 74 | 6 | intervention after t0 | 1 | 24 | 24 | 25 | 27 | 10 | 10 | yes | major benefit |
|  | female | 13 | 86 | 17 | intervention after t0 | 1 | 24 | 0 | 0 | 0 | 0 | 0 | yes | major benefit |
|  | male | 6 | 68 | 8 | intervention after t0 | 1 | 22 | 0 | 0 | 0 | 0 | 0 | yes | major benefit |
|  | female | 13 | 77 | 18 | intervention after t0 | 1 | 11 | 2 |  | 2 | 2 | 2 | yes | major benefit |
|  | female | 12 | 76 | 13 | intervention after t0 | 1 | 17 | 16 | 7 | 6 | 5 | 5 | yes | major benefit |
|  | female | 16 | 88 | 14 | intervention after t0 | 1 | 24 | 15 | 11 | 11 | 11 | 11 | yes | major benefit |
|  | female | 8 | 78 | 10 | intervention after t0 | 1 | 5 | 11 | 11 | 11 | 3 | 3 | yes | minor benefit |
|  | female | 10 | 82 | 7 | intervention after t0 | 1 | 42 | 25 | 25 | 20 | 18 |  | no |  |
|  | male | 13 | 70 | 11 | intervention after t0 | 1 | 110 | 110 | 71 | 71 | 71 | 71 | yes | major benefit |
|  | male | 6 | 75 | 11 | intervention after t0 | 1 | 8 | 8 | 3 | 3 | 3 | 3 | yes | major benefit |
|  | male | 14 | 78 | 18 | intervention after t0 | 1 | 23 | 12 | 12 | 12 | 12 | 12 | yes | major benefit |
|  | male | 7 | 73 | 11 | intervention after t0 | 1 | 2 | 0 | 0 | 0 | 0 | 0 | yes | minor benefit |
|  | male | 17 | 77 | 14 | intervention after t0 | 1 | 126 | 113 | 119 | 119 | 39 | 39 | yes | major benefit |
|  | female | 13 | 67 | 15 | intervention after t0 | 1 | 21 |  | 21 | 18 | 18 | 18 | yes | minor benefit |
|  | male | 7 | 75 | 8 | intervention after t0 | 1 | 6 |  |  |  |  |  | no |  |
|  | female | 13 | 74 | 14 | intervention after t0 | 1 | 55 | 55 | 20 | 20 | 20 | 20 | yes | major benefit |
|  | male | 10 | 66 | 11 | intervention after t0 | 1 | 32 | 32 | 2 | 2 | 2 | 2 | yes | major benefit |
|  | male | 13 | 84 | 14 | intervention after t0 | 1 | 31 | 11 | 11 | 11 | 36 | 36 | yes | minor benefit |
|  | male | 13 | 84 | 20 | intervention after t0 | 1 | 59 | 71 | 59 | 59 | 62 | 62 | yes | minor benefit |
|  | male | 9 | 79 | 16 | intervention after t0 | 1 | 26 | 20 | 20 | 20 | 17 | 17 | yes | major benefit |
|  | male | 6 | 70 | 14 | intervention after t0 | 1 | 15 | 15 | 31 | 31 | 31 | 31 | yes | minor benefit |
|  | male | 10 | 71 | 11 | intervention after t0 | 1 | 19 | 19 | 19 | 21 | 19 | 19 | yes | minor benefit |
|  | female | 7 | 71 | 15 | intervention after t3 | 1 | 23 | 28 | 26 | 26 | 34 | 23 | yes | minor benefit |
|  | female | 10 | 75 | 18 | intervention after t3 | 1 | 32 | 32 | 32 | 29 | 26 | 26 | yes | major benefit |
|  | male | 5 | 79 | 21 | intervention after t3 | 1 | 4 | 4 | 4 | 4 | 4 | 4 | yes | minor benefit |
|  | male | 11 | 80 | 10 | intervention after t3 | 1 | 34 | 34 | 34 | 34 | 12 | 12 | yes | major benefit |
|  | female | 7 | 74 | 17 | intervention after t2 | 1 | 36 | 36 | 36 | 36 | 29 | 29 | yes | major benefit |
|  | female | 5 | 74 | 27 | intervention after t2 | 1 | 4 | 10 | 10 | 8 | 12 | 12 | yes | minor benefit |
|  | female | 7 | 81 | 12 | intervention after t2 | 1 | 19 | 19 | 19 | 19 | 19 | 19 | yes | minor benefit |
|  | female | 9 | 83 | 16 | intervention after t2 | 1 | 16 | 16 | 16 | 16 | 16 | 16 | yes | minor benefit |
|  | female | 18 | 80 | 14 | intervention after t2 | 1 | 68 | 68 | 68 | 58 | 58 | 58 | yes | major benefit |
|  | female | 9 | 73 | 12 | intervention after t2 | 1 | 63 | 63 | 66 | 50 | 36 | 41 | yes | major benefit |
|  | female | 7 | 89 | 16 | intervention after t2 | 1 | 24 | 24 | 24 | 24 | 12 | 12 | yes | major benefit |
|  | male | 11 | 79 | 17 | intervention after t2 | 1 | 43 | 43 | 41 | 41 | 21 | 8 | yes | major benefit |
|  | female | 8 | 71 | 19 | intervention after t2 | 1 | 71 | 71 | 71 | 71 | 71 | 50 | yes | major benefit |
|  | female | 11 | 75 | 14 | intervention after t2 | 1 | 50 | 50 | 47 | 47 | 17 | 17 | yes | major benefit |
|  | female | 6 | 72 | 15 | intervention after t2 | 1 | 21 | 22 | 22 | 18 | 18 | 18 | yes | minor benefit |
