# Supplementary material for: Priority Setting and Influential Factors on Acceptance of Pharmaceutical Recommendations in Collaborative Medication Reviews in an Ambulatory Care Setting – Analysis of a Cluster Randomized Controlled Trial (WestGem-Study)
Source: PLoS One. 2016 Jun 2;11(6):e0156304. doi: 10.1371/journal.pone.0156304 (PMC4890849; doi:10.1371/journal.pone.0156304)
Supplement: S2 Data — (DOCX) [file pone.0156304.s003.docx]

Supplement 6: individual patient data on the acceptance of a GP to accept a suggestion of a pharmacist regarding stopping or starting a drug and to change a dose of a prescribed drug

| Patient | ‚stop a drug' accepted? |  | ‚start a drug‘ accepted? |  | ‚change in dose‘ accepted? |  |
| --- | --- | --- | --- | --- | --- | --- |
|  | yes | no | yes | no | yes | no |
|  |  |  |  |  |  |  |
|  |  |  |  |  |  |  |
|  |  |  |  |  |  |  |
|  |  |  |  |  |  |  |
|  |  |  |  |  |  |  |
|  |  |  |  |  |  |  |
|  |  |  |  |  |  |  |
|  |  |  |  |  |  |  |
|  |  |  |  |  |  |  |
|  |  |  |  |  |  |  |
|  |  |  |  |  |  |  |
|  |  |  |  |  |  |  |
|  | 1 | 1 | 2 | 1 |  |  |
|  |  |  |  |  |  |  |
|  | 1 | 2 |  |  | 1 | 1 |
|  | 3 |  | 5 |  |  | 1 |
|  | 2 | 1 | 1 | 3 | 2 | 1 |
|  | 1 | 1 | 1 |  | 2 |  |
|  |  | 1 |  |  | 3 |  |
|  | 3 | 3 |  | 1 | 2 | 1 |
|  |  | 2 | 1 | 1 |  | 1 |
|  |  |  |  |  |  | 1 |
|  |  | 2 |  | 1 |  | 2 |
|  | 3 | 1 | 3 | 1 |  |  |
|  | 1 | 3 | 3 |  |  | 1 |
|  | 1 | 2 |  | 3 | 1 |  |
|  | 1 | 1 | 1 | 1 | 1 |  |
|  | 1 |  |  |  | 3 | 1 |
|  | 1 |  | 2 |  | 3 |  |
|  | 1 | 2 | 4 | 3 |  |  |
|  |  | 4 | 4 | 1 | 1 | 1 |
|  |  |  | 3 |  | 1 |  |
|  | 2 | 1 | 4 |  |  |  |
|  |  | 1 | 4 | 2 |  | 1 |
|  | 1 |  | 3 |  | 2 |  |
|  |  |  |  |  |  |  |
|  |  |  |  |  |  |  |
|  | 5 | 1 | 3 | 1 | 1 |  |
|  |  |  |  |  |  |  |
|  |  |  |  |  |  |  |
|  |  |  |  |  |  |  |
|  | 2 |  |  |  | 2 |  |
|  |  |  |  |  |  |  |
|  | 1 | 3 |  |  | 1 |  |
|  | 2 | 1 |  |  | 1 |  |
|  | 1 | 1 | 2 | 2 | 2 | 1 |
|  | 1 | 1 |  | 1 | 1 |  |
|  | 3 | 1 | 3 |  | 2 |  |
|  | 3 | 2 | 1 |  | 2 |  |
|  | 1 | 1 | 1 | 1 |  |  |
|  |  | 1 | 2 |  | 1 |  |
|  | 1 | 2 | 1 | 3 | 1 |  |
|  |  |  |  |  | 1 |  |
|  | 1 |  |  |  |  |  |
|  |  |  |  |  |  |  |
|  |  |  | 1 |  |  |  |
|  | 1 | 1 |  |  |  |  |
|  | 1 | 1 | 2 |  | 2 |  |
|  | 1 | 1 | 1 | 1 |  |  |
|  |  |  | 1 |  |  |  |
|  |  |  |  |  |  |  |
|  |  |  |  |  |  |  |
|  |  |  |  |  |  |  |
|  |  |  |  |  |  |  |
|  |  |  |  |  |  |  |
|  |  |  |  |  |  |  |
|  |  |  |  |  |  |  |
|  |  |  |  |  |  |  |
|  | 2 |  | 1 |  |  | 1 |
|  | 1 |  | 1 |  |  |  |
|  |  |  |  |  |  |  |
|  |  |  |  |  |  |  |
|  | 1 |  | 1 |  |  |  |
|  |  |  |  |  |  |  |
|  |  | 2 |  | 1 | 1 | 2 |
|  |  |  |  |  |  |  |
|  | 1 |  |  |  |  |  |
|  |  | 1 |  | 5 | 1 |  |
|  | 1 | 1 | 1 |  | 3 | 1 |
|  |  | 4 | 3 | 4 | 1 |  |
|  | 1 |  |  | 4 |  | 2 |
|  |  | 1 |  | 3 |  | 1 |
|  | 1 | 5 | 1 |  |  | 1 |
|  |  | 1 |  | 4 | 3 |  |
|  | 2 |  | 1 | 2 | 4 | 2 |
|  | 1 | 1 | 1 | 3 |  |  |
|  | 2 |  | 6 |  |  |  |
|  | 6 |  | 8 |  | 2 |  |
|  |  |  |  |  |  |  |
|  |  |  |  |  |  |  |
|  |  |  |  |  | 1 | 1 |
|  | 3 |  | 1 |  | 1 |  |
|  |  | 4 | 2 |  | 3 | 3 |
|  | 1 |  | 1 | 1 |  | 1 |
|  |  | 4 | 1 | 2 | 1 |  |
|  |  |  |  |  |  |  |
|  | 4 | 4 | 3 | 1 | 2 | 2 |
|  | 2 |  |  |  |  |  |
|  | 2 | 1 |  | 2 |  | 3 |
|  |  |  |  |  |  |  |
|  | 1 |  | 1 |  |  |  |
|  | 3 | 3 | 5 | 1 |  | 2 |
|  | 1 | 1 |  | 1 | 2 | 1 |
|  | 1 | 1 | 1 | 3 |  | 1 |
|  | 1 |  |  |  | 3 |  |
|  | 1 | 3 | 1 | 4 |  |  |
|  |  | 2 |  | 2 |  | 3 |
|  |  | 3 |  | 1 | 2 |  |
|  |  | 2 |  | 1 | 1 |  |
|  | 3 | 1 |  | 2 | 3 | 1 |
|  | 1 | 2 | 1 | 4 |  | 1 |
|  | 3 |  | 1 | 2 | 2 | 2 |
|  | 2 |  | 3 | 1 | 2 | 1 |
|  | 2 | 4 |  | 5 |  | 2 |
|  | 1 | 2 |  | 2 | 3 |  |
|  | 1 |  | 1 | 1 | 2 | 1 |
|  | 2 | 3 | 2 | 3 |  |  |
|  |  |  |  |  | 1 | 1 |
|  | 2 | 3 | 4 | 1 | 1 | 2 |
|  | 1 |  | 2 | 1 | 1 |  |
|  |  |  | 4 |  |  |  |
|  | 3 | 1 | 2 | 2 | 3 |  |
|  | 2 | 2 | 2 | 4 |  | 1 |
|  | 6 | 1 | 1 |  | 2 |  |
|  | 1 | 2 |  | 4 | 1 |  |
|  | 4 | 1 | 2 | 3 | 1 |  |
|  | 2 | 1 | 2 | 2 | 1 | 1 |
|  | 1 | 1 |  | 4 |  | 1 |
|  |  |  |  |  |  |  |
|  |  |  |  |  |  |  |
|  |  |  |  |  |  |  |
|  |  |  |  |  |  |  |
|  | 1 |  | 1 |  | 2 |  |
|  | 1 |  |  |  | 2 | 2 |
|  | 3 | 2 |  | 1 | 1 |  |
|  | 1 |  |  |  |  | 1 |
|  |  |  |  |  | 1 | 1 |
|  |  | 3 |  |  | 1 |  |
|  | 1 |  |  |  | 1 |  |
|  |  | 1 |  |  |  | 1 |
|  |  |  |  | 2 | 1 |  |
|  | 3 | 2 | 2 | 4 | 1 |  |
|  |  |  |  |  | 1 | 1 |
|  |  |  |  |  |  |  |
| total per item | 133 | 121 | 129 | 120 | 104 | 60 |
|  |  |  |  |  |  |  |
| total number  rated | 667 |  |  |  |  |  |
