# Supplementary material for: Priority Setting and Influential Factors on Acceptance of Pharmaceutical Recommendations in Collaborative Medication Reviews in an Ambulatory Care Setting – Analysis of a Cluster Randomized Controlled Trial (WestGem-Study)
Source: PLoS One. 2016 Jun 2;11(6):e0156304. doi: 10.1371/journal.pone.0156304 (PMC4890849; doi:10.1371/journal.pone.0156304)
Supplement: S1 Protocol — (PDF) [file pone.0156304.s004.pdf]

Effect evaluation of an interprofessional medication therapy management approach for multimorbid patients in primary care: a cluster-randomized controlled trial in community care (WestGem study protocol)

Olaf Rose[1,6,]
Email: rose@elefantenapo.de

Corinna Schaffert[2]
Email: Schaffert@wiwi.uni-wuppertal.de

Kathrin Czarnecki[2]
Email: CzarneckiK@wiwi.uni-wuppertal.de

Hugo S. Mennemann[3]
Email: h.mennemann@fh-muenster.de

Isabel Waltering[4]
Email: waltering@pharmd.de

Stefanie Hamacher[5]
Email: stefanie.hamacher@uni-koeln.de

Moritz Felsch[5]
Email: moritz.felsch@uni-koeln.de

Lena Herich[5]
Email: lena.herich@uni-koeln.de

Juliane Köberlein[2*,]
*Corresponding author
Email: koeberlein@wiwi.uni-wuppertal.de

[1]University of Bonn, Department of Clinical Pharmacy, An der Immenburg 4, 53121 Bonn Germany.
[2]Centre of Health Care Management and Public Health, Schumpeter School of Business and Economics, University of Wuppertal, Wuppertal, Germany.
[3]Muenster University of Applied Science, Robert-Koch-Str. 30, 48149 Muenster, Germany.
[4]University of Muenster, Department of Pharmacy, Correnstr. 48, 48149 Muenster.
[5]Institute of Medical Statistics, Informatics and Epidemiology, University of Cologne, Kerpener Str. 62, 50937 Cologne, Germany.
[6]Elefanten-Apotheke gegr 1575, Steinstr. 14, 48565 Steinfurt, Germany.

Abstract

Background: Pharmaceutical practice worldwide is developing towards patient care. Medication Review (MR) and Medication Therapy Management (MTM) are evolving as the most prominent services in pharmaceutical care and have a strong potential to provide a large benefit for patients and society. MTMs can only be performed in an interprofessional, collaborative setting. Several international studies have explored the effects of a MTM on the quality of therapy and costs. For Germany the data is still deficient. This study aims to provide data on the effects of an interprofessional MTM regarding quality of therapy, quality of life, costs and cost-effectiveness.

Method/Design: The study is designed as a cluster-randomized controlled trial in primary care, involving 12 outpatient clinics (clusters) and 165 patients. Primary care units are allocated to interventions using a Stepped Wedge Design. All units are initially assigned to the control group. After a 6 month observation period, general practitioners (GP) are randomly allocated to one of three groups and the interprofessional medication therapy management approach is implemented sequentially per each group with a lag of 3 months between. The primary outcome is the change in the quality of therapy measured by the MAI (Medication Appropriateness Index). Secondary outcomes include changes in the number of drug related problems, medication complexity, changes in drug-adherence, changes in health-status and function, quality of life, direct costs and the incremental cost-effectiveness ratio. The acceptance of the interprofessional Medication Therapy Management approach is assessed by qualitative methods.

Discussion:
The patient interview and brown bag review are activities, typically provided by the pharmacist. In this trial the patient is blinded to the pharmacist. The strength of having the patient blinded to the pharmacists is to exclude skepticism of the patient toward unknown pharmacies, which might be a major confounder in a regional and community setting. A weakness is that some patient related data might reach the pharmacists in a way, which might differ from self-acquired data.

Trial registration: Current controlled trials ISRCTN41595373

Keywords: polypharmacy, interprofessional, medication therapy management, medication review, multimorbidity, complex intervention, primary care

Background

Polypharmacy

The care of patients with multiple chronic diseases entails many challenges, in particular related to higher than average coordination and medication complexity. In an European study Fialova et al found that 51% of participating patients take more than six prescribed medications per day [1].
Overall, 20% of GPs' patients older than 65 years receive 60% of all prescribed drugs [2]. In fact, polypharmacy comes along with frequently undesirable consequences, such as increased risk of inappropriate drug use, under-use of effective treatments, medication errors, drug interactions, poor patient compliance, and adverse drug reactions [3]. Regarding this, medication management provided by pharmacists may overcome these challenges [4, 5].

Current interventions and MTM approaches

Pharmaceutical practice worldwide is currently developing towards patient care. Pharmaceutical care has been promoted by Hepler and Strand of the University of Florida in 1990 and has been redefined by the PCNE in 2013/2014 [6, 7]. According to the PCNE definition, pharmaceutical care covers numerous activities to "optimize medicines use and improve health outcomes". Certain care aspects, like enhanced patient education have been well described and studied: Jalal et al. found patient education, provided by pharmacists beneficial in cardiovascular diseases [8], Schmiedel et al. recently found that patient education can reduce the risk to acquire diabetes [9]. Several studies could support the efficacy of patient counseling on drug-adherence [10] or patient skills in handling drug-devices [11-13].
The WHO and FIP have promoted a patient centered approach by publishing a handbook in 2006 [14]. Medication Review (MR) and Medication Therapy Management (MTM) are evolving as the most prominent services in pharmaceutical care and have a strong potential to provide a large benefit for patients and society. A comprehensive MTM can only be performed in an interprofessional setting [15]. Several international studies have explored the effects of a MTM on the quality of therapy and costs [16, 10, 17, 18]. A systematic review of Nkansah et al. found that the available trials are varying in study design and endpoints and hardly can be compared to services of other health care providers. Hence further studies on Medication Management are desired [19].
The impact of pharmaceutical services widely differs among societies with the setting of the national health care system. Differences in education and collaboration as well as structures and barriers between professions lead to a variety of possible outcomes. For Germany the data supporting a MTM is still deficient.

Novel aspects of the interprofessional medication therapy management approach

Most studies on MTM are examining certain effects of the intervention of the participating pharmacists and are evaluated by themselves. Pharmaceutical aspects, like a change in drug-adherence or a reduction in drug related problems are assessed [20-22].
Interventions can only reach the patient if they are approved by the decision maker, the general practitioner (GP) or primary care provider (PCP). A consensus between all health care providers is likely to support the therapy. The WestGem-study has a pronounced focus on interprofessional cooperation and collaboration. It might be one of the first Medication

Therapy Management studies combining three participating health care professions, consisting of physicians, pharmacists and home-care specialists. The interprofessional approach combines case management routines of the home-care specialists at the patient-site with information gained during the advanced Medication Review by specialised and clinical experienced study pharmacists.
The development of the approach was based on the Medical Research Council (MRC) guideline for the development and evaluation of randomized controlled trials [23, 24]. It was piloted with a group of seven GPs, two pharmacists and two home-care specialists.

Study aim and objectives

The aim of this randomized controlled trial is to evaluate the application of an interprofessional collaborative Medication Therapy Management approach in multimorbid patients, receiving multiple systemic available drugs. The evaluation refers to the extent of improvement in the quality of drug therapy through examination of drug related problems (DRPs) or drug related events and suggestions on optimizing drug use to reach therapeutic goals. Several tools are used to assess the patients' drug therapy and home-care needs.
Part of the complex intervention might be the removal of inappropriately prescribed medication, disclosure of drug related problems and prescribing cascades, assessing drug-drug interactions, determination of therapeutic goals, evaluation of pain management, the assessment of chief complaints and quality of life and a reflection of costs and cost-effectiveness of this complex intervention under terms of routine care.

Methods

Primary objective

The primary objective of this study is to determine whether the complex intervention would change the quality of medication therapy determined by the MAI (medication appropriateness index) [25, 26] in comparison to standard care. The intervention focuses on multimorbid patients receiving polypharmacy. It is done supplementary to standard care.

Secondary objectives

Secondary outcomes include changes in the number of drug related problems, classified according to PCNE version 6.2, medication complexity, measured by the MRCI [27], changes in adherence (measured by the Morisky-score [18]), changes in health-status and function, quality of life, direct and indirect costs, and the incremental cost-effectiveness ratio.

Setting

The study is conducted in a community and outpatient primary care setting in two model regions in North Rhine-Westphalia, located in Western Germany. We have chosen regions with different network structures, which enables us to measure performance and outcomes of the interprofessional medication therapy management approach while taking setting specific influence factors into account.

Outpatient health care in *region A* is organised as a network including GPs (n ≈ 15) and medical specialists (n ≈ 18). Outpatient health care in *region B* does not present in any network structure (number of available GPs in this area is ≈ 55). Local GPs of the *model regions* are contacted by an informative letter, briefly explaining details of the planned intervention and essential study tasks. After written confirmation of participation in the study by interested GPs, the project assistants visit the participating GPs personally to explain the study design, provide study instruction and gain the GPs' agreement to participate. In this context, a monetary incentive is provided to positively influence participation and cooperation [28-30]. Considering the experience of previous research for studies with relatively high workload for documentation, patient recruitment and study intervention, we expect a response-rate of 6-10% [31, 32] for region B. Furthermore we expect, that the existing network structure in region A will generate a higher response rate.

Study design

The study is designed as a cluster-randomized controlled trial, incorporating qualitative analysis. The qualitative analyses have been used during intervention development and piloting. Further qualitative methods will be applied to perform a process evaluation of the randomized trial and to assess the acceptance of the interprofessional Medication Therapy Management approach [33]. The study design is developed in line with the CONSORT statement extension to cluster RCT [34, 35]. The cluster design is chosen to avoid spillover effects across patients of the control and intervention groups. To ensure the feasibility and a high acceptance of the methodical cornerstones of the study, its essential aspects were piloted. Thereby the patients recruiting process, randomization routines, the applied documentation forms and data collection procedures are examined.

Participating GPs are allocated to one of three study arms by undertaking a cluster randomization on the level of the primary care units following a Stepped Wedge Design [36]. In this sense all GPs are initially assigned to the control group. After a 6 month observation period, general practitioners are randomly allocated to one of the three groups and the interprofessional Medication Therapy Management approach is implemented sequentially per each group with a lag of 3 months (see Fig. 1).

Patients recruited by the GPs receive standard care during the control period. After the implementation of the MTM-approach a first comprehensive Medication Review is performed by the pharmacists as well as a support by the home-care specialists using case management techniques (see Tab. 1). The Medication Review and the personal support by the home-care specialists are repeated after 6 months. The GP is free to accept or deny any suggestions made by the pharmacists and health-care specialists and keeps his or her unrestricted individual freedom of choice at all time during the study period. Primary and secondary endpoints are assessed at baseline and 3, 6, 9, 12 and 15 months post-baseline. Patient groups are compared with respect to their treatment response within the study period.

Primary hypothesis

It is proposed that patients receiving the interprofessional MTM show a significantly lower MAI score compared to patients receiving standard care [25, 26]. Therefore the study evaluates the primary null hypothesis that an interprofessional Medication Therapy Management approach has no influence on the quality of drug therapy.

Randomization

Participating clinics were randomly allocated to one of three study arms. A biometrician who is not involved in the field work, randomly selects the clinics. To avoid changes in physician’s prescription behavior, random lists remain concealed until each allocation date.

Ethical approval

The study protocol was approved by the responsible local Ethics Committee in the Westphalia-Lippe region (approval number AKZ-2013-292-f-s) and will be conducted to the principles of the Declaration of Helsinki.

Study population

Patient recruitment

The recruitment of the patients is carried out by the participating GPs. To avoid selection bias, patients' inclusion comprises of two steps. At first, all patients are screened for the defined in- and exclusion criteria. GPs systematically identify patients who are generally eligible for study inclusion. Potential study patients are listed in alphabetic order and are numbered consecutively (basic population). In a second step GPs add gender, age, and conditions (diagnoses) to this list. At a later date physicians provide a pseudonymous version of the recruitment list to the biometricians who determine a random sample of patients. These participants are informed about the study by their GP and asked to participate. After giving informed consent, baseline documentation forms and questionnaires are completed. For every patient of the sample list who declines participation, a new patient is drawn from the basic population pool. For potential study patients who decline participation a sensitivity analysis is planned at the end of the trial to determine whether this sample differs according to age, gender and structure of acute, as well as chronic conditions.
Figure 2 illustrates the flowchart for recruitment of primary care units and patients.

Inclusion and exclusion criteria

The following inclusion criteria are defined:

- patients age ≥ 65 years,
- at least 3 chronic diseases out of two organ systems with one being a cardiovascular disease (for the identification of relevant chronic diseases the list published by van den Bussche et al. 2011 was used [37]) ,
- at least 1 visit to the Primary Care Provider in each of the last 3 quarters,
- at least 5 chronic systemic available medications,
- (signed informed consent).

Patients with an insufficient ability to speak or read German, participation in other studies at the present time and with the existence of severe illnesses that might be lethal within 12 months according to the GP's judgment are excluded.

Sample size and power calculation

Sample size calculation for the stepped wedge design is based on Woertman et al. [38]. Because there were no studies investigating the effect of Medication Management an effect size of 0.25 is considered as clinically and socially relevant. Based on this assumption and using a two-tailed t-test with statistical power of 80% and significance level alpha = 0.05 a total unadjusted sample size of N = 502 is needed. An assumption of 20 patients per practice and little correlation between the clusters (ICC = 0.05) leads to a design factor of 0.383 in the present step wedge model. Adjusting the sample size with the design factor and considering a maximum drop-out rate of 20% the final sample size is calculated to N = 240.

Data collection

The main data collection comprises of paper-pencil questionnaires and documentation forms for chart reviews as well as telephone interviews with patients. After obtaining written informed consent, patients are registered in the study coordinating center of the Department of Health Care Management and Public Health Wuppertal (Germany).
Patients are asked to fill in a questionnaire. GPs document additional data from patients' chart and assess the current clinical status of the patient (e.g. blood pressure, Tinetti test, mini-mental state examination).
Patient questionnaire, chart review and telephone interview are performed at baseline (t0/t1), 3 months post-baseline (t2), 6 months (t3), 9 months post-baseline (t4), 12 months post-baseline (t5) and 15 months post-baseline (t6). The baseline documentation includes a retrospective assessment period over six month.
The pharmacists' and home-care specialists' assessment and reporting instruments are evaluated to gain the following information:

- medication appropriateness index (MAI) [25, 26];
- medication regimen complexity index (MRCI) [27];
- potential inadequate medication,
- drug related problems,
- possible medical interactions,
- number of taken over-the-counter medication,
- deviation between the GPs prescribing and the brown bag,
- patient's therapeutic goals,
- experienced side effects and
- further interventions suggested during Medication Review.

Within semi-standardized and guideline-based telephone interviews GPs are interviewed twice (at the beginning and at the end of study) to discuss their expectations toward and experiences with the interprofessional Medication Therapy Management approach. To gain influencing factors on physician and study site level, a standardized questionnaire is used that was developed in a previous study [39].

Outcome measures

Primary outcome

The primary objective of this study is to determine whether the complex intervention can change the quality of medication therapy. Therefore specialised study pharmacists measure the quality of medication therapy at baseline (t0/t1), 3 months post-baseline (t2), 6 months

(t3), 9 months post-baseline (t4), 12 months post-baseline (t5) and 15 months post-baseline (t6) by applying the MAI, a validated instrument with good intra-rater and inter-rater reliability, as well as face and content validity [40, 25, 41, 26].
The MAI is an implicit (judgment-based) process measure, which assesses ten elements of prescribing: indication, effectiveness, dose, correct directions, practical directions, drug-drug interactions, drug-disease interactions, duplication, duration and cost. To standardize the rating process, the index has operational definitions and instructions. The ratings result in a weighted score that serves as a summary measure of prescribing appropriateness [25, 26, 22]. We assume that an increase in appropriate polypharmacy would improve indicators of morbidity such as reduction in adverse drug events (ADEs) or hospital admissions, which are both also followed in this study. Furthermore we suggest that appropriate prescribing would positively influence mortality as well as morbidity and quality of life.
The choice for the process measure MAI as primary outcome parameter was made in consideration of a current Cochrane review [42] determining, which interventions are effective in improving the appropriate use of polypharmacy, reducing medication-related problems in older people and avoiding hospital admissions. The review reported that the majority of the eligible studies (seven out of eleven) used the validated MAI as a primary outcome. To determine whether the MAI can predict patient relevant outcomes and to assess its predictive validity, additional analyses are planned. More specifically, it will be examined whether any changes in the primary outcome measure result in changes in the secondary outcomes.

Secondary outcomes

Additional information regarding the quality of medication therapy will be obtained from assessment instruments used by the study pharmacists within their Medication Review. These documents include

- the number of drug related problems, classified according to PCNE version 6.2,
- medication complexity, measured by the medication complexity index (MRCI) [27],
- the prevalence of inadequate medication, using the PRISCUS-list [2] for assessment (see table 2)

Data according to over-the-counter medication, drugs prescribed by specialists, reported side effects, patients goals of therapy, patient reported medication and the risk of falling are provided by the assessment instrument of the home-care specialists.
The medication adherence is determined according to Morisky and MARS. Health related quality of life is assessed using the Short Form 12 Health Questionnaire (SF-12) and the EuroQoL instrument EQ-5D. We are documenting these instruments using a paper-based patient questionnaire as well as during the telephone interviews. Discrepancies between the two measures will be analysed.
Other secondary outcome parameters are: depression (PHQ-9), self-rated health, activities of daily living (ADL/iADL), mobility (FFB-Mot), pain (GCPS), mortality, hospital admissions, number and type of accepted medication proposals, social participation (F-SOZU K14), health care utilization and total healthcare costs.
Additionally quantitative (process documentation instruments) and qualitative (focus groups, narrative interviews) process evaluation is conducted to identify possible barriers of implementation and to detect needs to modify the intervention.

Statistical analyses

Baseline and demographic characteristics are analysed descriptively (number of valid cases,

mean, standard deviation, minimum, median, lower and upper quartile, maximum for quantitative variables and number and proportions for qualitative variables).
The statistical analyses of the full-analysis set will follow the intention-to-treat (ITT) principle. This dataset includes all trial subjects who are randomized, meet all inclusion and exclusion criteria, have signed informed consent and have at least a baseline MAI score. Supportive analyses will be performed for the per-protocol (PP) population that includes all trial subjects of the ITT set who were treated as randomized and have at least two MAI scores after changing from control to intervention.
The primary hypothesis will be evaluated by a mixed model with treatment group and time as fixed effects and clustering structure as random effect. Significance level is set to alpha = 5% (two-sided). It is assumed that values are missing at random (MAR) therefore no values need to be replaced in the mixed model. Secondary outcomes are analysed analogously. Further analyses are performed exploratory. In the sensitivity analysis missing values are replaced with last observation carried forward (LOCF).
Subgroup analyses will be performed with respect to age, gender, migrant status, social bonding and complexity of morbidity structure.

Cost-effectiveness
The cost-effectiveness analysis is performed from a societal perspective. Therefore direct costs are calculated. We will exclude indirect costs as we assume that these are not relevant for our included patient group. To calculate the documented resource utilization we use administrative and market prices.
As effect measure QALYs are calculated from the EQ-5D and as the point estimate the incremental costs per QALY will be determined.
To take uncertainty into account a cost-effectiveness-acceptability-curve is computed, using non-parametric bootstrapping methods. Furthermore, a net-monetary benefit regression analysis will be performed [43].

Methods against bias and for quality assurance

To ensure data quality and to avoid missing data or processes which are not adherent with the study protocol, clinical research associates visit study sites for clinical monitoring. Furthermore several routines, like a data handling report, are established to prevent or detect incorrect as well as inconsistent data entry and incomplete data. In this regard a random sample of paper-pencil questionnaires is compared with the data entries in the database. Additionally, regular training sessions are done.

Intervention

Interprofessional Medication Therapy Management approach

Pharmacists perform a comprehensive Medication Review (PCNE type 3). They receive the patient data from the GP and from the home-care specialists (see table 1). All data reaching the pharmacists is anonymised. Besides their own assessments, home-care specialists perform several assessments at the patients' site for the pharmacists. At these visits a “brown bag review” of the drugs in use by the patient is performed as well as an intense patient interview, covering a list with all the questions a pharmacist would ask a patient including side effects,

difficulties in handling, adherence, nutrition, dizziness, social support and else. The home-care specialists furthermore evaluate the demand of the patient for home-care devices or products, social and financial support and identify tripping hazards and potential risks. The pharmacists transfer all the provided data to a calculation sheet for statistical purposes and develop a message to the GP based on the SOAP-note form.

In a first attempt, the data of the brown bag review is compared to the medication plan of the GP. Deviations are registered and possible explanations are assumed and added. Based on the diagnoses, the laboratory data and the reported complaints, individual therapeutic goals are generated and the glomerular filtration rate (GFR) is calculated using the Cockroft-Gault equation. The pharmacotherapy is assessed on:

- drug-drug interactions,
- contraindications,
- suitability to reach the therapeutic goals,
- guideline accordance,
- difficulties in handling the drugs,
- problems of timing and drug-food interactions,
- indications without a drug,
- drugs without an indication,
- duplications,
- toxicity/dose/geriatric appropriateness,
- drug monitoring,
- appropriateness of lengths of therapy,
- side effects and
- costs.

Depending on the patient further problems are assessed. Pharmacists are discussing favourable interventions and are generating a new medication plan. Suggestions for monitoring parameters and patient counseling are expressed. A SOAP note to the GP is written. Estimations on the disease related and drug depending falling risk are provided to the home-care specialists.

Intervention for the control group

At control condition patients receive standard treatment by their GP and other health care providers within the regular German health care system.

Discussion

Interprofessional Medication Therapy Management for multimorbid patients receiving polypharmacy may improve quality of medication therapy and outcomes. MTMs have increasingly been recognized as a resource in overcoming shortages in primary care [44].
The study investigates a new type of collaboration between GPs, pharmacists and home-care specialists in outpatient care and combines case management routines of the home-care specialists at the patient-site with information gained during the advanced Medication Review by specialised and clinical experienced study pharmacists.

The emphasis on interprofessional cooperation and collaboration with the participation of physicians, pharmacists and home-care specialists has a greater potential to show an improvement in the interventions compared to drug safety and therapy management programs by a single profession alone. It is strongly believed that the future of optimising a patient's therapy as well as reducing patient's drug risks can only be provided by a collaborative health care team consisting of different professions. In this trial the home-care specialists provide their patient oriented insight to the pharmacists and physicians. They suggest interventions relating to patient care directly to the GPs. Pharmacists perform the Medication Review not only with a focus on drug safety but also on the quality of therapy, therapeutic outcomes and patient goals. The GPs outweigh all these suggestions, perform several assessments with the patient and are free to choose the best therapeutic alternatives, based on their own judgment.

The patient interview and brown bag review are activities, typically provided by the pharmacist [45, 46]. All investigations at the patient are performed by the physician and the home-care specialists, according to a standardized and comprehensive list. The pharmacists are referred to as research pharmacists at a different location who receive only anonymised data. The reasons for this blinded approach are various. In a regional setting patients do not want to see an unknown pharmacist to receive all their data. There might be strong relations of the patients toward a different pharmacy. Personal relations and social ties to a pharmacist are very important for elderly patients in Germany. There might be a strong skepticism of the patient toward foreign pharmacies and pharmacists, which might be a major barrier and confounder in a regional and community setting. As a MTM is unknown to most participating GPs, they might as well hesitate to share their data with a local pharmacist as they feel controlled by them at this stage. At introductory interviews patients and physicians clearly preferred to stay anonymous. As pharmacists in this trial do not need a personal meeting with the patients at the site, a strength of being anonymous is that highly trained pharmacotherapy experts from different areas in Germany can enter the team. The considered study-pharmacists need to be specialists in clinical pharmacy and pharmacotherapy with additional clinical education, experience and training. For participation in this complex study therapeutic knowledge and scientific experience is indispensable. Handling of the MAI needs further training and skills to meet research standards. Based on these criteria a nationwide team of pharmaceutical researchers is recruited to build the team of MTM-experts. A weakness of being anonymous is that some patient related data might reach the pharmacists in a way, which might differ from self-acquired data.

List of abbreviations

DRP	Drug related problem
FIP	International Pharmaceutical Federation
GFR	Glomerular filtration rate
GP	General practitioner
ICC	Intraclass correlation
ITT	Intention-to-treat
LOCF	Last observation carry forward
MAI	Medication appropriateness index
MARS	Medication adherence report scale
MR	Medication Review
MRC	Medical research council
MRCI	Medication regimen complexity index
MTM	Medication Therapy Management
NCEP	National cholesterol education program
PCNE	Pharmaceutical Care Network Europe
PCP	Primary care physician
PP	Per protocol
QALY	Quality-adjusted life years
RCT	Randomised controlled trial
SOAP	Acronym for subjective, objective, assessment and plan
WHO	World Health Organisation

Acknowledgements

We thank these GPs and their medical assistants, home-care specialists and pharmacists who have already consented to participate in this study.

Authors' contribution

OR, JKN, IW, CS and HM developed the interventions and the study protocol. MF and SH developed the statistical analyses plan. KC contributed to the development of intervention and piloting. CS and KC managed the recruitment process. JKN and OR wrote the first draft of the manuscript. IW, CS, KC, MF, SH, LH and HM critically revised it. All authors read and approved the final manuscript.

Competing interests

The authors declare that they have no competing interests. Olaf Rose is a prevalent speaker at conferences and has received tuition fees for speeches at conventions or for printed matter by Bayer, Boehringer Ingelheim, Medac and MSD and by the publishing houses DAV, DAZ, PNN, Govi and Wort und Bild. He denies any corporate influence on his research activities or on this study.

Funding

The study is granted by the European Union and the German State of North-Rhine-Westphalia as part of the competitive call IuK&Gender Med.NRW.

References

1. Fialova D, Topinkova E, Gambassi G, Finne-Soveri H, Jonsson PV, Carpenter I et al. Potentially inappropriate medication use among elderly home care patients in Europe. JAMA. 2005;293(11):1348–58. doi:10.1001/jama.293.11.1348.
2. Holt S, Schmiedl S, Thurmann PA. Potentially inappropriate medications in the elderly: the PRISCUS list. Deutsches Arzteblatt international. 2010;107(31-32):543–51. doi:10.3238/arztebl.2010.0543.
3. Gandhi TK, Weingart SN, Borus J, Seger AC, Peterson J, Burdick E et al. Adverse drug events in ambulatory care. The New England journal of medicine. 2003;348(16):1556–64. doi:10.1056/NEJMsa020703.
4. Barnett MJ, Frank J, Wehring H, Newland B, VonMuenster S, Kumbera P et al. Analysis of pharmacist-provided medication therapy management (MTM) services in community pharmacies over 7 years. Journal of managed care pharmacy : JMCP. 2009;15(1):18–31.
5. Isetts BJ, Schondelmeyer SW, Artz MB, Lenarz LA, Heaton AH, Wadd WB et al. Clinical and economic outcomes of medication therapy management services: the Minnesota experience. Journal of the American Pharmacists Association : JAPhA. 2008;48(2):203-11; 3 p following 11. doi:10.1331/JAPhA.2008.07108.
6. Allemann SS, van Mil JWF, Botermann L, Berger K, Griese N, Hersberger KE. Pharmaceutical care: the PCNE definition 2013. International journal of clinical pharmacy. 2014;36(3):544–55. doi:10.1007/s11096-014-9933-x.
7. Hepler CD, Strand LM. Opportunities and responsibilities in pharmaceutical care. American journal of hospital pharmacy. 1990;47(3):533–43.
8. Jalal ZS, Smith F, Taylor D, Patel H, Finlay K, Antoniou S. Pharmacy care and adherence to primary and secondary prevention cardiovascular medication: a systematic review of studies. European Journal of Hospital Pharmacy: Science and Practice. 2014;21(4):238–44. doi:10.1136/ejhpharm-2014-000455.
9. Schmiedel K, Mayr A, Fiessler C, Schlager H, Friedland K. Effects of the Lifestyle Intervention Program GLICEMIA in People at Risk for Type 2 Diabetes: A Cluster-Randomized Controlled Trial. Diabetes care. 2015;38(5):937–9. doi:10.2337/dc14-2206.
10. Lee JK, Grace KA, Taylor AJ. Effect of a pharmacy care program on medication adherence and persistence, blood pressure, and low-density lipoprotein cholesterol: a randomized controlled trial. JAMA. 2006;296(21):2563–71. doi:10.1001/jama.296.21.joc60162.
11. Hammerlein A, Muller U, Schulz M. Pharmacist-led intervention study to improve inhalation technique in asthma and COPD patients. Journal of evaluation in clinical practice. 2011;17(1):61–70. doi:10.1111/j.1365-2753.2010.01369.x.
12. Kjeldsen LJ, Bjerrum L, Dam P, Larsen BO, Rossing C, Sondergaard B et al. Safe and effective use of medicines for patients with type 2 diabetes - A randomized controlled trial of two interventions delivered by local pharmacies. Research in social & administrative pharmacy : RSAP. 2015;11(1):47–62. doi:10.1016/j.sapharm.2014.03.003.

13. Schulz M, Verheyen F, Muhlig S, Muller JM, Muhlbauer K, Knop-Schneickert E et al. Pharmaceutical care services for asthma patients: a controlled intervention study. Journal of clinical pharmacology. 2001;41(6):668–76.

14. Developing pharmacy practice-A focus on patient care, Handbook 2006. World Health Organization and International Pharmaceutical Federation. http://www.who.int/mediacentre/news/new/2006/nw05/en/ ed.

15. van Mil JWF, Schulz M, Tromp TFJD. Pharmaceutical care, European developments in concepts, implementation, teaching, and research: a review. Pharmacy world & science : PWS. 2004;26(6):303–11.

16. Isetts BJ, Brown LM, Schondelmeyer SW, Lenarz LA. Quality assessment of a collaborative approach for decreasing drug-related morbidity and achieving therapeutic goals. Archives of internal medicine. 2003;163(15):1813–20. doi:10.1001/archinte.163.15.1813.

17. Pringle JL, Boyer A, Conklin MH, McCullough JW, Aldridge A. The Pennsylvania Project: pharmacist intervention improved medication adherence and reduced health care costs. Health affairs (Project Hope). 2014;33(8):1444–52. doi:10.1377/hlthaff.2013.1398.

18. Ramanath K, Balaji D, Nagakishore C, Kumar SM, Bhanuprakash M. A study on impact of clinical pharmacist interventions on medication adherence and quality of life in rural hypertensive patients. Journal of young pharmacists : JYP. 2012;4(2):95–100. doi:10.4103/0975-1483.96623.

19. Nkansah N, Mostovetsky O, Yu C, Chheng T, Beney J, Bond CM et al. Effect of outpatient pharmacists' non-dispensing roles on patient outcomes and prescribing patterns. The Cochrane database of systematic reviews. 2010(7):CD000336. doi:10.1002/14651858.CD000336.pub2.

20. Bregnhoj L, Thirstrup S, Kristensen MB, Bjerrum L, Sonne J. Combined intervention programme reduces inappropriate prescribing in elderly patients exposed to polypharmacy in primary care. European journal of clinical pharmacology. 2009;65(2):199–207. doi:10.1007/s00228-008-0558-7.

21. Clyne B, Bradley MC, Hughes CM, Clear D, McDonnell R, Williams D et al. Addressing potentially inappropriate prescribing in older patients: development and pilot study of an intervention in primary care (the OPTI-SCRIPT study). BMC health services research. 2013;13:307. doi:10.1186/1472-6963-13-307.

22. Spinewine A, Swine C, Dhillon S, Lambert P, Nachega JB, Wilmotte L et al. Effect of a collaborative approach on the quality of prescribing for geriatric inpatients: a randomized, controlled trial. Journal of the American Geriatrics Society. 2007;55(5):658–65. doi:10.1111/j.1532-5415.2007.01132.x.

23. Craig P, Dieppe P, Macintyre S, Michie S, Nazareth I, Petticrew M. Developing and evaluating complex interventions: the new Medical Research Council guidance. BMJ (Clinical research ed). 2008;337:a1655.

24. Craig P, Petticrew M. Developing and evaluating complex interventions: reflections on the 2008 MRC guidance. International journal of nursing studies. 2013;50(5):585–7. doi:10.1016/j.ijnurstu.2012.09.009.

25. Hanlon JT, Schmader KE, Samsa GP, Weinberger M, Uttech KM, Lewis IK et al. A method for assessing drug therapy appropriateness. Journal of clinical epidemiology. 1992;45(10):1045–51.

26. Samsa GP, Hanlon JT, Schmader KE, Weinberger M, Clipp EC, Uttech KM et al. A summated score for the medication appropriateness index: development and assessment of

clinimetric properties including content validity. Journal of clinical epidemiology. 1994;47(8):891–6.

27. Stange D, Kriston L, Langebrake C, Cameron LK, Wollacott JD, Baehr M et al. Development and psychometric evaluation of the German version of the Medication Regimen Complexity Index (MRCI-D). Journal of evaluation in clinical practice. 2012;18(3):515–22. doi:10.1111/j.1365-2753.2011.01636.x.

28. Edwards P RI, Clarke M, DiGuiseppi C, Pratap S, Wentz R, Kwan I. Increasing response rates to postal questionnaires: systematic review. BMJ. 2002;324(7347):1183. doi:10.1136/bmj.324.7347.1183.

29. Kellerman SE, Herold J. Physician response to surveys. A review of the literature. American journal of preventive medicine. 2001;20(1):61–7.

30. VanGeest JB, Johnson TP, Welch VL. Methodologies for improving response rates in surveys of physicians: a systematic review. Evaluation & the health professions. 2007;30(4):303–21. doi:10.1177/0163278707307899.

31. Bleidorn J VI, Wrede J, Dierks ML, Junius-Walker U. Keeping the Wire Hot with Calls? Recruiting Family Practices for a Health Care Research Project. ZfA.2012:61–8.

32. Güthlin C, Beyer M, Erler A, Gensichen G, Hoffmann B, Mergenthal K et al. Recruitment of Family Practitioners for Research. ZfA. 2012;88(4):173–81.

33. Lewin S, Glenton C, Oxman AD. Use of qualitative methods alongside randomised controlled trials of complex healthcare interventions: methodological study. BMJ (Clinical research ed). 2009;339:b3496.

34. Campbell MK, Elbourne DR, Altman DG. CONSORT statement: extension to cluster randomised trials. BMJ (Clinical research ed). 2004;328(7441):702–8. doi:10.1136/bmj.328.7441.702.

35. Campbell MK, Piaggio G, Elbourne DR, Altman DG. Consort 2010 statement: extension to cluster randomised trials. BMJ (Clinical research ed). 2012;345:e5661.

36. Brown CA, Lilford RJ. The stepped wedge trial design: a systematic review. BMC medical research methodology. 2006;6:54. doi:10.1186/1471-2288-6-54.

37. van den Bussche H, Koller D, Kolonko T, Hansen H, Wegscheider K, Glaeske G et al. Which chronic diseases and disease combinations are specific to multimorbidity in the elderly? Results of a claims data based cross-sectional study in Germany. BMC public health. 2011;11:101. doi:10.1186/1471-2458-11-101.

38. Woertman W, Hoop Ed, Moerbeek M, Zuidema SU, Gerritsen DL, Teerenstra S. Stepped wedge designs could reduce the required sample size in cluster randomized trials. Journal of clinical epidemiology. 2013;66(7):752–8. doi:10.1016/j.jclinepi.2013.01.009.

39. Köberlein J, Gottschall M, Czarnecki K, Thomas A, Bergmann A, Voigt K. General practitioners' views on polypharmacy and its consequences for patient health care. BMC family practice. 2013;14:119. doi:10.1186/1471-2296-14-119.

40. Bregnhoj L, Thirstrup S, Kristensen MB, Sonne J. Reliability of a modified medication appropriateness index in primary care. European journal of clinical pharmacology. 2005;61(10):769–73. doi:10.1007/s00228-005-0963-0.

41. Knight EL, Avorn J. Quality indicators for appropriate medication use in vulnerable elders. Annals of internal medicine. 2001;135(8 Pt 2):703–10.

42. Patterson SM, Cadogan CA, Kerse N, Cardwell CR, Bradley MC, Ryan C et al. Interventions to improve the appropriate use of polypharmacy for older people. The Cochrane database of systematic reviews. 2014;10:CD008165. doi:10.1002/14651858.CD008165.pub3.

43. Glick HA, Doshi JA, Sonnad SS, Polsky D. Economic evaluation in clinical trials. Second edition ed. Handbooks in health economic evaluation series.

44. Arya V, Pinto S, Singer J. Understanding awareness of pharmacist-led medication therapy management among primary care physicians in New York City, Part II: Findings and implications. The Journal of medical practice management : MPM. 2013;29(3):187–94.

45. Badowski SA, Rosenbloom D, Dawson PH. Clinical importance of pharmacist-obtained medication histories using a validated questionnaire. American journal of hospital pharmacy. 1984;41(4):731–2.

46. Gurwich EL. Comparison of medication histories acquired by pharmacists and physicians. American journal of hospital pharmacy. 1983;40(9):1541–2.

47. Ahrens W, Bellach B, Jöckel K. Messung soziogemographischer Merkmale in der Epidemiologie. München: Urban & Vogel; 1998.

48. Hudon C, Fortin M, Soubhi H. Abbreviated guidelines for scoring the Cumulative Illness Rating Scale (CIRS) in family practice J Clin Epidemiol. 2007;60:212.

49. EuroQoL Group: EuroQoL - a new facility for the measurement of health-related quality of life. Health Policy. 1990;16:199-208.

50. Ware J, Kosinski M, Keller S. SF-12: How to Score the SF-12 Physical and Mental Health Summary Scales. The Health Institute. New England Medical Center. Boston: 1995.

51. Gräfe K, Zipfel S, Herzog W, Löwe B. Screening psychischer Störungenmit dem „Gesundheitsfragebogenfür Patienten (PHQ-D)“. Ergebnisse der deutschen Validierungsstudie. Diagnostica. 2004;50(4):171-81.

52. Katz S, Ford A, Moskowitz R, Jackson B, Jaffe M. Studies of Illness in the aged. The index of ADL: A standadized measure of biological and psychological function. JAMA. 1963;185:914-9.

53. Lawton M, Brody E. Assessment of older people: self-maintaining and instrumental activities of daily living. Gerontologist. 1969;9:179-86.

54. Tinetti M. Performance-oriented assessment of morbility problems in elderly patients. J Am Geriatr Soc. 1986;34:119-26.

55. Bös K, Abel T, Woll A, Niemann S, Tittlbach S, Schott N. Der Fragebogen zur Erfassungdes motorischen Funktionsstatus(FFB-Mot). Diagnostica. 2002;48:101-11.

56. Klasen B, Hallner D, Schaub C, Willburger R, Hasenbring M. Validation and reliability of the German version of the Chronic Pain Grade questionnaire in primary care back pain patients. Psycho-Social-Medicine 2004;1:Doc07.

57. Fydrich T, Sommer G, Tydecks S, Brähler E. Social Support Questionnaire (F-SozU): Standardization of short form (K-14). Z Med Psychol. 2009;18:43-8.

Figures

Figure 1: Design of the WestGem-study
This figure shows design and timeframe of the WestGem-study.

Figure 2: CONSORT flowchart of recruitment of practices and patients (projected)
This figure illustrates the projected recruitment flowchart of the WestGem-study.

Tables

Table 1: Components of the intervention and training strategy for its implementation

This table summarizes the components of the intervention and planned training strategies.

Components of intervention and implementation		Content	Participants
Intervention	I. Transfer of medical patient data	Information concerning diagnosis, patient's medication, quality of life, mobility, risk of failing, allergies	GP
	II. Assessment at patients site	Brown bag review and collection of information concerning side effects, adherence, social support and else	Home-care specialist, patient
	III. Anonymised data transfer to pharmacist	Assessment data	Home-care specialist
	IV. Medication review and SOAP note	Assessment of pharmacotherapy, generation of a new medication plan, suggestions for monitoring and patient counseling	2 Pharmacists per patient (one pharmacist generation a first draft of the SOAP note, second one reviews suggested plan)
	V. Transfer of the SOAP note to home-care specialist	SOAP note and advices addressing home-care specialist tasks	Pharmacist
	VI. Information of GP	SOAP note with new medication plan, home-care specialist's note for the GP (concerning for example home-care devices)	Home-care specialist
Training concept for implementation	I. Kick-off meetings	Information concerning organizational aspects, process's time frame, controlling tools, assessment instruments	Home-care specialists, pharmacists, GPs and moderators
	II. profession-specific trainings	Training in medication therapy management, medication review and SOAP-writing<br><br>Training in case management	Home-care specialists, pharmacists, and moderators
	III. training in patient assessment	Assessment instruments, case studies	GPs and moderators
	IV. Process controlling	Ongoing feedbacks on process performance (e.g. accepted interventions, time frame)	All participants

Table 2: Outcome parameters and instruments

This table displays the outcome parameter of the study as well as instruments.

Outcome parameter	Instrument	Data source
**Primary outcome**		
Quality of medication therapy	Medication Appropriateness Index (MAI)	PHARM
**Secondary outcomes**		
Sociodemographic data	Items from German standard questionnaire [47]	CRF, TI
Laboratory data	Patient chart	CRF
Diagnosis	Patient chart (ICD-10)	CRF
Allergies	Patient chart	CRF
Comorbidities	Cumulative Illness Rating Scale Geriatric Version (CIRS) [48]	CRF
Reported side effects	Self-developed item	CRF, TI
Quality of Life	EuroQol (EQ-5D) [49], Short Form 12 Health Questionnaire (SF-12) [50]	PQ, TI
Depression	Patient Health Questionnaire (PHQ9) [51]	PQ
Activities of daily living	ADL, iADL [52, 53]	TI
Risk of falling	Tinetti-Test [54]	CRF
Mobility	FFB-Mot [55]	TI
Cognitive status	Adopted Mini-Mental State Examination (MMSE)	CRF
Pain	German Chronic Pain Scale [56]	PQ, TI
Self-rated health	Self-developed item	PQ
Vision and hearing	Self-developed item	TI
Health behavior (smoking, drinking)	Self-developed item	PQ, TI
Social participation	F-SOZU K14 [57]	TI
Prescribed medication	Patient chart	CRF
Brown bag medication	Self-developed assessment instrument	HCS
Healthcare utilization and costs	Data from inpatient and outpatient care, rehabilitation, medical devices, etc.	CRF
Complexity of medication	Medication regimen complexity index (MRCI) [27]	PHARM
Adherence	MARS, MORISKY [25, 26]	TI
Drug-related Problems	Classification according PCNE version 6.2	PHARM
Inadequate medication	Potentially inadequate medication (German PRISCUS-list) [2]	PHARM
Patients goals of therapy	Self-developed assessment instrument	HCS
Difficulties in medication handling	Self-developed assessment instrument	HCS
Nutrition	Self-developed assessment instrument	HCS
Dizziness	Self-developed assessment instrument	HCS
Mortality	Patient chart	CRF
Accepted medication proposals	Self-developed item	CRF
**Additional parameters**		
Barriers of concept implementation	Qualitative research approach	TI, FG
Practice characteristics	Self-developed questionnaire [39]	TI, on-site monitoring

PHARM = pharmacists, HCS = home-care specialist, PQ = patient questionnaire, CRF = case report form, TI = telephone interview, FG = focus groups

Figure 1

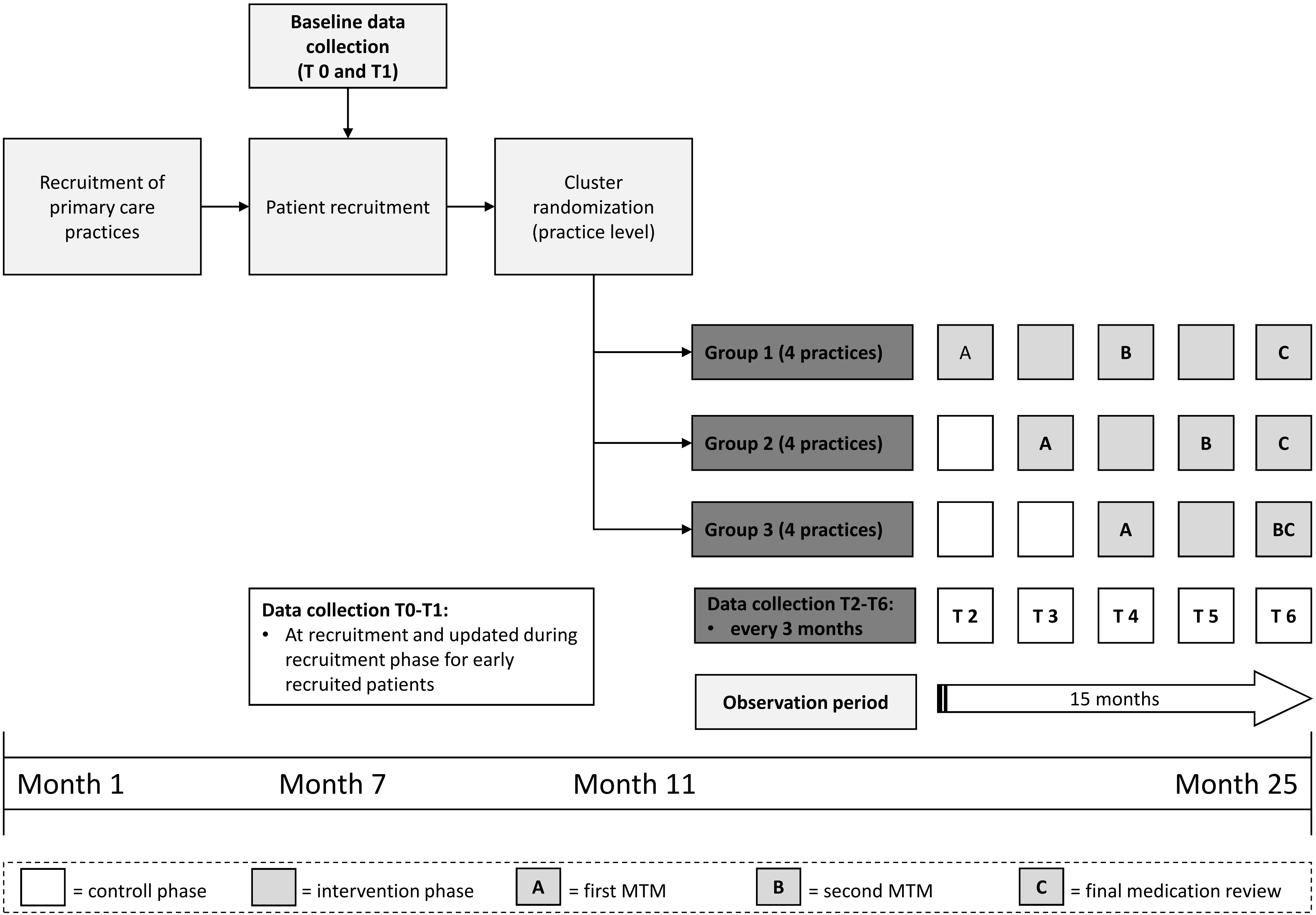
Baseline data collection (T 0 and T1)
Recruitment of primary care practices
Patient recruitment
Cluster randomization (practice level)
Group 1 (4 practices)
A
B
C
Group 2 (4 practices)
A
B
C
Group 3 (4 practices)
A
BC
Data collection T0-T1:
• At recruitment and updated during recruitment phase for early recruited patients
Data collection T2-T6:
• every 3 months
T 2
T 3
T 4
T 5
T 6
Observation period
15 months
Month 1
Month 7
Month 11
Month 25
= controll phase
= intervention phase
A = first MTM
B = second MTM
C = final medication review

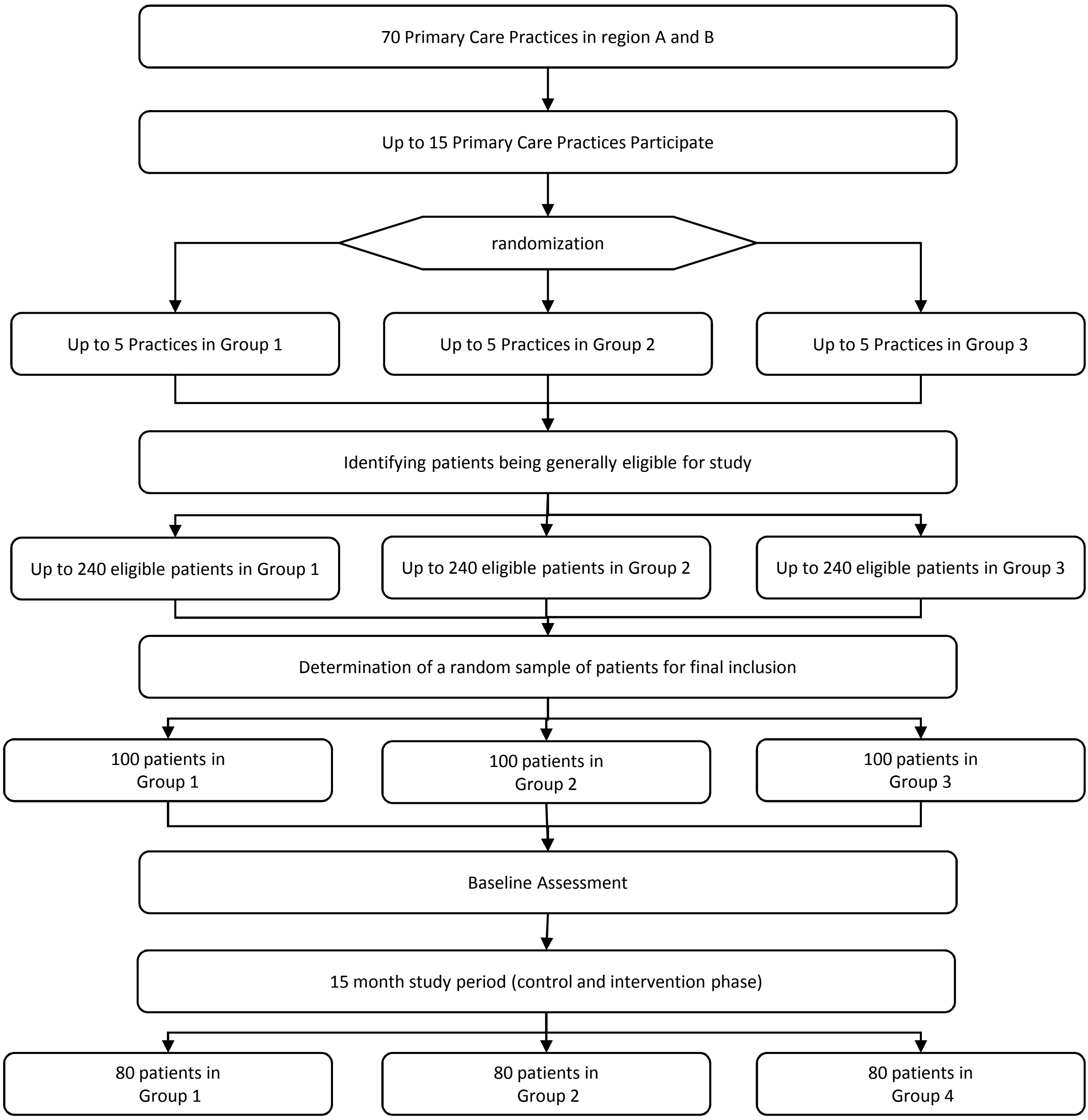
70 Primary Care Practices in region A and B
Up to 15 Primary Care Practices Participate
randomization
Up to 5 Practices in Group 1
Up to 5 Practices in Group 2
Up to 5 Practices in Group 3
Identifying patients being generally eligible for study
Up to 240 eligible patients in Group 1
Up to 240 eligible patients in Group 2
Up to 240 eligible patients in Group 3
Determination of a random sample of patients for final inclusion
100 patients in Group 1
100 patients in Group 2
100 patients in Group 3
Baseline Assessment
15 month study period (control and intervention phase)
80 patients in Group 1
80 patients in Group 2
80 patients in Group 4

Figure 2
