# Supplementary material for: Priority Setting and Influential Factors on Acceptance of Pharmaceutical Recommendations in Collaborative Medication Reviews in an Ambulatory Care Setting – Analysis of a Cluster Randomized Controlled Trial (WestGem-Study)
Source: PLoS One. 2016 Jun 2;11(6):e0156304. doi: 10.1371/journal.pone.0156304 (PMC4890849; doi:10.1371/journal.pone.0156304)
Supplement: S2 Protocol — (PDF) [file pone.0156304.s005.pdf]

WestGem-Studiengruppe vertreten durch:

Bergisches Kompetenzzentrum für Gesundheitsmanagement und Public Health

Bergische Universität Wuppertal

Rainer-Gruenter-Str. 21

42119 Wuppertal

STUDIENPROTOKOLL

Prospektive, cluster-randomisierte, kontrollierte Studie zur Untersuchung der Wirksamkeit und der Kosten eines professions- und organisationsübergreifenden Medikationsmanagements bei multimorbiden Patienten mit Polypharmazie

---

Kurztitel: Medikationsmanagement bei multimorbiden Patienten

Studien-Code: WestGem – GW 2076

08.05.2013

(Version 1.1)

Gefördert durch die Europäische Union und das Ministerium für Gesundheit, Emanzipation, Pflege und Alter des Landes Nordrhein-Westfalen

INHALTSVERZEICHNIS

1 ALLGEMEINE INFORMATIONEN 4
1.1 PROTOKOLLIDENTIFIZIERUNG 4
1.2 VERTRAULICHKEITSHINWEIS 4
1.3 BETEILIGTE PERSONEN, INSTITUTIONEN UND TEILNEHMENDE STUDIENZENTREN 4
1.4 FÖRDERNDE INSTITUTIONEN 7
1.5 ZUSAMMENFASSUNG 7
1.6 SYNOPSIS 9
1.6 FLOW-CHART ZUR STUDIE 11
2 RATIONALE UND FRAGESTELLUNG 13
2.1 AUSGANGSSITUATION 13
2.2 STAND DER FORSCHUNG 16
2.3 BESCHREIBUNG DER INTERVENTION 19
2.3.1 Ausgangssituation 19
2.3.2 Das professions- und organisationsübergreifende Medikationsmanagement 21
2.3.3 Informationsaustausch zwischen den Professionen 23
2.4 ZIELSETZUNG DER STUDIE 25
3 STUDIENDESIGN 27
3.1 STUDIENTYP 27
3.2 STUDIENPOPULATION 27
3.2.1 Einschlusskriterien 27
3.2.2 Ausschlusskriterien 28
3.3 TEILNEHMENDE STUDIENZENTREN 28
3.4 RANDOMISIERUNG 28
3.5 STUDIENABLAUF 29
3.5.1 Patientenrekrutierung 29
3.5.2 Studien- und Dokumentationsverlauf 30
3.5.3 Beobachtungsdauer 33
3.6 ZIELPARAMETER 33
3.6.1 Primäre Zielparameter 33
3.6.2 Sekundäre Zielparameter 34
3.7 BEOBACHTUNGSPARAMETER 35
4 STUDIENORGANISATION 38
4.1 PLATZIERUNG DER STUDIE 38
4.2 BETREUUNG DER TEILNEHMENDEN PRAXEN/MONITORING 38
4.3 NEWSLETTER 38
5 BIOMETRIE 39
5.1 FALLZAHLSCHÄTZUNG 39
5.2 STATISTISCHE AUSWERTUNG 39
5.2.1 Definition von Auswertungskollektiven 39
5.2.2 Beschreibung der Studienpopulation 40
5.2.3 Auswertung 40
5.2.4 Subgruppenanalyse 40
5.2.5 Umgang mit fehlenden Werten 40
5.2.6 Gesundheitsökonomische Evaluation 41
6 DATENMANAGEMENT 42
6.1 PATIENTENIDENTIFIKATIONSLISTE 42

6.2 DATENERHEBUNG 42
6.3 DATENVERARBEITUNG 44
6.4 AUFBEWAHRUNG DER STUDIENUNTERLAGEN 45
6.5 DATENSCHUTZ 45

7 GESETZLICHE GRUNDLAGEN UND ADMINISTRATIVE ASPEKTE 46
7.1 ETHISCHE GRUNDSÄTZE 46
7.1.1 Ethikkommission 46
7.1.2 Aufklärung und Einwilligung der Patienten 46
7.1.3 Verwendung, Speicherung und Weitergabe von Daten 46
7.1.4 Gesetzliche Grundlagen 46
7.2 VERSICHERUNG 46
7.3 UNERWÜNSCHTE EREIGNISSE 47
7.4 FINANZIERUNG 47
7.5 ABSCHLUSSBERICHT UND PUBLIKATION 47
7.6 EINHALTUNG DES PROTOKOLLS UND PROTOKOLLÄNDERUNGEN 47

8 LITERATURVERZEICHNIS 48

9 ANHANG 51

1 ALLGEMEINE INFORMATIONEN

1.1 Protokollidentifizierung

Studien-Code: WestGem – GW 2076

Protokollnummer: 1.1

EudraCT-Nr.: entfällt

Datum der Protokollversion: 06.05.2013

1.2 Vertraulichkeitshinweis

Der Inhalt des Protokolls ist vertraulich zu behandeln und darf ohne Zustimmung der Studienleitung weder mündlich noch schriftlich an Unbeteiligte weitergegeben werden.

1.3 Beteiligte Personen, Institutionen und teilnehmende Studienzentren

Studienleiter und korrespondierender Antragsteller

	Jr.-Prof. Dr. Juliane Köberlein-Neu
**Name des Unternehmens bzw. der Institution**	Bergische Universität Wuppertal<br>Ausführende Stelle:<br>Bergisches Kompetenzzentrum für Gesundheitsmanagement und Public Health
**Straße/Hausnummer**	Rainer-Gruenter-Str. 21
**PLZ/Ort**	42119 Wuppertal
**Telefon**	+49 (0) 202 439 1381<br>+49 (0) 202 439 1384
**E-Mail**	koeberlein@wiwi.uni-wuppertal.de
**Internet**	www.gesundheit.uni-wuppertal.de<br>www.versorgungsforschung.uni-wuppertal.de

Pharmazie

	Olaf Rose, Apotheker, PharmD, e.K.<br>*Isabell Waltering, Apothekerin, PharmD*
**Name des Unternehmens bzw. der Institution**	Elefanten-Apotheke, gegr. 1575
**Straße/Hausnummer**	Steinstr. 14
**PLZ/Ort**	48565 Steinfurt
**Telefon**	+49 (0) 2551 5435

**Telefax**	+49 (0) 2551 6236
**E-Mail**	rose@elefantenapo.de
**Internet**	www.elefantenapo.de

Pflege und Wohnberatung

	Ursula Woltering
	Martin Kamps
	Silke Saalmann
	Manfred Kehr
**Name des Unternehmens bzw. der Institution**	Verein Alter und Soziales e.V.
**Straße/Hausnummer**	Wilhelmstr. 5
**PLZ/Ort**	59227 Ahlen
**Telefon**	+49 (0) 2382 59 467
**Telefax**	+49 (0) 2382 59 730
**E-Mail**	wolteringu@stadt.ahlen.de
**Internet**	www.kaa-ahlen.de

	Heinz Teupen
	Angela Grosse
**Name des Unternehmens bzw. der Institution**	Kreis Steinfurt<br>Ausführende Stellen:<br>Sozialamt – Altenhilfe und Pflegeberatung
**Straße/Hausnummer**	Tecklenburger Str. 10
**PLZ/Ort**	48565 Steinfurt
**Telefon**	+49 (0) 2551 69 2253<br>+49 (0) 2551 69 2363
**Telefax**	+49 (0) 2551 69 12236
**E-Mail**	Heinz.teupen@kreis-steinfurt.de
**Internet**	www.kreis-steinfurt.de

Case Management

	Prof. Dr. Hugo Mennemann
**Straße/Hausnummer**	Lindenbreie 15
**PLZ/Ort**	48161 Münster
**Telefon**	+49 (0)251 390 4569
**Telefax**	--
**E-Mail**	h.mennemann@katho-nrw.de

Biometrie

	Prof. Dr. Walter Lehmacher,<br>*Dr. Lena Herich*
**Name des Unternehmens bzw. der Institution**	Institut für medizinische Statistik, Informatik und Epidemiologie (IMSIE) der Universität zu Köln<br>Leiter: Prof. Dr. Walter Lehmacher
**Straße/Hausnummer**	Kerpener Str. 62
**PLZ/Ort**	50937 Köln
**Telefon**	+49 (0) 221 478 6501
**Telefax**	+49 (0) 221 478 6520
**E-Mail**	walter.lehmacher@uni-koeln.de

Teilnehmenden Studienzentren

Bisher konnten die folgenden Studienzentren für eine Teilnahme am Projekt gewonnen werden:

Modellregion Ahlen

Praxis Detlef Kahl
Alter Hof 7-9
59227 Ahlen

Praxis Dr. med. Hans-Werner Henseleit
Richard-Wagner-Str. 11
59227 Ahlen

Modellregion Steinfurt

Praxis Matthias Wissing
Bahnhofstr. 9
48612 Horstmar

Praxis Ingrid Lummer
Drepsenhoek 4
48565 Steinfurt

Praxis Sabine Heidler
Im Biewing 9
48612 Horstmar

Praxis Dr. med. Reinhard Stahl
Königstr. 8
48612 Horstmar

Praxis Martina Heine
Münsterstr. 24
48565 Borghorst

Praxis Dr. Andreas Maib
Tecklenburger Str. 34
48565 Steinfurt

Praxis Friedrich-Wilhelm Spelsberg
Wippert 8
48565 Steinfurt

1.4 Fördernde Institutionen

Die Förderung der Studie erfolgt durch die Europäische Union und durch das Ministerium für Gesundheit, Emanzipation, Pflege und Alter des Landes Nordrhein-Westfalen im Rahmen des Ziel 2-Wettbewerbs IuK & Gender Med.NRW aus dem Jahr 2011.

1.5 Zusammenfassung

Ziel der Studie ist die modellhafte Erprobung eines durch niedergelassene Ärzte und Ärztinnen (behandelnder Primärarzt bzw. Primärärztin), Apotheker und Apothekerinnen sowie durch die Pflege- und Wohnberatung gemeinsam getragenen, professions- und organisationsübergreifenden Medikationsmanagement zur Verbesserung der Arzneimittelversorgung älterer Menschen mit Multimorbidität. Hiermit möchte das Vorhaben an die Erkenntnisse anderer Forschergruppen anknüpfen und die Arzneimittelversorgung älterer Menschen durch trägerübergreifende Vernetzung optimieren. Ein besonderer Schwerpunkt wird dabei auf die Behandlung kardiovaskulärer Erkrankungen gelegt. In der cluster-randomisierten prospektiven kontrollierten Studie in den zwei Modellregionen Ahlen und Steinfurt werden die Wirksamkeit und die Kosten der interprofessionellen Zusammenarbeit bewertet.

Dazu werden insgesamt 240 multimorbide Patienten mit Polypharmazie über 12 Monate dokumentiert. Die Randomisierung erfolgt auf Ebene der teilnehmenden Hausarztpraxen,

welche im Rahmen eines Stepped Wedge Design („Wartegruppendesign“) sequentiell alle drei Monate in die Konzeptgruppe (Interventionsgruppe) aufgenommen werden

Trotz des randomisierten, kontrollierten Charakters der Studie erfolgt kein direkter Eingriff in den Behandlungs- und Therapieablauf des Patienten. Im Rahmen des professions- und organisationsübergreifenden Medikationsmanagement, welches in der Interventionsgruppe durchgeführt wird, werden dem betreuenden Hausarzt lediglich weiterführende Informationen zum sozialen Umfeld des Patienten, zur Selbstmedikation sowie zu Interaktionseffekten und potentiell inadäquaten Arzneimitteln der eingeschlossenen Patienten zur Verfügung gestellt. Die Entscheidung über den Einbezug dieser Informationen in die therapeutische Entscheidung obliegt dem Behandler.

Primärer Zielparameter der Studie ist der Unterschied zwischen dem mittleren MAI-Score vor und nach dem Wechsel der Patienten in die Konzeptgruppe. Sekundäre Zielparameter sind die Lebensqualität sowie das Kosten-Effektivitäts- und Kosten-Nutzwert-Verhältnis zwischen Interventions- und Kontrollgruppe

1.6 Synopsis

**Titel der Studie**	Prospektive, cluster-randomisierte, kontrollierte Studie zur Untersuchung der Wirksamkeit und der Kosten eines professions- und organisationsübergreifenden Medikationsmanagements bei multimorbiden Patienten mit Polypharmazie
**Kurzbezeichnung der Studie**	Medikationsmanagement bei multimorbiden Patienten
**Indikation**	Multimorbidität
**Primäres Ziel der Studie**	Vergleich der Angemessenheit der Arzneimitteltherapie
**Sekundäres Ziel der Studie**	Lebensqualität<br>Arzneimittel- und Versorgungskosten;<br>Funktionaler Status der Patienten;<br>Lebenslagenorientierung der Versorgung;<br>Zusammenarbeit der Professionen
**Studiendesign**	Cluster-randomisierte, kontrollierte, prospektive und multizentrische Kohorten-Studie
**Studienpopulation**	*Einschlusskriterien:*<br>• Alter ≥ 65 Jahre<br>• Mind. 3 chronische Erkrankungen aus 2 verschiedenen Organsystemen<br>o Organsysteme werden Anhand der Kapitel der ICD-10 Klassifizierung definiert<br>o Für Chronische Erkrankungen gilt eine Liste mit 46 Erkrankungen<br>• Eine der Erkrankungen muss mindestens bereits seit drei Quartalen der letzten 12 Monate bestehen (= Chroniker)<br>• Mind. ein Arztbesuch in jedem der letzten drei Quartale<br>• Mind. ein kardiovaskuläre Erkrankung<br>• fünf und mehr Dauermedikationen (> 3 Monate) mit systemischen Effekten<br>• unterschriebene Teilnahmeerklärung<br>• Fähigkeit, ggf. mit Unterstützung Fragebögen zu beantworten<br><br>*Ausschlusskriterien:*<br>• Erkrankung, die eine Lebenserwartung weniger als 12 Monate bedingt<br>• Teilnehme an einer anderen klinischen Studie innerhalb der letzten 30 Tage
**Patienten- und Zentrenzahl**	Es werden 240 Patienten rekrutiert, welche sich gleichmäßig auf die Modellregionen verteilen sollen. Angestrebt wird die Teilnahme von 12-16 Hausarztpraxen.
**Intervention**	*professions- und organisationsübergreifenden Medikationsmanagement:*<br>Der behandelnde Hausarzt übermittelt an die örtliche Pflege- und Wohnberatung auf Einverständnis des Patienten hin Adress-, Stamm-, Diagnose und Arzneimitteldaten. Diese werden durch die Pflege- und Wohnberatung um Informationen zur Selbstmedikation des Patienten, Sturzgefährdung sowie

	zum häuslich-sozialen Umfeld ergänzt und pseudonymisiert an ein Team aus Pharmazeuten weitergeleitet. Diese prüfen die Medikation des Patienten auf Interaktionen sowie im Hinblick auf die häuslich-soziale Situation des Patienten und geben ggf. Empfehlungen für eine Veränderung der Therapie ab. Die Pflege- und Wohnberatung ergänzt die Empfehlungen der Pharmazeuten um Aspekte aus dem Pflegebereich und leitet alle Vorschläge an den behandelnden Hausarzt weiter. Dieser kann nun die ihm vorliegenden Informationen im Rahmen seiner Patientenbehandlung nutzen.
**Primäre Studienendpunkte**	• Veränderung des Medication Appropriateness Index (MAI) Scores durch den Wechsel in die Konzeptgruppe
**Sekundärer Studienendpunkt**	• Lebensqualität;<br>• Kosten-Effektivitäts-Verhältnis;<br>• Kosten-Nutzwert-Verhältnis<br>• Komplexität der Arzneimitteltherapie (Medication Regimen Complexity Index (MRCI), Anzahl der Arzneimittel, Anzahl der Einzeldosen pro Tag)<br>• Berichtete Therapieadhärenz (Morisky und MARS)<br>• Belastung durch die Therapie<br>• Anzahl berichteter (potentieller) UAWs<br>• Funktionaler Status<br>• Sturzgefahr<br>• Selbstberichtete Gesundheit<br>• Medikationskosten<br>• Krankenhaustage<br>• Krankheitskosten aus der Perspektive des Hausarztes<br>• Qualität der Versorgung vulnerabler Patientengruppen (ACOVE)
**Biometrie**	• Die Auswertung der primären Zielgröße erfolgt wird mit Hilfe eines Gemischten Modells mit den festen Faktoren Studiengruppe und Zeit, sowie zufälligen Effekten zur Berücksichtigung der Clusterstruktur und der wiederholten Messungen innerhalb eines Individuums,<br>• Die sekundären Zielgrößen werden analog ausgewertet.
**Zeitplan**	*Patientenbezogen:* Beobachtungs-/Dokumentationsdauer pro Patient: 6 Monate retrospektiv; 12 Monate prospektiv<br>*Studienbezogen:* 4-6 Monate Rekrutierungszeitraum; 12 Monate Studienzeitraum

1.6 Flow-Chart zur Studie

	t 0	t 1	t 2	t 3	t 4	t 5
Patienteneinverständniserklärung und Datenschutz	A					
Überprüfung der Ein- und Ausschlusskriterien	A					
Dokumentation der Soziodemographische Merkmale						
Geburtsmonat/Jahr, Geschlecht	A					
Familienstand, Haushaltsgröße, Wohnumstände		T	T	T	T	T
Bildungsabschluss, ehemaliger Beruf, Migrantenstatus		T				
Versichertenstatus, Krankenkasse, Wahltarife	A	T	T	T	T	T
Einkommen (Einkommensarten, etwaige Höhe)		T				T
Erfassung der Morbidität auf Basis der Patientenakte						
Größe, Gewicht	A	A	A	A	A	A
Vitalparameter und Laborwerte (nach Verfügbarkeit)	A	A	A	A	A	A
Laborwerte (nach Verfügbarkeit)	A	A	A	A	A	A
Aktuelle Akut- und Dauerdiagnosen	A	A	A	A	A	A
Kardiovaskuläres Risiko	G	G	G	G	G	G
Allergien und Unverträglichkeiten	A					
Komplexität der Morbiditätsstruktur	A	A	A	A	A	A
Symptome potentieller unerwünschter Arzneimittelwirkungen	A	A	A	A	A	A
Akutmedikation: Wirkstoff, Wirkstärke, Dosis etc.	A	A	A	A	A	A
Dauermedikation: Wirkstoff, Wirkstärke, Dosis etc.	A	A	A	A	A	A
Nicht-Medikamentöse Therapien	A	A	A	A	A	A
Erfassung zusätzlicher Informationen zur Morbidität						
Lebensqualität	P	PT	PT	PT	PT	PT
Depression	P	PT	PT	PT	PT	PT
Pflegestufe	A	T	T	T	T	T
Erfassung zusätzlicher Informationen zum funktionalen Status						
Einschränkungen bei Aktivitäten des täglichen Lebens		T	T	T	T	T
Sturzgefahr	A	A		A		A

Mobilität		T	T	T	T	T
Kognitiver Status	A			A		A
Schmerz	P	T	T	T	T	T
Selbstbewertete Gesundheit	P	T	T	T	T	T
Sehen und Hören		T	T	T	T	T
Erfassung des Gesundheitsverhaltens und der sozialen Unterstützung						
Selbstmanagement		T		T		T
Rauchverhalten und Alkoholkonsum	P	T				T
Soziale Einbindung und Unterstützung		T		T		T
Erfassung zusätzlicher Informationen zur Arzneimitteltherapie						
Komplexität der Arzneimitteltherapie		Ph	Ph	Ph	Ph	Ph
Potentiell inadäquate Medikation (PIM)		Ph	Ph	Ph	Ph	Ph
Berichtete Therapieadhärenz		T	T	T	T	T
Arzneimittelbezogene Probleme		Ph	Ph	Ph	Ph	Ph
Belastung durch die derzeitige Arzneimitteltherapie		T	T	T	T	T
Bewertung des Inanspruchnahmeverhaltens						
Anzahl der Haus- und Facharztbesuche	A	AT	AT	AT	AT	AT
Anzahl Krankenhausaufenthalte, Dauer und Ursache	A	AT	AT	AT	AT	AT
weitere Inanspruchnahme von Gesundheitsleistungen	A	AT	AT	AT	AT	AT
Leistungen im Rahmen des SGB XI		T	T	T	T	T
Dokumentation der Versorgungsstruktur						
Praxisorganisation (z.B. Einzel- oder Gemeinschaftspraxis)	A					
Qualität der Versorgung vulnerabler Patientengruppen	A					A
Erwartungen an das Medikationsmanagement	Pr					
Bewertung der Zusammenarbeit				Pr		Pr
Abschlussdokumentation						
Dokumentation bei Studienabbruch						A

T_0: Baseline; T_1: Ende der Rekrutierungszeit; T_2: Ende Rekrutierungszeit + 3 Monate; T_3: Ende Rekrutierungszeit + 6 Monate; T_4: Ende Rekrutierungszeit + 9 Monate; T_5: Ende Rekrutierungszeit + 12 Monate/Studienende;
A = durch den Arzt, P = durch den Patient, T = im Telefoninterview; Ph = durch Pharmazeut; G = aus anderen Beobachtungsparametern generierbar; Pr = alle beteiligten Professionen

2 RATIONALE UND FRAGESTELLUNG

2.1 Ausgangssituation

Es ist hinlänglich bekannt, dass Auftreten und Komplexität von Multimorbidität mit zunehmendem Alter steigen. Obwohl bislang keine klare Definition des Konzepts der „Multimorbidität“ hinsichtlich Art, Anzahl und Schwere der zugrundeliegenden Erkrankungen existiert [1], besteht kein Zweifel daran, dass das gleichzeitige Vorliegen mehrerer Erkrankungen eine hohe sozialmedizinische und gesundheitsökonomische Bedeutung hat und die Gesellschaft vor sozioökonomische, medizinische und pflegerische Herausforderungen stellt [2]. Internationale Studien belegen eine hohe Prävalenz von Multimorbidität speziell innerhalb der älteren Bevölkerung. So stellten beispielsweise Fortin et al. im Rahmen einer Literaturübersicht Multimorbiditätsraten zwischen 49% bei Patienten und Patientinnen unter 45 Jahren und 99% bei Betroffenen über 65 Jahren fest [3]. In Deutschland gibt es bislang nur wenige bevölkerungsbezogene Aussagen zur Verbreitung von Multimorbidität [4]. Jüngst wurde hierzu eine Studie von van den Bussche et al. auf Basis von Krankenkassendaten von über 120.000 GKV-Versicherten über 65 Jahre veröffentlicht [5]. Rund 62% der Versicherten wiesen mehr als drei chronische Krankheiten auf, wobei signifikant mehr Frauen zur Gruppe der Multimorbiden zählten. Unter den in der Studie identifizierten sechs häufigsten chronischen Erkrankungen fanden sich mit der Hypertonie und der chronisch ischämischen Herzkrankheit zwei Krankheitsbilder aus dem Indikationsgebiet der kardiovaskulären Erkrankungen. Dieses umfasst eine Reihe epidemiologisch und volkswirtschaftlich bedeutsamer Krankheitsbilder, die Herz und Gefäße betreffen und durch einen multifaktoriellen sowie komplexen Charakter gekennzeichnet sind. Typischerweise zählen dazu Herzinsuffizienz, Myokardinfarkt, Angina Pectoris, Herzarrhythmien, Schlaganfall und die periphere arterielle Verschlusskrankheit [6].

Patienten und Patientinnen mit Herz-Kreislauf-Erkrankungen werden sowohl ambulant als auch stationär behandelt. Über 50% des Patientenkollektivs hausärztlicher Internisten und Internistinnen weisen mindestens eine Diagnose aus dem ICD-10 Kapitel für Krankheiten des Kreislaufsystems (ICD: Internationale Klassifikation der Krankheiten) auf [7]. Über alle Arztgruppen hinweg werden deutschlandweit binnen eines Quartals etwa 18 Millionen Patienten und Patientinnen ambulant wegen Kreislaufkrankheiten behandelt und verursachen dabei Kosten in Höhe von 4 bis 4,6 Milliarden Euro pro Jahr [7]. Wie die vom Statistischen Bundesamt durchgeführte Evaluation der stationären Diagnosedaten des Jahres 2008 belegt, sind es ebenfalls Kreislauferkrankungen, die zu der Mehrzahl der stationären Aufnahmen führen; mit rund 15% aller stationären Fälle (2,7 Mio. Pat.; 1,4 Mio. männlich/1,3 Mio. weiblich) handelt es sich um die größte Diagnosegruppe [8]. Im Ranking

der häufigsten stationären Einzeldiagnosen 2008 gemäß ICD-10 war die Herzinsuffizienz die zweithäufigste Einzeldiagnose, Angina Pectoris folgte auf Platz 4, Hirninfarkt auf dem sechsten Platz, Vorhofflimmern auf Platz 7 und schließlich der akute Myokardinfarkt auf dem achten Rang [8]. Die Todesursachenstatistik 2009 führte die chronisch ischämische Herzkrankheit (40.155 weiblich/33.744 männlich) vor dem akuten Myokardinfarkt (25.292 weiblich/30.934 männlich) und der Herzinsuffizienz (33.088 weiblich/15.883 männlich) an [9]. Bei derartig hohen Morbiditäts- und Mortalitätsraten wundert es nicht, dass im Jahr 2006 von den insgesamt entstandenen Krankheitskosten in Höhe von 236 Milliarden Euro rund 35,2 Milliarden Euro bzw. 14,9%, auf die Gruppe der Krankheiten des Kreislaufsystems entfielen [10].

Die primäre Behandlungsoption kardiovaskulärer Erkrankungen ist die Arzneimitteltherapie [7]. Dieser kommt im Rahmen der Gesamtversorgung der Betroffenen eine wesentliche Bedeutung zu, da ein auf diese Weise stabilisierter Gesundheitszustand des Patienten sowie der Patientin in der Regel die Voraussetzung für die Wahrnehmung übriger Versorgungsangebote wie Physiotherapie bildet. Die evidenzbasierte Arzneimitteltherapie erfolgt unter Rückgriff auf Angiotensininhibitoren (ACE-Hemmer und Angiotensin-Rezeptor-Blocker), die im Jahr 2008 mit 46 Millionen Verordnungen und 6,2 Milliarden täglicher Tagesdosen die Liste der verordnungsstärksten Arzneimittelgruppen anführten [11]. Es ist davon auszugehen, dass multimorbide Patienten und Patientinnen mit Herz-Kreislauf-Erkrankungen täglich durchschnittlich sechs verschiedene Medikamente einnehmen, wobei die Anzahl der Präparate bei über 65-jährigen signifikant höher liegt als in der jüngeren Altersgruppe unter 65 Jahren [12]. Die gleichzeitige Behandlung eines Patienten oder einer Patientin mit mehreren Wirkstoffen, auch Polypharmazie genannt, betrifft insgesamt häufiger Frauen, wobei diese zudem durchschnittlich mehr Arzneimittel einnehmen als Männer [13]. Gerade im Bereich der kardiovaskulären Erkrankungen ist die Verordnung unterschiedlicher Wirkstoffe per se erforderlich und erklärt die bei ca. 40% liegende Prävalenzrate der Polypharmazie (inkl. Over-the-counter-Präparate) bei über 75-jährigen [14]. Doch Multimedikation kann zu Problemen wie unerwünschten Arzneimittelinteraktionen und verminderter Compliance führen [15]. Unerwünschte Arzneimittelereignisse (UAE), die bei ca. 13% der medikamentös ambulant behandelten Patienten und Patientinnen in Verbindung mit Verordnung, Anwendung, Distribution oder Applikation eines Präparats auftreten, verursachen eine erhebliche ökonomische Belastung [16], die Schätzungen zufolge für Deutschland bei über 800 Millionen Euro pro Jahr liegt [17]. Bedingt durch assoziierte Multimorbidität sind in besonderem Maße ältere Patienten und Patientinnen von UAE betroffen [18], darüber hinaus sind Frauen einem erhöhten Risiko ausgesetzt [19]. Die Medikation zur Behandlung kardiovaskulärer Erkrankungen ist im ambulanten Setting für den größten Teil der unerwünschten Arzneimittelereignisse verantwortlich [12, 20, 21].

Zusammenfassend sind also ältere Patienten und Patientinnen sowohl von Multimorbidität als auch von der damit einhergehenden Polypharmazie und schließlich resultierenden unerwünschten Arzneimittelereignissen in hohem Maße betroffen.

Ein erster Schritt zur Vermeidung ungeeigneter Arzneimittelverordnungen in dieser speziellen Risikogruppe wurde in Deutschland durch den vom Bundesministerium für Bildung und Forschung geförderten Forschungsverbund PRISCUS getan. Im Rahmen eines Teilprojekts wurden 83 für ältere Patienten und Patientinnen potenziell inadäquate Medikamente identifiziert und auf der „PRISCUS-Liste“ zusammengefasst [22].

An diese Forschungsaktivitäten möchte die Studie anknüpfen und zielt darauf ab, die Arzneimittelversorgung älterer Menschen mit Multimorbidität durch trägerübergreifende Vernetzung zu optimieren. Ein besonderer Schwerpunkt wird dabei auf die Behandlung kardiovaskulärer Erkrankungen gelegt. Zur Steigerung der Effektivität, Sicherheit und Effizienz der Arzneimittelversorgung wird in Anlehnung an das in anderen Ländern eingesetzte Medikationsmanagement (Medication Therapy Management (MTM)) ein Konzept zur integrierten Zusammenarbeit von niedergelassenen Ärzten und Ärztinnen (behandelnder Primärarzt bzw. Primärärztin), Apothekern und Apothekerinnen sowie Pflege- und Wohnberatern und Pflege- und Wohnberaterinnen evaluiert.

Beim MTM handelt es sich um einen Prozess, der die Optimierung individueller therapeutischer Ergebnisse für Patienten und Patientinnen zum Ziel hat und als Teil einer Medikamentenversorgung aber auch unabhängig von der Abgabe eines Arzneimittels durchgeführt werden kann. Das MTM grenzt sich von den derzeit in deutschen öffentlichen Apotheken angebotenen Beratungsleistungen ab, da nicht nur die Überprüfung und Kommunikation von medikamentenbezogenen Problemen im Fokus stehen. Vielmehr findet in enger Zusammenarbeit mit dem behandelnden Arzt oder der Ärztin die Überarbeitung der medikamentösen Therapie unter Hinzunahme sämtlicher Patienteninformationen, einschließlich Diagnosen und Labordaten statt. Der auf MTM spezialisierte Apotheker bzw. die Apothekerin wird insbesondere untersuchen, ob die verordneten Präparate beim jeweiligen Patienten bzw. bei der entsprechenden Patientin die optimale Medikation darstellen. Darüber hinaus wird geprüft, ob die Dosierung zutreffend ist, Medikamente im Therapieplan fehlen, oder in wie weit Arzneimittel überflüssig sind. Abschließend spielt der Apotheker oder die Apothekerin die gewonnen Erkenntnisse und Informationen in Form eines individuellen Empfehlungsplans an den Arzt bzw. die Ärztin zurück. Die Entscheidung darüber, ob und welche Änderungsvorschläge in der Patientenbehandlung umgesetzt werden, verbleibt beim Arzt oder der Ärztin.

2.2 Stand der Forschung

Die potentiellen Erfolge des Medikationsmanagements lassen sich vor allem an dessen Einsatz in den USA ablesen, wo der Prescription Drug, Improvement and Modernization Act (MMA) seit 2003 die Anwendung eines Medication Therapy Managements bei kostenintensiven Patienten oder Patientinnen vorschreibt. Das MTM wird dort überwiegend vom Pharmazeuten und Pharmazeutinnen in Rücksprache mit dem behandelnden Arzt oder der Ärztin betrieben. Es kann jedoch auch als Beratungsleistung des Apothekers oder der Apothekerin im direkten Kontakt mit dem Patienten/der Patientin angesiedelt sein.

Folgende Studien evaluieren klinische, patientenrelevante und ökonomische Effekte des Medication Therapy Managements.

Die Forschergruppe um Barnett untersuchte retrospektiv über 76.000 MTM-bezogene Abrechnungsdatensätze von nahezu 24.000 Patienten und Patientinnen in den USA, die innerhalb eines Zeitraums von sieben Jahren erfasst wurden. Die zentralen Ergebnisse belegen eine signifikante Verschiebung des Leistungsspektrums von MTM-Pharmazeuten und -Pharmazeutinnen. Stand zu Beginn des Beobachtungszeitraums im Jahr 2000 noch die Bereitstellung von Informationsmaterial im Kontext akuter Erkrankungen für eher junge Patienten und Patientinnen (mittleres Alter 30,4 Jahre) im Vordergrund, wandelte sich das Interventionsspektrum bis zum Jahr 2006 hin zur Betreuung älterer Patienten und Patientinnen (mittleres Alter 57,6 Jahre) mit chronischen Erkrankungen. Der Anteil von direkten Kontakten mit Patienten oder Patientinnen und verschreibenden Ärzten/Ärztinnen nahm dabei signifikant zu. Zudem wurde von den MTM-Pharmazeuten/-Pharmazeutinnen die Kenngröße „erwartete Kostenvermeidung“ dokumentiert. Hier schätzen die Pharmazeuten und Pharmazeutinnen ein, ob die Intervention beispielsweise zur Steigerung der Lebensqualität beitrug, einem Arztbesuch oder einer stationären Einweisung vorbeugte. Die angenommenen vermiedenen Ereignisse wurden mit pauschalen Geldsummen bewertet und auf jeden Abrechnungsfall umgelegt. Hier ergaben sich für das Jahr 2006 erwartete vermiedene Kosten in Höhe von 429 US-$ pro Abrechnungsfall [23].

Eine prospektive Studie von Isetts et al. in Minnesota in den Jahren 2001 und 2002 konnte positive klinische sowie ökonomische Effekte des Medication Therapy Managements belegen. Verglichen wurden 285 Patienten und Patientinnen der Interventionsgruppe, die eine persönliche MTM-Beratung erhielten, mit 252 Patienten und Patientinnen einer historischen Kontrollgruppe. Im Bereich der klinischen Outcomeparameter wurden beispielsweise der Erreichungsgrad der individuellen Therapieziele, dieser konnte im Durchschnitt zwischen dem ersten und letzten MTM-Besuch um 14% gesteigert werden, sowie die Anzahl der identifizierten und gelösten arzneimittelbezogenen Probleme erhoben. Die MTM-Pharmazeuten/-Pharmazeutinnen konnten bei den Patienten und Patientinnen der

Interventionsgruppe 637 Probleme in der Arzneimitteltherapie feststellen, die sowohl Unter-, Über- als auch Fehlversorgung betrafen. Die gesamten jährlichen Gesundheitsausgaben pro Person, die auf Basis einer Stichprobengröße von 186 Personen erhoben wurden, verringerten sich vom Jahr vor der MTM-Intervention bis zum Ende der Intervention um 31,5%. Es wurde ein Return of Investment (ROI) der MTM-Intervention in Höhe von 12,15 US-$ errechnet [24].

Oliveira, Brummel und Miller evaluierten retrospektiv die elektronischen Patientenakten von über 9.000 Personen, die zwischen 1998 und 2008 in Minnesota (USA) am MTM-Programm des Fairview Health Services-Netzwerk teilnahmen. Insgesamt wurden etwa 33.700 Pharmazeuten-Kontakte dokumentiert, bei denen insgesamt über 38.000 arzneimitteltherapiebezogene Probleme identifiziert werden konnten. Bei Einschreibung in das MTM-Programm wurden jene knapp 13.000 Gesundheitszustände erfasst, die nicht dem angestrebten Behandlungsziel entsprachen. Im Zuge der Programmteilnahme verbesserten sich davon 55%. Die von den Pharmazeuten und Pharmazeutinnen geschätzten MTM-bedingten Ersparnisse für das Netzwerk betrugen 86,45 US-$. Den durchschnittlichen Kosten jeder MTM-Intervention von 67,00 US-$ gegenübergestellt, ergibt sich so ein ROI von 1,29 US-$ [25].

Auch Smith und Kollegen konnten bei der Evaluation des in North Carolina erprobten „Senior PHARMAssist program“ positive Effekte des MTM-Ansatzes finden. Anhand der Stichprobe von 506 älteren und finanziell schwachen Teilnehmern und Teilnehmerinnen konnte nachgewiesen werden, dass sich sowohl das Inanspruchnahmeverhalten medizinischer Einrichtungen als auch der allgemeine Gesundheitsstatus über den Beobachtungszeitraum von 24 Monaten signifikant verbesserten [26].

Ebenfalls retrospektiv evaluierte die Forschergruppe um Doucette unter anderem die Kooperationsbereitschaft der verschreibenden Ärzte und Ärztinnen im Rahmen eines Medication Therapy Management Programms, das den Medicaid-Empfängerkreis in Iowa adressierte. Es konnten 150 Patienten und Patientinnen eingeschlossen werden, die in den ersten beiden Jahren nach Gründung am pharmazeutischen Case-Managementprogramm teilnahmen. Die MTM-Pharmazeuten/-Pharmazeutinnen konnten nahezu 900 arzneimittelbezogene Probleme feststellen und sprachen daraufhin 659 Empfehlungen zur Anpassung der Arzneimitteltherapie aus. Insgesamt 47,7% dieser Empfehlungen wurden von den verschreibenden Ärzten und Ärztinnen akzeptiert und in geänderte Therapiepläne umgesetzt. Dabei wurde die Empfehlung, eine Medikation abzubrechen, am häufigsten befolgt, der Rat, eine neue Medikation zu beginnen, fand die geringste Zustimmung [27].

Im Rahmen einer Pilotstudie mit einem Vorher-Nachher-Design und zwei Kontrollgruppen untersuchten Christensen und Kollegen ein MTM-Angebot in North Carolina. Bei den 67

Teilnehmern und Teilnehmerinnen der Interventionsgruppe konnten MTM-Pharmazeuten/-Pharmazeutinnen durchschnittlich je 3,6 Probleme in der Arzneimitteltherapie identifizieren, wobei potenzielle Unterversorgung die häufigste Diagnose war. Insgesamt fand in rund 50% der Fälle durch den verschreibenden Arzt oder die verordnende Ärztin eine Anpassung der Medikation statt. In Fällen, in denen vom Pharmazeuten und Pharmazeutinnen eine anzunehmende Überversorgung festgestellt wurde, folgten die Ärzte und Ärztinnen den Empfehlungen besonders häufig. Hinsichtlich der Gesamtzahl der verordneten verschreibungspflichtigen Arzneimittel und der hierfür vom Versicherer geleisteten Ausgaben konnte zwar eine negative Tendenz, hin zu einer kostengünstigeren Versorgung, festgestellt werden, aufgrund der kleinen Interventionsgruppengröße blieben diese Ergebnisse jedoch nicht signifikant [28].

Die vorgestellten Studien konnten belegen, dass die Anwendung des MTM-Konzepts Medikationsprobleme wie Über-, Unter- oder Fehlversorgung identifiziert, sich die teilnehmenden Patienten und Patientinnen ihren Therapiezielen nähern, die Anzahl der insgesamt verordneten Arzneimittel reduziert werden kann und somit verminderte Ressourcenverbräuche auf Seiten der Patienten und Patientinnen sowie der Versicherer erzielt werden können. Der finanzielle Mehraufwand, der durch Implementierung des MTM-Ansatzes entstand, wurde in den Studien, die eine Evaluation der ökonomischen Outcomeparameter vornahmen, durch reduzierten Gebrauch verschreibungspflichtiger Arzneimittel, vermiedene Interaktionseffekte und verhinderte stationäre Aufnahmen vollständig kompensiert.

Allerdings können ausländische Konzepte und die mit ihnen erzielten Effekte nicht ohne weiteres auf Deutschland übertragen werden, da sich die Rahmenbedingungen des Gesundheitssystems, etwa institutionelle und strukturelle Verknüpfungen, aber auch ökonomische Anreizsysteme und kulturelle Hintergründe der Patienten und Patientinnen sowie die Ärzte- und Apothekermentalität erheblich unterscheiden. Diese Argumentation bestärken unter anderem Arbeiten von Weingarten et al. 2002 [29] und Wensing et al. 2006 [30], die eine Abhängigkeit der Effektivität neuer Konzepte von der Art ihrer Implementierung darlegen.

In Deutschland finden Ansätze des Medikationsmanagements derzeit in verschiedenen Formen modellhaft Anwendung. So wurde mit Hilfe eines Arzneimittelsicherheitssystems in Erlangen versucht, computergestützt die Medikation im stationären Sektor zu optimieren [31]. Gemeinsam mit der Bundesvereinigung Deutscher Apothekerverbände untersuchte eine Forschergruppe aus Krefeld in Anlehnung an das amerikanische System, in wie weit sich durch die Begutachtung des Medikationsplans durch den Arzt/die Ärztin und den Pharmazeut/die Pharmazeutin die Arzneimitteltherapie im ambulanten Bereich optimieren

lässt. Hierzu selektierten der Pharmazeut und die Pharmazeutin relevante Patienten und Patientinnen, suchten diese im häuslichen Umfeld auf und analysierten gemeinsam mit einem unabhängigen Hausarzt bzw. Hausärztin die Medikationspläne. Der Ansatz stellte sich in der Studie als wirksam, aber sehr kostenintensiv dar [32]. Kruse et al. erörterten das Potential des Medication Therapy Managements im Alten- und Pflegeheim [33].

Die erfolgreiche Beteiligung von Pflege- und Wohnberatungen an der Versorgung älterer Menschen zeigt ein Projekt in Ahlen, das im Rahmen des Modellprogramms zur Weiterentwicklung der Pflegeversicherung gemäß §8 Abs. 3 SGB XI durchgeführt wurde. Die Pflege- und Wohnberatung erfasste anhand eines Assessmenttools die komplexe Hilfesituation der Patienten und Patientinnen und leitete daraufhin den Maßnahmenkatalog ab. Die im Projekt erstellte Kosten-Nutzen-Analyse ergab bei beratenen Ahlenern und Ahlenerinnen im Vergleich zu den nichtberatenen Ahlenern und Ahlenerinnen eine monatliche Kosteneinsparung für Leistungen nach SGB XI von 200 € pro Beratenem [34].

2.3 Beschreibung der Intervention

2.3.1 Ausgangssituation

Ziel der Studie ist die patientenorientierte Zusammenarbeit niedergelassener Ärzte, Pharmazeuten sowie Pflege- und Wohnberater. Gemäß Rollenzuschreibung erfüllen diese in der Regelversorgung bereits gesetzlich festgesetzte Aufgaben, welche im Rahmen der Intervention um eine gemeinsame Kommunikationsstruktur und den Informationsaustausch erweitert werden sollen. Die Intervention greift weder direkt noch indirekt in die Regelversorgung des Patienten nach SGB V sowie in die ärztliche Behandlung ein. Dem behandelnden Hausarzt wird durch die im Rahmen der interprofessionellen Zusammenarbeit gewonnen Informationen lediglich eine erweiterte Entscheidungsgrundlage zur Verfügung gestellt.

Folgende Tabelle veranschaulicht die formal zugestandenen und die fachlich nach Selbstanspruch qua Professionen und Disziplin beanspruchten Kompetenzen.

Tabelle 1: Formale und fachliche Kompetenzen der Professionen

Profession	formale Kompetenz	fachliche Kompetenz
Arzt/Ärztin	Diagnostik und Therapie gemäß SGB V	Therapiesteuerung; Vernetzungs-arbeit; psychosoziale Betreuung
Klinische Pharmazeuten/ Pharmazeutinnen, Apotheker/Apothekerin		aktives Medikationsmanagement; Unterstützung bei Diagnostik und Therapie
Pflege- und Wohnberater/	Pflege- und Wohnberatung	Information, Beratung; Case

Pflege- und Wohnberaterin	gemäß SGB XI	Management; Wohnungs-anpassung; Vernetzungsarbeit

Dem approbierten, niedergelassenen **Arzt** ist nach SGB V die medizinische Kompetenz in Diagnostik und Therapie mit Verschreibungsrecht zugeschrieben. Der niedergelassene Arzt ist in der Regel die Vertrauensperson, deren Empfehlungen die Patienten und Patientinnen folgen. Er steuert die Situation mit Blick auf die medizinischen Therapievorschläge und Verschreibungen notwendiger Maßnahmen, die zum Teil von Dritten ausgeführt werden.

Pharmazeuten treten in der Studie nicht als Apotheker, sondern als fachliche Spezialisten mit spezifischem pharmakologischem Wissen auf: mit Blick auf medizinisch messbare Parameter im Einzelfall erarbeiten sie ein Medikationskonzept, das dem Bedarf des Patienten und der Patientin optimal entspricht und unerwünschte Wechselwirkungen zwischen den Medikamenten vermeidet. Sie begleiten den Patienten und die Patientin angesichts der Wirkung der Medikamente unter Berücksichtigung des allgemeinen Gesundheitszustandes und des spezifischen gesundheitlichen Bedarfs. Sie stehen dem Arzt diagnostisch sowie therapeutisch unterstützend zur Seite und geben eine Empfehlung ab, welcher der Arzt folgen kann. In formaler Hinsicht kommt ihnen in der Zusammenarbeit mit den Ärzten sowie den Pflege- und Wohnberatern keine gesetzlich fixierte Kompetenz zu.

Pharmazeuten sind in diesem Projekt ohne direkten Kontakt zum Patienten auf der Grundlage der weitergeleiteten Daten des Arztes und der Pflege- und Wohnberatung beratend tätig.

Pflege- und Wohnberater sind inzwischen in der Regel in Pflegestützpunkten nach § 92c SGB XI mit den Aufgaben Information, Auskunft und Beratung zu Pflegefragen und Care Management oder nach § 7a SGB XI als Case Manager oder Case Managerinnen bei den Pflegekassen tätig. Wurden keine Stützpunkte eingerichtet, sind die Berater und Beraterinnen oftmals nach § 4 Landespflegegesetz NRW bei den Kommunen angesiedelt.

Ihre Aufgabe ist es aber auch, in komplexen Problemlagen, in denen mehrere Leistungserbringer und Kostenträger involviert sind, bei Bedarf den Einzelfall nach dem Handlungskonzept Case Management zu steuern: sie nehmen die Situation im Assessment mit Blick auf die gesundheitliche, soziale, räumliche sowie psychische Situation auf und betrachten das soziale System inklusive der primären Hilfen durch Angehörige und die Nachbarschaft. Auch Wohnumfeldverbesserungen kommen in den Blick. Die Pflege- und Wohnberater stellen mit den Betroffenen einen Hilfeplan auf und vernetzen die am Einzelfall beteiligten Dienste – Ergotherapeuten und Ergotherapeutinnen, Physiotherapeuten und Physiotherapeutinnen, Pflegedienste, Ärzte usw. – und behalten die Fallführung. Das Case

Management erfolgt stets vor dem Hintergrund der im SGB XI vorgesehenen Regelversorgung.

2.3.2 Das professions- und organisationsübergreifende Medikationsmanagement

Das vorgesehene professions- und organisationsübergreifende Medikationsmanagement umfasst Prozesse des international eingesetzten Medication Therapy Managements (MTM) sowie Prozeduren des Fallmanagements der Pflege- und Wohnberatung und die medizinische Expertise des behandelnden Arztes/der behandelnden Ärztin.

Nach Einstufung eines Patienten oder einer Patientin als geeignet für die Studie (Einschlusskriterien siehe Kapitel 3.2) und Zustimmung des Patienten zur Einverständniserklärung werden vom Studienarzt die für die Patientenbewertung notwendigen Daten an die in den Modellregionen zuständigen Pflege- und Wohnberatungen übermittelt. Bei diesen Daten handelt es sich um Patientenstammdaten, die aktuelle Medikation, Größe, Gewicht, Alter und Geschlecht, Laborwerte (falls verfügbar), Diagnosen, die Medikationsgeschichte und bereits bekannte Problemlagen des Patienten. Eine Abfrage der Lebensqualität mit Hilfe eines standardisierten Fragebogens (SF-12) ist ebenfalls eingeschlossen.

Die Pflege- und Wohnberatung setzt sich anschließend mit dem zu beratenden Patienten in Verbindung und vereinbart einen Assessment-Termin im häuslichen Umfeld. Jeder persönliche Kontakt beginnt mit einem Assessment der sozialen Situation, der Therapie-Compliance und Umsetzbarkeit der medizinischen Behandlung (Gesundheitskompetenz), der Wohnsituation sowie der vorliegenden Kompetenzen bzgl. Grundpflege, Haushalt, Ämtern, Behörden und Aufrechterhaltung sozialer Beziehungen. Aufbauend auf diese vorgenommene Bewertung entscheidet die Pflege- und Wohnberatung über den weiteren Verlauf der Betreuung:

1. Beendigung, da kein weiterer Handlungsbedarf besteht;
2. Beratung mit Vorschlägen zu einer Hilfeplanung, die der Patient oder die Patientin selbst oder seine Bezugspersonen umsetzen. Hier erfolgt im Anschluss ein Folge-Assessment, um den Erfüllungsgrad der Vorschläge zu prüfen;
3. Beratung mit Vorschlägen zu einer Hilfeplanung mit stellvertretender Ausführung, da der/die Betroffene selbst oder seine/ihre Familie den Hilfeplan nicht mit befriedigendem Ergebnis umsetzten kann.

Im Fall des zuletzt genannten Betreuungsausgangs wird ein Fallmanagement erforderlich, welches stets dann greift, wenn sich der Patient in vielschichtigen Problemsituationen

befindet, mit mehreren notwendigen Leistungserbringern und Kostenträgern kommunizieren muss, der Patienten selbst oder betreuende Angehörige den Angebotsmarkt nicht selbst erschließen bzw. steuern kann, eine psychosoziale Begleitung notwendig wird und sich eine längerfristige Problemlage abzeichnet.

Der geplante Beratungsprozess, die im Assessment aufgenommenen Patientenmerkmale sowie die vom Arzt bereitgestellten Informationen werden nach erfolgtem Patientenkontakt an die Pharmazeuten pseudonymisiert (d.h. ohne Stammdaten, lediglich mit einer Studien-ID gekennzeichnet) weitergeleitet. Anhand dieser Daten wird die Pharmakotherapie mit Hilfe eines standardisierten Ablaufs überprüft. So wird z.B. auf Wechselwirkungen, Doppelverordnungen, Therapiedauer und Dosierungen geachtet. Weiterhin erfolgt eine Prüfung auf alters- und indikationsgerechte Verschreibung sowie nach geschlechtsspezifischen Kriterien. Für diese Überprüfung ist ein Zeitrahmen von 4 Stunden vorgesehen, basierend auf den Erfahrungen aus dem Projekt „Optimierung der Arzneimitteltherapiesicherheit von Bewohnern von Alten- und Pflegeheimen durch pharmazeutische Betreuung“, durchgeführt von Frau Waltering 2009 – 2011 im Auftrag des Landesinstituts für Gesundheit und Arbeit NRW. Die Ergebnisse der Überprüfung werden in einem „pharmaceutical care plan“ zusammengestellt. Dieser Plan umfasst die Empfehlungen zur möglichen Optimierung des Medikationsregimes. Diese Empfehlungen sind zu belegen an Hand von aktuellen, nationalen und internationalen Leitlinien, offiziellen Therapieempfehlungen und mit aktueller, wissenschaftlicher Literatur. Ebenfalls soll für die Empfehlung auf die Priscus-Liste, Beer`s Kriterien oder START und STOPP-Kriterien zurückgegriffen werden.

Die Information des Arztes erfolgt in Form einer SOAP-Mitteilung, einer in der Medizin üblichen und standardisierten Form der Informationsweitergabe und umfasst folgende Punkte:

S: Subjektive Einschätzung des Patienten/der Patientin und seiner/ihrer Probleme (→ Erhebung hier auch durch den Pflegestützpunkt)

O: Symptome und Beschwerden werden in diesem Punkt „objektiviert“ und durch Labordaten (Blutdruck, SCr, Cholesterinwerte, BG, HbA1c, Hb, UA, WBC, RBC etc.) untermauert, sowie Beurteilung des Pflegestützpunktes

A: beim sog. „Assessment“ werden Informationen und Befunde kritisch bewertet und Probleme definiert. Damit lassen sich Patientenprobleme priorisieren und die Gefährdung eines Patienten/einer Patientin feststellen

P: der „Plan“ enthält dann die Empfehlungen zur Änderung der Pharmakotherapie und für weitere soziale Maßnahmen. Zusätzlich können im Plan auch Hinweise zum Monitoring bestimmter Parameter aufgenommen werden

Die beim Assessment unter dem Punkt „S“ erwähnten Probleme werden in verschiedene Kategorien eingruppiert. Diese Klassifizierung kann z.B. an Hand der DRP Checkliste (DRP = Drug Related Problem) des Pharmaceutical Care Networks Europe „PCNE“ erfolgen.

Nach der Übermittlung der Empfehlung an den Arzt erfolgt die Dokumentation im CRF (Case Report Form), ob die Empfehlungen angenommen wurden. Die folgenden Kategorien werden zur Auswertung verwendet:

- Empfehlung wurde abgelehnt, weil sie für medizinisch falsch gehalten wird
- Empfehlung wurde aus anderen Gründen abgelehnt
- Empfehlung aus Kostengründen abgelehnt
- Empfehlung wurde angenommen
- Empfehlung wurde teilweise übernommen

Für die Erstellung und Übermittlung der Empfehlung und Informationen an den behandelnden Hausarzt ist ein Zeitfenster von ca. 14 Tagen vorgesehen. Rücksprachen zwischen Pharmazeut und Arzt sind jederzeit auf Wunsch des Arztes möglich, die Identifikation des jeweiligen Patienten und die patientenspezifische Kommunikation erfolgt dabei jedoch ausschließlich auf der Basis der Studien-ID.

Nach 6 Monaten wird ein sogenanntes „Folge-MTM“ wie bereits beschrieben durchgeführt. Die Änderungen und Ergebnisse werden bewertet, diskutiert und dokumentiert. Weiterhin erfolgt eine erneute Erhebung zur Lebensqualität. Der direkte Kontakt zum Patienten ist für die Pharmazeuten zu keinem Zeitpunkt gegeben. Alle Daten liegen stets pseudonymisiert vor.

2.3.3 Informationsaustausch zwischen den Professionen

Die folgende Tabelle stellt die zwischen den Professionen auszutauschenden Informationen dar. Grau gekennzeichnete Zellen beinhalten Daten, die pseudonymisiert übermittelt werden und betreffen vor allem die Schnittstellen Pflege- und Wohnberatung – Pharmazeut sowie bei Bedarf Arzt – Pharmazeut.

Tabelle 2: Inhalte des Datenaustauschs zwischen den Professionen

**Schnittstellen**	**Informationen**
Arzt → PuW	Kontaktdaten des Patienten: Name, Adresse, Telefonnummer;<br>Patientencharakteristika: Alter, Geschlecht, Größe, Gewicht;<br>Diagnosen;<br>Medikationsplan;

	Medikationshistorie der letzten sechs Monate (nur zu Beginn des Medikationsmanagement);<br>Vom Patient berichtete UAWs<br>Verordnungen über Heil- und Hilfsmittel;<br>Vitalparameter: Puls, Blutdruck;<br>Letzt verfügbares Labor;<br>Allergien und Unverträglichkeiten;<br>Einschätzung des Kognitiven Status;<br>Lebensqualität des Patienten;<br>Einschätzung der Sturzgefahr;<br>Schmerzbewertung;<br>Krankenhausaufenthalte des Patienten und Ursachen
PuW → Pharmazeut	Patientencharakteristika: Alter, Geschlecht, Größe, Gewicht;<br>Diagnosen, Einschätzung des Gesundheitszustandes;<br>Medikationsplan;<br>Ergänzungen des Medikationsplans durch weitere durch den Patienten eingenommene Arzneimittel (z.B. Facharztverordnungen);<br>Selbstmedikation der Patienten;<br>Informationen zum Medikationsmanagement des Patienten: Zeit und Regelhaftigkeit der Einnahme, Therapietreue und Abweichungen vom Medikationsplan, Lagerung der AZM, Handhabungsprobleme, Zufriedenheit mit der Medikation<br>Medikationshistorie der letzten sechs Monate (nur zu Beginn des Medikationsmanagement);<br>Vom Patienten wahrgenommene, unerwünschte Arzneimittelwirkungen<br>Verordnungen über Heil- und Hilfsmittel;<br>Vitalparameter: Puls, Blutdruck;<br>Letzt verfügbares Labor;<br>Allergien und Unverträglichkeiten;<br>Ernährungszustand des Patienten, PEG, Ausscheidungen;<br>Einschätzung des Kognitiven Status;<br>Tagesstruktur des Patienten<br>Rauchverhalten und Alkoholkonsum<br>Lebensqualität des Patienten;<br>Einschätzung der Sturzgefahr;<br>Schmerzbewertung;<br>Soziale Einbindung des Patienten;<br>Krankenhausaufenthalte des Patienten und Ursachen;<br>Pflegestufe des Patienten;<br>Ergebnisse des Durchgeführten Assessments nach SGB XI:

	Wohnumfeld, personell, Heil- und Hilfsmittelbedarf, (Lebens)Ziele des Patienten
Pharmazeut → PuW	Ergebnisse des Medikationsreview in Form des SOAP-Schemas (siehe Kapitel 2.3.2);<br>Hinweise zu Auswirkungen der AZM, welche für die Arbeit der Pflege- und Wohnberatung von Belangen sein könnten: Einnahmeverhalten, Auswirkungen auf die Gesundheitszustand des Patienten
PuW → Arzt	Ergebnisse des Medikationsreview der Pharmazeuten in Form des SOAP-Schemas ergänzt um die Assessment-Ergebnisse der PuW: AZM-Anmerkungen z.B. Interaktionseffekte, Angaben zur Selbstmedikation der Patienten, Lebensgewohnheiten, Verordnungsvorschläge Heil- und Hilfsmittel, etc.
Arzt → Pharmazeut	Schriftliche oder telefonische Absprachen bzgl. der unterbreiteten Vorschläge

PuW: Pflege- und Wohnberatung; AZM = Arzneimittel

2.4 Zielsetzung der Studie

Zielsetzung der Studie ist es, nachzuweisen, dass das geplante professionsübergreifende Medikationsmanagement im Vergleich zu den in der Versorgungspraxis bestehenden Kooperationen der Gesundheitsberufe geeignet ist, die Versorgung multimorbider Patienten mit Polypharmazie patientengerechter, sicherer und kosteneffektiver zu gestalten.

Eine patientengerechte Versorgung liegt dann vor, wenn die Arzneimitteltherapie eine enge Patienten- bzw. Bedarfsorientierung aufweist (z.B. ist die benötigte Unterstützung bei der Einnahme der Medikamente gegeben), damit zu einer verbesserten Therapietreue führt und eine ggf. vorliegende Über-, Unter- oder Fehlversorgung des Patienten vermindert werden kann. Die Sicherheit der Arzneimitteltherapie soll durch die Vermeidung potentiell inadäquater Arzneimittel sowie durch das Aufdecken von Interaktionseffekten zwischen verordneten Präparaten verbessert werden. Die hierdurch reduzierte Anzahl an unerwünschten Arzneimittelereignissen und -wirkungen sowie die damit ggf. einhergehende Vermeidung von Krankenhausaufenthalten und weiterer Ressourcenverbräuche im Gesundheitswesen könnten das professionsübergreifende Medikationsmanagement trotz seiner Eigenkosten als kosteneffektivere Versorgungsform im Vergleich zu einer Behandlung ohne Informationsaustausch zwischen den Professionen zeigen.

Neben der Kosten-Wirksamkeit des Medikationsmanagements im Rahmen der Krankenbehandlung nach SGB V soll darüber hinaus analysiert werden, welche Effekte sich durch die frühzeitige Inanspruchnahme der Pflege- und Wohnberatung im Zuge des SGB XI ergeben. Hierfür werden die über die Pflegeversicherung in Anspruch genommenen

Leistungen der Interventions- und Kontrollgruppe, die Zeit bis zum Wechsel in eine höhere Pflegestufe sowie der Verbleib in der Häuslichkeit miteinander verglichen.

Zudem soll in einem qualitativen Forschungsansatz erörtert werden, ob und unter welchen Bedingungen die Zusammenarbeit der Professionen in der Regelversorgung fortgeführt werden kann.

3 STUDIENDESIGN

3.1 Studientyp

Zur Beurteilung der Wirksamkeit und der Kosten des professionsübergreifenden Medikationsmanagement bei multimorbiden Patienten mit Polypharmazie wird eine prospektive, cluster-randomisierte, kontrollierte Studie im Stepped Wedge Design („Wartegruppendesign“) durchgeführt. Hierfür werden die teilnehmenden Studienpraxen nach Abschluss der Rekrutierungsphase in drei Gruppe randomisiert, von denen je eine zu den Studienzeitpunkten t1 (nach Abschluss der Rekrutierungsphase), t2 (t1 + 3 Monate) und t3 (t1 + 6 Monate) in die Konzeptgruppe wechselt.

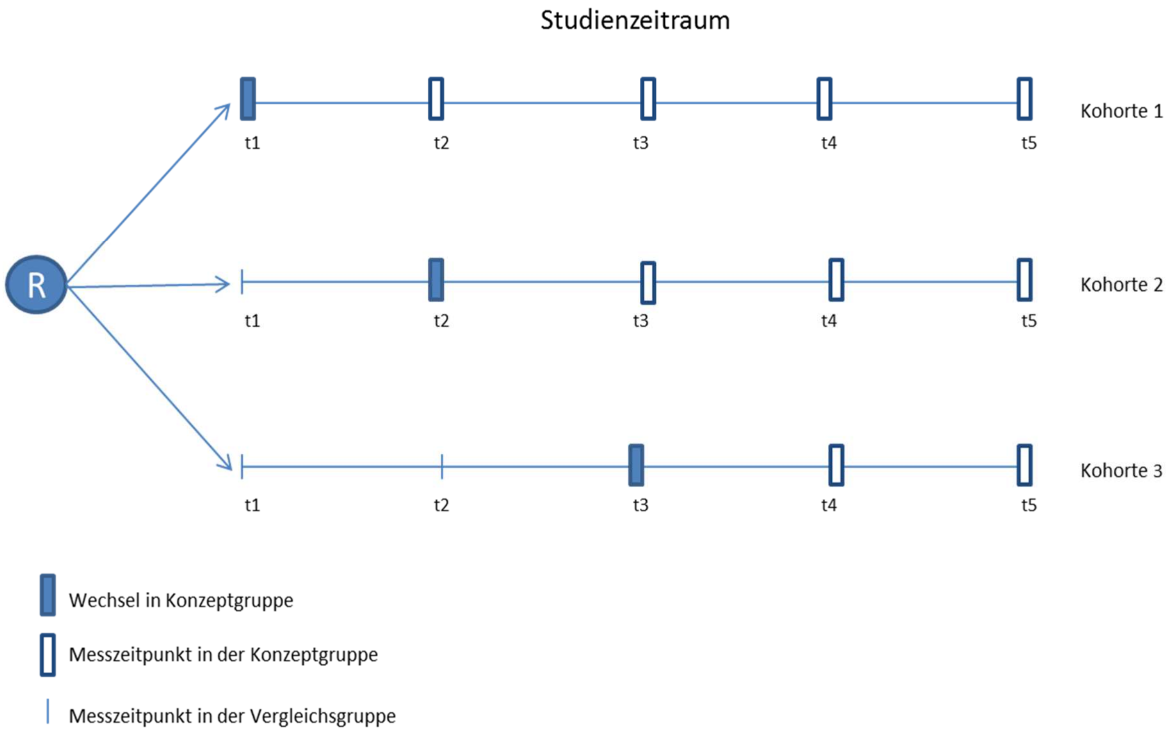

3.2 Studienpopulation

In die Studien werden Patienten eingeschlossen, die an Multimorbidität leiden, über mindestens eine chronische Erkrankung verfügen und zudem eine Polypharmazie aufweisen.

3.2.1 Einschlusskriterien

- Alter ≥ 65 Jahre
- mind. 3 chronische Erkrankungen aus 2 verschiedenen Organsystemen

  - die Organsysteme werden Anhand der Kapitel der ICD-10 Klassifizierung definiert
  - für Chronische Erkrankungen gilt eine Liste mit 46 Erkrankungen (siehe Anhang 1)
- mind. ein kardiovaskuläre Erkrankung
- eine der Erkrankungen muss mindestens bereits seit drei Quartalen der letzten 12 Monate bestehen (= Chroniker)
- mind. ein Arztbesuch in jedem der letzten drei Quartale
- fünf und mehr Dauermedikationen (> 3 Monate) mit systemischen Effekten
- unterschriebene Teilnahmeerklärung
- Fähigkeit, ggf. mit Unterstützung Fragebögen zu beantworten

3.2.2 Ausschlusskriterien

- Erkrankung, die eine Lebenserwartung weniger als 12 Monate bedingt
- Teilnehme an einer anderen klinischen Studie innerhalb der letzten 30 Tage

3.3 Teilnehmende Studienzentren

Die Studie wird im Kreis Steinfurt und in der Stadt Ahlen durchgeführt. In der Modellregion Ahlen nehmen niedergelassene Hausärzte an der Patientenrekrutierung und Dokumentation teil. Alle zum Stadtgebiet Ahlen gehörigen und hausärztlich tätigen Praxen wurden hierfür im Vorfeld der Studienplanungen durch den Bürgermeister mit der Bitte um Teilnahme angeschrieben. Die Auswahl der Studienpraxen erfolgte nicht systematisch, sondern auf freiwillige Entscheidung der Praxisinhaber hin. Im Kreis Steinfurt erfolgte keine öffentliche Ansprache der Arztpraxen, da hier bereits bei Beantragung des zum Projekt gehörigen Förderantrages ein Ärztenetz mitgewirkt hat.

Es wurden bewusst unterschiedliche regionale Strukturen als Studiensetting ausgewählt, um Prozessunterschiede bei der Durchführung des Konzepts sowie bei seiner späteren Implementierung erfassen zu können.

3.4 Randomisierung

Die Randomisierung in die Interventions- oder Kontrollgruppe erfolgt auf Cluster-, d.h. auf Praxisebene. Die Praxen erhalten zum Studienbeginn eine Zentrumsnummer, welche sowohl Teil der Patientenidentifikationsnummer ist als auch zur Kennzeichnung der Praxen in der Randomisierungsliste dient. Nach Abschluss der Rekrutierungsphase wird für alle Zeitpunkte (t1, t2, und t3), an welchen Praxen aus der Vergleichsgruppe in die Interventions-

/Konzeptgruppe wechseln, eine separate Randomisierungsliste mit den entsprechenden Zentrumsnummern vom IMSIE erstellt und in einzelnen Umschlägen versiegelt. Dabei wird je Modellregion stets die gleiche Anzahl an Studienpraxen in die Konzeptgruppe übergehen.

Ist im Studienverlauf ein Randomisierungszeitpunkt erreicht, wird der für diesen Zeitpunkt vorgesehene Umschlag geöffnet und die jeweiligen Zentren per Fax über ihren Wechsel in die Interventionsgruppe benachrichtigt.

3.5 Studienablauf

3.5.1 Patientenrekrutierung

Zu Beginn des Rekrutierungszeitraums stellt der teilnehmende Arzt auf der Basis der Ein- und Ausschlusskriterien eine alphabetische Liste aller für eine Studienteilnahme potentiell in Betracht kommenden Patientinnen und Patienten aus seinem Patientenstamm zusammen. Die Liste enthält eine fortlaufende Nummerierung beginnend bei eins (Praxis-ID), Nachnahme und Vorname des Patienten, das Geschlecht sowie das Geburtsdatum und dient neben der Auswahl von Studienpatienten gleichzeitig zur praxisinternen Identifikation der Studienteilnehmer. Die Hausarztpraxis leitet darauf hin die Anzahl der potentiell zur Verfügung stehenden Patienten, das Geschlecht und das Geburtsdatum an das IMSIE weiter, persönliche Daten des Patienten (Name, Vorname) verbleiben in der Arztpraxis und sind nur für den behandelnden Arzt sowie das Praxispersonal einsehbar. Computerbasiert erfolgt im IMSIE nun die Ziehung einer Zufallsstichprobe, die Zusammenstellung einer Rekrutierungsliste bestehend aus der in der Praxis vergebenen Praxis-ID (Zentrumsnummer + fortlaufende Nummer) sowie einer Studie-ID (Zentrumsnummer + Nummer von 01 bis 40) und der Versand dieser an den behandelnden Arzt. Jedes Studienzentrum erhält eine Rekrutierungsliste mit 40 Patienten, von welchen 20 eingeschlossen werden sollen.

Erscheint ein auf der Rekrutierungsliste befindlicher Studienpatient im Rahmen seiner Regelversorgung und im Rekrutierungszeitraum bei seinem behandelnden Hausarzt, wird er über die Studieninhalte sowie die Abläufe informiert und im Falle seiner Teilnahmebereitschaft um Unterschrift der Patienteneinverständnis- und Datenschutzerklärung gebeten. Zeitgleich erhält die Bergische Universität Wuppertal das Einschlussfax des Patienten, welches die Studie-ID des Patienten, Geburtsmonat und -jahr, das Geschlecht sowie das Einschlussdatum enthält.

3.5.2 Studien- und Dokumentationsverlauf

Bei Einschluss des Patienten in die Studie erfolgt zunächst eine Basiserhebung, bei der alle Baseline-Werte des Patienten vom Arzt und mit Hilfe von Selbstbeurteilungsintrumenten vom Patienten erfasst werden (siehe Kapitel 3.7). Zudem werden die Diagnose- und Medikationshistorie der Patienten sowie die in Anspruch genommenen Gesundheitsleistungen sechs Monate retrospektiv dokumentiert.

Nach Ablauf der Rekrutierungszeit (4 Monate nach Studienstart) aktualisiert der Arzt Diagnose und Medikationsdaten des Patienten. Praxen, die zu diesem Zeitpunkt in die Konzeptgruppe wechseln, übermitteln darauf hin die für das Erst-Assessment benötigten Parameter (siehe Kapitel 3.5.3) an die Pflege- und Wohnberatung. Ca. ein Monat nach dem Wechsel der jeweiligen Praxen in die Interventionsgruppe ist vom behandelnden Arzt in den Studienunterlagen anzugeben, welche Empfehlungen/Informationen der Pflege- und Wohnberatung und der Pharmazeuten in der Routinebehandlung des Patienten umgesetzt worden. Alle 3 Monate gibt der Arzt jeweils erneut die Diagnose- und Medikationsdaten der Patienten an die Pflege- und Wohnberatung zum Erst-, Follow-up-Assessment bzw. Abschluss-Assessment weiter. Nach Rückmeldung der Assessment-Ergebnisse wird die Empfehlungsverwendung jeweils in der Routineversorgung vom Arzt dokumentiert.

Das gewählte Stepped Wedge Design sieht vor, dass nach Ablauf der Rekrutierungsphase alle drei Monate randomisiert Praxen von der Vergleichs- in die Konzeptgruppe wechseln bzw. Diagnose- und Medikationsdaten zwischen den Professionen ausgetauscht werden. Die Dokumentationen in den Studienunterlagen durch den Arzt erfolgen in derselben Frequenz. Dies ist notwendig, um die aktuellen Diagnose- und Medikationsdaten mit geringem Zeitverzug an die Pflege- und Wohnberatung weiterleiten zu können. Weiterhin wird dadurch gewährleistet, dass für Patienten, deren Praxen sich noch in der Kontrollgruppe befinden, Vergleichswerte zu jedem Medikations-Assessment der Pharmazeuten für die spätere Auswertung vorliegen. Für die dreimonatigen Dokumentationen des Arztes sind jedoch keine studieneigenen Arzt-Patienten-Termine notwendig. Vielmehr aktualisiert der Arzt auf der Basis der Patientenakte die Angaben auf den Dokumentationsbögen.

Neben den Inhalten der Patientenakte werden zusätzliche Evaluationsparameter zum Gesundheitszustand des Patienten (z.B. Mobilitätstest nach Tinetti, SF-12, etc.; siehe Kapitel 3.7) vom Arzt und vom Patienten alle sechs Monate sowie zum Studienende erhoben. Die Dokumentation erfolgt im Rahmen der normalen Behandlungstermine. Um zu gewährleisten, dass von allen Studienpatienten über die Patientenakte hinausgehende Dokumentationen möglichst lückenlos für die Auswertung zur Verfügung stehen, wurden die Dokumentationszeiträume mit sechs Monaten entsprechend groß gewählt, da bei multimorbiden Patienten mit mindestens einem Arztbesuch innerhalb dieser Zeit zu rechnen

ist. Abbildung 1 fasst den Studien- und Dokumentationsablauf in der Praxis des Arztes zusammen.

Die Studie wird durch 20 minütige Telefoninterviews des Patienten ggf. mit Unterstützung durch einen Angehörigen im dreimonatigen Abstand beginnend mit Ende der Rekrutierungszeit begleitet. Die Interviews werden nach Einverständnis der Studienpatienten durch die Bergische Universität Wuppertal durchgeführt. Sie dienen zur Erhebung des funktionalen Status, der Gesundheitskompetenz, sozialer Determinanten (wie z.B. Selbstmanagement, Raucherverhalte), zur Dokumentation von Ressourcenverbräuchen der Patienten in der gesamten Krankenversorgung (SGB V) und Pflege (SGB XI) sowie zur Selbsteinschätzung ihrer bestehenden Polypharmazie (z.B. Belastung durch die Therapie, berichtete Therapietreue).

Abbildung 1: Studien- und Dokumentationsablauf

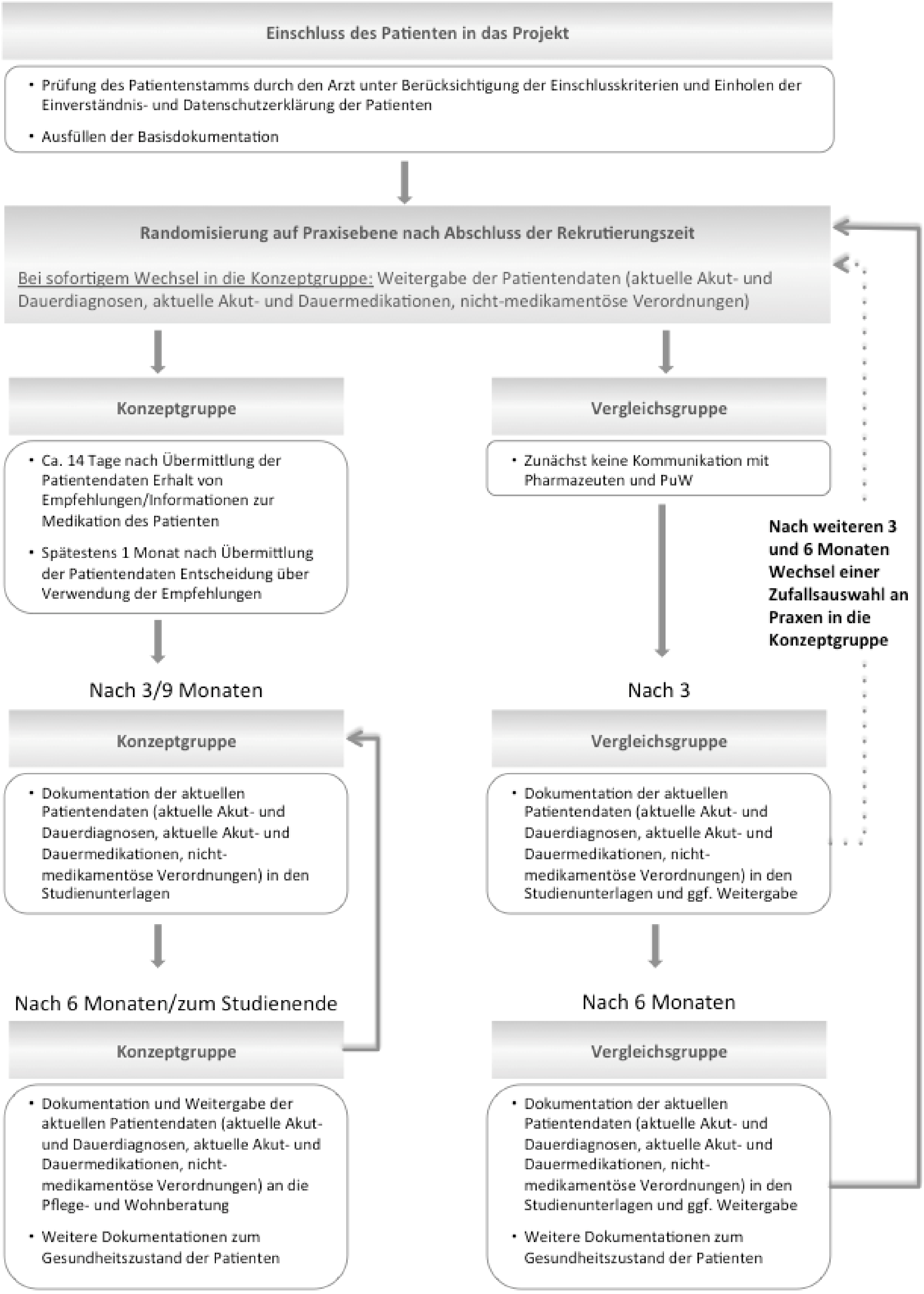

3.5.3 Beobachtungsdauer

Vor Beginn des Studienzeitraums erfolgt zunächst eine Rekrutierungsphase. Die Patientenrekrutierung durch den Arzt (Last patient 1st visit) sollte innerhalb von vier Monaten abgeschlossen sein. Der Studienzeitraum beträgt 12 Monate. Er beginnt mit dem Wechsel der ersten Arztpraxen in die Konzeptgruppe. Die Abgabe der letzten Studienunterlagen zur Dateneingabe durch die Bergische Universität Wuppertal kann bis 18 Monate nach dem Start der Patientenrekrutierung erfolgen.

3.6 Zielparameter

3.6.1 Primäre Zielparameter

Primärer Zielparameter ist die Veränderung der Angemessenheit der Arzneimitteltherapie, gemessen mit Hilfe des Medication Appropriateness Index (MAI) Scores. Betrachtet wird der Unterschied zwischen dem mittleren Gesamtscore vor und nach dem Wechsel der Patienten in die Konzeptgruppe.

1. Medication Appropriateness Index (MAI) Score

   Der MAI-Score wurde zur Bestimmung des primären Zielparameters ausgewählt, da er eine umfassende und valide Beurteilung der Angemessenheit der Medikation gewährleistet. Zudem zählt das Instrument als Standard in der Evaluation des Medication Therapy Managements [35].

   Jedes vom Patienten eingenommene Arzneimittel wird durch 10 Kriterien auf seine Angemessenheit hin bewertet. Die 10 Kriterien sind mit Hilfe von Bewertungsanweisungen und Beispielen operationalisiert und werden auf einer 3-Punkte umfassenden Skala (1-angemessen, 2-marginal angemessen, 3-unangemessen) in ihrem Ausprägungsgrad durch zwei unabhängige Reviewer beurteilt sowie anschließend Kriterien-spezifisch gewichtet. Als Gewichtung werden die Werte aus der Publikation von Samsa und Kollegen 1994 zugrunde gelegt [36]. Jedes Arzneimittel kann einen Score von bis zu 18 Punkten erzielen, wobei mit steigender Punktzahl ein zunehmender Grad an Unangemessenheit ausgedrückt wird. Pro Patient wird zudem ein Gesamtscore gebildet [37]. Folgende Tabelle stellt die Kriterien und Wertigkeiten zusammenfassend dar.

Kriterium	Gewichtung
Indikation	3
Effektivität	3
Dosierung korrekt	2
Applikationsweg korrekt	2
Applikationsweg praktikabel	1
Arzneimittelwechselwirkung	2
Wechselwirkung mit Begleiterkrankung	2
Doppelverordnung	1
Dauer der Anwendung	1
(Arzneimittel kosteneffektiv)	(1)

3.6.2 Sekundäre Zielparameter

1. START-/STOPP-Kriterien
2. PCNE-ABP

Als weitere sekundäre Zielparameter gelten die *Lebensqualität*, erhoben durch den EQ5-D sowie den SF-12, und das *Kosten-Effektivitäts- sowie das Kosten-Nutzwert-Verhältnis* zwischen Interventions- und Kontrollgruppe. Für die Bestimmung des Kosten-Nutzwert-Verhältnisses werden die Perspektive der GKV sowie die der GKV-Versichertengemeinschaft eingenommen.

Als sekundäre Zielparameter werden des Weiteren die folgenden Endpunkte, berücksichtigt.

- Komplexität der Arzneimitteltherapie (Medication Regimen Complexity Index (MRCI), Anzahl der Arzneimittel, Anzahl der Einzeldosen pro Tag)
- Berichtete Therapieadhärenz (Morisky und MARS)
- Belastung durch die Therapie
- Anzahl berichteter (potentieller) UAWs
- Funktionaler Status
- Sturzgefahr

- Selbstberichtete Gesundheit
- Medikationskosten
- Krankenhaustage
- Krankheitskosten aus der Perspektive des Hausarztes
- Qualität der Versorgung vulnerabler Patientengruppen (ACOVE)

Der Effekt eines frühzeitigen Einsatzes der Pflege- und Wohnberatung wird über die Zeit bis zum Wechsel in eine höhere Pflegestufe und den Verbleib in der Häuslichkeit bewertet.

Eine Bewertung der einzelnen Prozesse des Konzepts erfolgt im Rahmen eines qualitativen Ansatzes am Ende der Studie.

3.7 Beobachtungsparameter

Tabelle 3: Beobachtungsparameter und Dokumentationszeitpunkt

**Beobachtungsparameter**	**t 0**	**t 1**	**t 2**	**t 3**	**t 4**	**t 5**
Patienteneinverständniserklärung und Datenschutz	A					
Ein- und Ausschlusskriterien	A					
Soziodemographische Merkmale						
Geburtsmonat/Jahr	A					
Geschlecht	A					
Familienstand, Haushaltsgröße, Wohnumstände		T	T	T	T	T
Bildungsabschluss und ehemaliger Beruf		T				
Versichertenstatus, Krankenkasse, Wahltarife	A	T	T	T	T	T
Migrantenstatus		T				
Einkommen (Einkommensarten, etwaige Höhe)		T				T
Morbidität						
Größe und Gewicht	A	A	A	A	A	A
Vitalparameter: Blutdruck, Herzfrequenz	A	A	A	A	A	A
Laborwerte (nach Verfügbarkeit)	A	A	A	A	A	A
Aktuelle Akut- und Dauerdiagnosen	A	A	A	A	A	A
Kardiovaskuläres Risiko	G	G	G	G	G	G

Allergien und Unverträglichkeiten	A					
Komplexität der Morbiditätsstruktur	A	A	A	A	A	A
Symptome potentieller unerwünschter Arzneimittelwirkungen	A	A	A	A	A	A
Lebensqualität	P	PT	PT	PT	PT	PT
Depression	P	PT	PT	PT	PT	PT
Pflegestufe	A	T	T	T	T	T
Funktionaler Status						
Einschränkungen bei Aktivitäten des täglichen Lebens		T	T	T	T	T
Sturzgefahr	A	A		A		A
Mobilität		T	T	T	T	T
Kognitiver Status	A			A		A
Schmerz	P	T	T	T	T	T
Selbstbewertete Gesundheit	P	T	T	T	T	T
Sehen und Hören		T	T	T	T	T
Gesundheitsverhalten und soziale Unterstützung						
Selbstmanagement		T		T		T
Rauchverhalten und Alkoholkonsum	P	T				T
Soziale Einbindung und Unterstützung		T		T		T
Arzneimitteltherapie						
Akutmedikation: Wirkstoff, Wirkstärke, Dosis etc.	A	A	A	A	A	A
Dauermedikation: Wirkstoff, Wirkstärke, Dosis etc.	A	A	A	A	A	A
Nicht-Medikamentöse Therapien	A	A	A	A	A	A
Komplexität der Arzneimitteltherapie		Ph	Ph	Ph	Ph	Ph
Potentiell inadäquate Medikation (PIM, MAI-Score, START-STOPP, PCNE-ABP)		Ph	Ph	Ph	Ph	Ph
Berichtete Therapieadhärenz		T	T	T	T	T
Arzneimittelbezogene Probleme		Ph	Ph	Ph	Ph	Ph
Belastung durch die derzeitige Arzneimitteltherapie		T	T	T	T	T
Inanspruchnahmeverhalten						
Anzahl der Haus- und Facharztbesuche	A	AT	AT	AT	AT	AT

Anzahl Krankenhausaufenthalte, Dauer und Ursache	A	AT	AT	AT	AT	AT
weitere Inanspruchnahme von Gesundheitsleistungen	A	AT	AT	AT	AT	AT
Leistungen im Rahmen des SGB XI		T	T	T	T	T
Versorgungsstruktur						
Praxisorganisation (z.B. Einzel- oder Gemeinschaftspraxis)	A					
Qualität der Versorgung vulnerabler Patientengruppen	A					A
Erwartungen an das Medikationsmanagement	Pr					
Bewertung der Zusammenarbeit				Pr		Pr

T_0: Baseline; T_1: Ende der Rekrutierungszeit; T_2: Ende Rekrutierungszeit + 3 Monate; T_3: Ende Rekrutierungszeit + 6 Monate; T_4: Ende Rekrutierungszeit + 9 Monate; T_5: Ende Rekrutierungszeit + 12 Monate/Studienende;
A = durch den Arzt, P = durch den Patient, T = im Telefoninterview; Ph = durch Pharmazeut; G = aus anderen Beobachtungsparametern generierbar; Pr = alle beteiligten Professionen

4 STUDIENORGANISATION

4.1 Platzierung der Studie

Die Platzierung der Studie erfolgt in einem persönlichen Initialbesuch durch einen Mitarbeiter der Bergischen Universität Wuppertal, in dem der teilnehmende Arzt bzw. das Praxisteam in die Modalitäten der Durchführung eingewiesen werden und die Übergabe der Dokumentationsunterlagen erfolgt. Weiterhin findet die Besprechung des Protokolls, Klärung von Fragen und Unterzeichnung der Teilnahmeerklärung statt.

4.2 Betreuung der teilnehmenden Praxen/Monitoring

Die Studie wird regelmäßig durch einen verantwortlichen Monitor der Bergischen Universität Wuppertal hinsichtlich der Qualität der erhobenen Daten betreut. Zusätzlich wird telefonischer Kontakt mit den teilnehmenden Praxen gehalten. Die Studienärzte sind damit einverstanden, dass der Monitor Einsicht in das Quellmaterial für die Daten der Studie nimmt.

4.3 Newsletter

Ein Newsletter wird dreimal im Laufe der Studie an die teilnehmenden Ärzte versendet: am Ende der Rekrutierungsphase, nach der Zwischenauswertung und nach Erstellung des Abschlussberichtes. Der Versand kann je nach Arztwunsch per Post, Fax oder E-Mail erfolgen und dient zur Information des Arztes über den aktuellen Stand der Studie.

5 BIOMETRIE

5.1 Fallzahlschätzung

Die Fallzahlschätzung für die clusterrandomisierte Studie im Stepped Wedge Design wird wie bei Woertman et al., 2013 [38] beschrieben durchgeführt. Es wird angenommen, dass pro Praxis 20 Patienten eingeschlossen werden können, auf der Basis ähnlicher Studien ist davon auszugehen, dass der ICC 0,05 beträgt.

Da die Studie im Vergleich zu internationalen Untersuchungen vor allem patientenrelevante Effekte des Medikationsmanagements in den Mittelpunkt stellen möchte, liegen weder nationale noch internationale Forschungsergebnisse vor, die den erwarteten Interventionseffekt abschätzen. Für die gegebene Fragestellung ist jedoch davon auszugehen, dass eine Effektstärke von 0,25 Standardabweichungen einen klinisch und sozial relevanten Unterschied in der der Prävalenz angemessen eingesetzter Arzneimittel (gemessen durch den MAI-Score) beschreibt.

In einem Patientenkollektiv von 502 auswertbaren Patienten lässt sich diese Effektstärke mit Hilfe eines zweistichproben T-Tests mit einer Power von $1-\beta = 80\%$ bei einem Signifikanzniveau $\alpha = 5\%$ zweiseitig nachweisen. Mit Hilfe des berechneten Designfaktors von 0,383 für die clusterrandomisierte Studie im Stepped Wedge Design [38] folgt daraus, dass 192 Patienten benötigt werden.

Es wird eine Drop-out Rate von maximal 20% erwartet, so dass insgesamt 240 Patienten eingeschlossen werden sollen. Diese verteilen sich gleichmäßig auf die beiden Modellregionen. Insgesamt werden 12-16 Praxen benötigt.

5.2 Statistische Auswertung

5.2.1 Definition von Auswertungskollektiven

Primäre Auswertungspopulation ist die Intention to Treat (ITT) Population. Patienten, die unmittelbar nach der Rekrutierung und vor der eigentlichen Umsetzungsphase des Medikationsmanagements aus der Studie ausscheiden, werden als nicht verwertbar für die ITT-Population angesehen. Patienten, die im Laufe der Umsetzungsphase aus der Studie ausscheiden, werden in der ITT-Population analysiert. Des Weiteren wird eine unterstützende Per Protocol Analyse durchgeführt.

5.2.2 Beschreibung der Studienpopulation

Die gesamte Studienpopulation, sowie die einzelnen Interventionsgruppen werden deskriptiv bezüglich der soziodemographischer Parameter und den Baselinevariablen beschrieben.

5.2.3 Auswertung

Die primäre Zielgröße, der Medication Appropriateness Index (MAI) Score (Gemittelt über alle Medikamente eines Patienten), wird mit Hilfe eines Gemischten Modells unter Berücksichtigung aller verfügbaren Daten von allen Zeitpunkten analysiert [39]. Das Modell beinhaltet die festen Faktoren Studiengruppe und Zeit sowie zufällige Effekten zur Berücksichtigung der Clusterstruktur und der wiederholten Messungen innerhalb eines Individuums.

Die Sekundären Zielparameter werden analog mit Hilfe von gemischten Modellen ausgewertet. Weitere Analysen werden explorativ durchgeführt.

5.2.4 Subgruppenanalyse

Die folgenden Variablen sollen als Einflussfaktoren im Auswertungsmodell berücksichtigt werden, außerdem sollen für diese Parameter Subgruppenanalysen durchgeführt werden.

- Alter
- Gender
- Migrantenstatus
- Soziale Einbindung
- Komplexität der Morbiditätsstruktur

5.2.5 Umgang mit fehlenden Werten

Bei der primären Auswertung werden die fehlenden Werte nicht ersetzt. Das Gemischte Modell bietet die Möglichkeit bei der Auswertung alle verfügbaren Daten zu verwenden. Es ist zu erwarten, dass die fehlenden Werte Missing at Random (MAR) sind.
Des Weiteren werden Sensitivitätsanalysen durchgeführt. Dabei werden die fehlenden Werte ersetzt (etwa last observation carried forward).

5.2.6 Gesundheitsökonomische Evaluation

Zur Generierung von Erkenntnissen zur Wirtschaftlichkeit des professionsübergreifenden Medikationsmanagements werden das inkrementelle Kosten-Effektivitäts- sowie das inkrementelle Kosten-Nutzwert-Verhältnis zwischen Interventions- und Kontrollgruppe über einen Zeitraum von sechs Monaten bestimmt.

Die Kosten setzen sich dabei aus den Aufwendungen für die Durchführung des Medikationsmanagements und den auf Seiten der Patienten entstandenen Gesundheitsausgaben zusammen.

Um die Kosten aus verschiedenen Perspektiven beurteilen zu können, finden je nach Sichtweise unterschiedliche Kostenkomponenten Berücksichtigung:

- GKV-Perspektive: Ressourcenverbräuche, welche im Rahmen des SGB V erstattet werden.
- GKV-Versichertengemeinschaft: Ressourcenverbräuche, welche im Rahmen des SGB V erstattet werden sowie vom Patienten selbst getragen werden müssen.
- Gesamtgesellschaftliche Perspektive: Ressourcenverbräuche, welche im Rahmen des SGB V und SGB XI erstattet werden sowie aus gesamtgesellschaftlicher Sicht berücksichtigt werden müssen (z.B. Arbeitsausfälle der Angehörigen zur Unterstützung der Patienten)

Eine ausführliche Beschreibung der erhobenen Ressourcenverbräuche sowie das Vorgehen bei der Berechnung der „Unit Costs“ sind in einem eigenen gesundheitsökonomischen Analyseplan beschrieben.

Im Rahmen der Kosten-Effektivitäts-Analyse findet die Anzahl vermiedener arzneimittelbezogener Probleme sowie die Veränderung des MAI-Scores als Effektivitätsparameter Anwendung. Das inkrementelle Kosten-Nutzwert-Verhältnis wird als Kosten je QALY (Quality-adjusted life year) dargestellt. Die Nutzwerte werden hierfür über den EQ5-D bestimmt.

6 DATENMANAGEMENT

6.1 Patientenidentifikationsliste

Alle patientenbezogenen Daten werden in pseudonymisierter Form erfasst. Lediglich der Informationsaustausch zwischen behandelndem Arzt und Pflege- und Wohnberatung erfolgt in nicht pseudonymisierter Form, da das professionsübergreifende Medikationsmanagement den persönlichen Kontakt zwischen Patient und Pflege- und Wohnberatung vorsieht. Die Identifikation der Studienpatienten erfolgt mit Hilfe einer Studien-ID, welche auf allen Dokumentationsunterlagen vermerkt ist. Die Erstellung dieser Studien-ID erfolgt durch 4-stellige Patientennummern, zwei Stellen für das Zentrum, zwei Stellen für den Patienten. Jede teilnehmende Praxis führt eine Patientenidentifikationsliste mit dem vollen Namen aller potentiellen Studienpatienten sowie einer 6-stelligen Praxis-ID, zwei Stellen für das Zentrum, vier Stellen für den Patienten. Zudem erhält die Praxis für die Rekrutierung der Studienpatienten eine Rekrutierungsliste mit Praxis-ID und Studien-ID, welche nach Studienende gemeinsam mit der Patientenidentifikationsliste für 10 Jahre zu archivieren ist. Die Patienteneinverständniserklärung enthält nur die Praxis-ID, jedoch keine Studien-ID.

6.2 Datenerhebung

Die Datenerhebung in den teilnehmenden Praxen erfolgt in Papierform auf Dokumentationsbögen. Die Dokumentationsbögen liegen in Durchschreibsatz vor. Das Original verbleibt in der teilnehmenden Arztpraxis, die Kopie ist für die Studienzentrale bestimmt. Eine Anleitung zum Ausfüllen der Dokumentationsbögen erfolgt durch den verantwortlichen Monitor, der auch für diesbezügliche Fragen zur Verfügung steht. Korrekturen sind wie folgt vorzunehmen: Der falsche Eintrag wird mit einer einfachen Linie durchgestrichen, die korrekte Information daneben eingetragen und vom Arzt mit Datum paraphiert, und ggf. mit Angabe des Grundes der Korrektur versehen. Datenfelder, die wegen fehlender Information nicht ausgefüllt werden können, sind zu kommentieren.

Zur Messung der in Kapitel 3.7 beschriebenen Beobachtungsparameter werden validierte Erhebungsinstrumente und in großen Bevölkerungsumfragen bereits standardisierte Fragebatterien verwendet. Klinische Parameter sowie Angaben zu Arzneimitteln werden aus den Patientenakten in die Dokumentationsunterlagen übertragen. Die Erhebungsinstrumente sind in nachfolgender Tabelle aufgeführt.

Tabelle 4: Verwendete Erhebungsinstrumente

Beobachtungsparameter	Erhebungsinstrument
Geburtsmonat/Jahr	Übertrag aus Patientenakte
Geschlecht	Übertrag aus Patientenakte
Familienstand, Haushaltsgröße, Wohnumstände	Fragebatterien aus dem Sozioökonomischen Panel
Bildungsabschluss und (ehemaliger) Beruf	Fragebatterien aus dem Sozioökonomischen Panel
Versichertenstatus, Krankenkasse, Wahltarife	Fragebatterien aus dem Sozioökonomischen Panel
Migrantenstatus	Fragebogen nach Schenk et al. 2007
Einkommen (Einkommensarten, etwaige Höhe)	Fragebatterien aus dem Sozioökonomischen Panel
Größe und Gewicht	Übertrag aus Patientenakte
Vitalparameter: Blutdruck, Herzfrequenz	Übertrag aus Patientenakte
Laborwerte (nach Verfügbarkeit)	Übertrag aus Patientenakte
Aktuelle Akut- und Dauerdiagnosen	Übertrag aus Patientenakte
Kardiovaskuläres Risiko	SCORE (Systematic Coronary Risk Evaluation)
Allergien und Unverträglichkeiten	Übertrag aus Patientenakte
Komplexität der Morbiditätsstruktur	CIRS-G (Cumulative Illness Rating Scale - German)
Symptome potentieller unerwünschter Arzneimittelwirkungen	eigene Items
Lebensqualität	SF-12, EQ5-D
Depression	PHQ-9
Pflegestufe	eigene Items
Einschränkungen bei Aktivitäten des täglichen Lebens	ADL, iADL, VES-13
Sturzgefahr	Mobilitätstest nach Tinetti
Mobilität	FFB-Mot
Kognitiver Status	MMSE
Schmerz	GCPS (German Chronic Pain Scale)
Selbstbewertete Gesundheit	Fragebatterien aus dem Sozioökonomischen Panel
Sehen und Hören	Fragebatterien aus dem Sozioökonomischen Panel
Selbstmanagement	European Selfcare Behavior Scale
Rauchverhalten und Alkoholkonsum	Fragebatterien aus dem Sozioökonomischen Panel; AUDIT-C

Soziale Einbindung und Unterstützung	F-SOZU K14; SoS
Akutmedikation: Wirkstoff, Wirkstärke, Dosis etc.	Übertrag aus Patientenakte
Dauermedikation: Wirkstoff, Wirkstärke, Dosis etc.	Übertrag aus Patientenakte
Nicht-Medikamentöse Therapien	Übertrag aus Patientenakte
Komplexität der Arzneimitteltherapie	MRCI (Medication Regimen Complexity Index)
Potentiell inadäquate Medikation (PIM)	MAI-Score
Berichtete Therapieadhärenz	Morisky, MARS
Arzneimittelbezogene Probleme	PCNE-ABP
Belastung durch die derzeitige Arzneimitteltherapie	eigene Items
Anzahl der Haus- und Facharztbesuche	Übertrag aus Patientenakte
Anzahl Krankenhausaufenthalte, Dauer und Ursache	Übertrag aus Patientenakte
weitere Inanspruchnahme von Gesundheitsleistungen	Übertrag aus Patientenakte;<br>eigene Items
Leistungen im Rahmen des SGB XI	eigene Items
Praxisorganisation (z.B. Einzel- oder Gemeinschaftspraxis)	eigne Items
Qualität der Versorgung vulnerabler Patientengruppen	ACOVE (Assessing Care of Vulnerable Elders)
Erwartungen an das Medikationsmanagement	Fokusgruppen
Bewertung der Zusammenarbeit	Fokusgruppen

6.3 Datenverarbeitung

Die Datenerfassung erfolgt mit Hilfe einer SPSS-Datenbank. Die Eingabe erfolgt durch zwei Personen unabhängig voneinander (double data entry). Die Überprüfung der Richtigkeit der Daten erfolgt durch Range-, Validitäts- und Konsistenzchecks. Nicht plausible oder fehlende Daten können korrigiert oder ergänzt werden (Querymanagement). Die Queries müssen schriftlich erfolgen und werden gemeinsam mit den übrigen Dokumentationsunterlagen archiviert. Am Studienende wird nach Eingabe aller Eintragungen die Datenbank geschlossen. Dieser Vorgang wird dokumentiert.

6.4 Aufbewahrung der Studienunterlagen

Die Originale aller zentralen Studiendokumente werden im jeweiligen teilnehmenden Zentrum für mindestens 10 Jahre nach Erstellung des Abschlussberichtes aufbewahrt. Originaldaten der Studienpatienten (Patientenakte) werden ebenso 10 Jahre archiviert.

6.5 Datenschutz

Mit Hilfe eines Sicherheitskonzeptes wird der Schutz vor unbefugtem Zugriff und der Schutz vor Datenverlust sichergestellt und dafür Sorge getragen, dass die Bestimmungen des Datenschutzgesetzes eingehalten werden. Die Studiendaten sind vor fremden Zugriff geschützt und nur Mitarbeiter der Studie dürfen auf diese zugreifen. Diese Mitarbeiter sind zur Verschwiegenheit verpflichtet. Im Falle eines Widerrufes der Einwilligung durch den Patienten wird geprüft, inwieweit die gespeicherten Daten noch erforderlich sind. Nicht mehr benötigte Daten werden unverzüglich gelöscht.

Alle auf die Studie bezogenen Patientendaten werden ohne Nennung des Patientennamens der biometrischen Auswertung zugeleitet. Davon ausgenommen bleiben die Erklärungen zum Patienteneinverständnis, die in der Dokumentation der Arztpraxis verbleiben. Damit ist die Vertraulichkeit der persönlichen Daten gesichert und eine Identifikation einzelner Personen von Seiten Außenstehender nicht möglich.

7 GESETZLICHE GRUNDLAGEN UND ADMINISTRATIVE ASPEKTE

7.1 Ethische Grundsätze

7.1.1 Ethikkommission

Das vorliegende Studienprotokoll wird der zuständigen Ethikkommission an der Bergischen Universität Wuppertal vorgelegt. Für alle beteiligten Zentren wird ebenso die Zustimmung der beteiligten Ethikkommission (bei der Ärztekammer Westfalen-Lippe) beantragt. Bei Protokolländerungen werden die Ethikkommissionen informiert.

7.1.2 Aufklärung und Einwilligung der Patienten

Jeder Patient wird über die Studie, die Randomisierung und die Teilnahme genau aufgeklärt. Jeder Patient erklärt schriftlich seine Einwilligung zur Teilnahme an der Studie. Dem Patienten wird genügend Zeit gegeben, um vor Einschluss in die Studie über die Teilnahme zu entscheiden und offene Fragen zu klären. Darüber hinaus ist es dem Patienten möglich, zu jedem Zeitpunkt sein Einverständnis zurückzuziehen und die Studien abzubrechen. Ein Muster der Patienteninformation und Einwilligungserklärung sind als Anhang beigefügt.

7.1.3 Verwendung, Speicherung und Weitergabe von Daten

Die Patienten werden über die pseudonymisierte Weitergabe ihrer Daten und Verwendung für wissenschaftliche Auswertungen informiert.

7.1.4 Gesetzliche Grundlagen

Die Empfehlungen der Guten Klinischen Praxis – für Deutschland verbindlich umgesetzt durch die GCP-Verordnung 2004, die Leitlinien und Empfehlungen zur Sicherung von Guter Epidemiologischer Praxis (GEP) 2008 sowie das Memorandum III „Methoden für die Versorgungsforschung“ des Deutschen Netzwerkes Versorgungsforschung e.V. (DNVF e.V., 2009 und 2010) werden berücksichtigt.

7.2 Versicherung

Für die vorliegende Untersuchung ist kein Versicherungsschutz notwendig, da am Patienten keine Leistungen außerhalb der Regelversorgung im Rahmen des SGB V und SGB XI erfolgen. Die Intervention beschränkt sich ausschließlich auf den Informationsaustausch zwischen den Professionen.

7.3 Unerwünschte Ereignisse

Unerwünschte Ereignisse als Folge der Studie werden nicht erwartet, da es sich nicht um eine direkte Intervention handelt, sondern durch die professionsübergreifende Zusammenarbeit lediglich eine breitere Informationsbasis zur Situation des Patienten generiert wird. Therapeutische Entscheidungen werden nicht vorgegeben sondern verbleiben im Ermessen des Arztes.
Während der Studie auftretende unerwünschte Ereignisse werden im Rahmen der ärztlichen Dokumentation gemäß Regelversorgung der Studienteilnehmer festgehalten.

7.4 Finanzierung

Die Studie wird durch die Europäische Union sowie durch das Ministerium für Gesundheit, Emanzipation, Pflege und Alter des Landes Nordrhein-Westfalen gefördert.

7.5 Abschlussbericht und Publikation

Nach Abschluss der biometrischen Auswertung wird ein integrierter Bericht von der Projektleitung erstellt. Der Bericht enthält den statistischen Bericht, eine Auswertung des qualitativen Studienabschnitts und die Schlussfolgerung. Die Veröffentlichung der Ergebnisse erfolgt unabhängig von den Ergebnissen der Studie.

7.6 Einhaltung des Protokolls und Protokolländerungen

Das Studienprotokoll wird genau eingehalten. Jede vom teilnehmenden Arzt, der Pflege-und Wohnberatung sowie den Pharmazeuten zu vertretende Abweichung von den vorgesehenen Konzeptbestandteilen und Beobachtungszeitpunkten wird dokumentiert und begründet. Änderungen oder Ergänzungen des Studienprotokolls können nur von der Projektleitung veranlasst und autorisiert werden.

8 LITERATURVERZEICHNIS

1. Valderas JM, Starfield B, Sibbald B, Salisbury C, Roland M. Defining Comorbidity: Implications for understanding health and health services. The Annals of Family Medicine. 2009;7 (4):357-63.
2. Scheidt-Nave C, Richter S, Fuchs J, Kuhlmey A. Herausforderungen an die Gesundheitsforschung für eine alternde Gesellschaft am Beispiel "Multimorbidität". Bundesgesundheitsblatt. 2010;53 (5):441-50.
3. Fortin M, Bravo G, Hudon C, Vanasse A, Lapointe L. Prevalence of multimorbidity among adults seen in family practice. The Annals of Family Medicine. 2006;3 (3):223-8.
4. Wiesner G, Bittner E. Zur Inzidenz und Prävalenz von Mehrfachkrankheiten in Deutschland. Arbeitsmedizin, Sozialmedizin, Umweltmedizin. 2005;40 (9).
5. van den Bussche H, Koller D, Kolonko T, Hansen H, Wegscheider K, Glaeske G, et al. Which chronic diseases and disease combinations are specific to multimorbidity in the elderly? Results of a claims data based cross-sectional study in Germany. BMC Public Health. 2011;11 (1):101.
6. Gorenoi V, Schönemark MP, Hagen A. Instrumente zur Risikoprädiktion für kardiovaskuläre Erkankungen. In: DIMDI, editor. Schriftenreihe Health Technology Assessment. Köln: DIMDI; 2009.
7. Brenner G, Altenhofen L, Kerek-Bodden H, Koch H, Lang A, Weber I, et al. Behandlung von Patienten mit Kreislauferkrankungen in der Arztpraxis. Herz. 2000;25 (5):502-14.
8. Diagnosedaten der Patienten und Patientinnen in Krankenhäusern [database on the Internet]. Destatis. 2009.
9. Destatis. Sterbefälle 2009. [cited 2011 07.03.]; Available from: http://www.destatis.de/jetspeed/portal/cms/Sites/destatis/Internet/DE/Content/Statistiken/Gesundheit/Todesursachen/Tabellen/Content75/SterbefaelleInsgesamt,templateId=renderPrint.psml
10. Nöthen M, Böhm K. Krankheitskosten. Berlin: Robert Koch Institut; 2009; Available from: http://www.worldcat.org/oclc/503460510.
11. Schwabe U, Paffrath D. Arzneiverordnungs-Report. Aktuelle Daten, Kosten, Trends und Kommentare. Heidelberg: Springer Medizin; 2009.
12. Teweleit S, Kuschel U, Hippius M, Goettler M, Bornschein B. Manifestation und Präventionsmöglichkeiten unerwünschter Arzneimittelwirkungen (UAW) in der Pharmakotherapie von Herz-Kreislauf-Erkrankungen. Medizinische Klinik. 2001;96 (8):442-50.
13. Grimmsmann T, Himmel W. Polipharmacy in primary care practice: an analysis using a large health insurance database. Pharmacoepidemiol Drug Saf. 2009;18 (12):1206-13.
14. Burkhardt H, Wehling M. Probleme bei der Pharmakotherapie älterer Patienten. Internist. 2010;51 (6):737-46.
15. Rottlaender D, Scherner M, Schneider T, Erdmann E. Mulimedikation, Compliance und Zusatzmedikation bei Patienten mit kardiovaskulären Erkrankungen. Deutsche medizinische Wochenschrift. 2007;132 (4):139-44.
16. Sachverständigenrat zur Begutachtung der Entwicklung im Gesundheitswesen: Kooperation und Verantwortung. Voraussetzung einer zielorientierten Gesundheitsversorgung. 2007.
17. Stark RG, John J, Leidl R. Health care use and costs of adverse drug events emerging from outpatient treatment in Germany. A modelling approach. BMC Health Serv Res 11(1). 2011:9.
18. Burkhardt H, Wehling M, Gladisch R. Prävention unerwünschter Arzneimittelwirkungen bei älteren Patienten Z Gerontol Geriat. 2007;40 (4):241-54.

19. Field TS, Gurwitz JH, Harrold LR, Rothschild J, DeBellis KR, et al. Risk factors for adverse drug events among older adults in the ambulatory setting. Journal of the American Geriatrics Society. 2004;52 (8):1349-54.
20. Gurwitz JH, Field TS, Harrold LR, Rothschild J, DeBellis KR, Seger AC, et al. Incidence and preventability of adverse drug events among older persons in the ambulatory setting. JAMA. 2003;289 (9):1107-16.
21. Murray MD, Ritchey ME, Wu J, Tu W. Effect of a pharmacist on adverse drug events and medication errors in outpatients with cardiovascular disease. Arch Intern Med. 2009;169 (8):757-63.
22. Holt S, Schmiedl S, Thürmann PA. Potentially inappropriate medications in the elderly: The PRISCUS List. Deutsches Ärzteblatt international. 2010;107:543-51.
23. Barnett MJ, Frank J, Wehring H, Newland B, von Muenster S, Kumbera P, et al. Analysis of pharmacist-provided medication therapy management (MTM) services in community pharmacies over 7 years. Journal of Managed Care Pharmacy. 2009;15 (1):18-31.
24. Isetts BJ, Schondelmeyer SW, Artz MB, Lenarz LA, Heaton AH, Wadd WB, et al. Clinical and economic outcomes of medication therapy management services: the Minnesota experience. Journal of the American Pharmacists Association. 2008;48 (2):203-11.
25. Oliveira de DR, Brummel AR, Miller DB. Medication Therapy Management: 10 years of experience in a large integrated health care system. Journal of Managed Care Pharmacy. 2010;16 (3):185-95.
26. Smith SR, Catellier DJ, Conlisk EA, Upchurch GA. Effect on health outcomes of a community-based medication therapy management program for seniors with limited incomes. American Journal of Health-System Pharmacy. 2006;63:372-9.
27. Doucette WR, McDonough RP, Klepser D, McCarthy R. Comprehensive Medication Therapy Management: identifying and resolving drug-related issues in a community pharmacy. Clinical Therapeutics. 2005;27 (7):1104-11.
28. Christensen DB, Roth M, Trygstad T, Byrd J. Evaluation of a pilot medication therapy management project within the North Carolina state health plan. Journal of the American Pharmacists Association. 2007;47 (4):471-83.
29. Weingarten SR, Henning JM, Badamgarav E, Knight K, Hasselblad V, Gano A, Jr., et al. Interventions used in disease management programmes for patients with chronic illness-which ones work? Meta-analysis of published reports. BMJ. 2002 Oct 26;325(7370):925.
30. Wensing M, Wollersheim H, Grol R. Organizational interventions to implement improvements in patient care: a structured review of reviews. Implement Sci. 2006;1:2.
31. Reisig M. Implementierung und Evaluation eines computergestützten Systems zur Optimierung der individuellen Arzneimitteltherapie: Universität Erlangen; 2006.
32. Krüger M, Griese N. Home Medication Review - a structured and collaborative service for multimorbid type 2 diabetic patients provided by community pharmacies and practitioners In: GAA, editor. 17 Jahrestagung der GAA; Düsseldorf2010.
33. Kruse J, Waltering I, Puteanus U, Hempel G. Potentials to optimize the pharmacotherapy of geriatric residents in nursing homes by pharmaceutical care. In: GAA, editor. 16 Jahrestagung der GAA; Berlin2009.
34. Abschlussbericht 2004-2007 zum Modellprojekt "Evaluation der Effektivität und Effizienz eines integrierten Versorgungssystems für ältere pflege- und hilfebedürftige Menschen am Beispiel der Pflege- und Wohnberatung in Ahlen". 2008.
35. Patterson SM, Hughes C, Kerse N, Cardwell CR, Bradley MC. Interventions to improve the appropriateness of polypharmacy for older people. Cochrane Database of Systematic Review 2012; 5: pub 2.
36. Samsa GP, Hanlon JT, Schmader KE et al. A summated score for the medication appropriateness index: development and assessment of clinimetric properties including content validity. J Clin Epidemiology 1994; 47(8): 891-896.

37. Spinewine A, Swine C, Dhillon S et al. Effect of a collaborative approach on the quality of prescribing for geriatric inpatients: a randomized, controlled trial. Am Geriatr Soc. 2007; 55(5): 658-665.
38. Woertman W, de Hoop E, Moerbeek M et al. Stepped wedge designs could reduce the required sample size in cluster randomized trials. J Clin Epidemiology 2013; Mar 22: Epub ahead of print.
39. Hussey MA, Hughes,JP. Design and analysis of stepped wedge cluster randomized trials. Contemp clin trials 2007; 28(2): 182-191.

9 ANHANG

9.1 Liste der 46 chronischen Erkrankungen (nach van Bussche et al. 2011 [5])

No.	Chronic condition	ICD-10-codes
1	Hypertension	I10-I15
2	Hyperlipidemia	E78
3	Chronic low back pain	M40-M45, M47, M48.0-M48.2, M48.5-M48.9, M50-M54
4	Severe vision reduction	H17-H18, H25-H28, H31, H33, H34.1-H34.2, H34.8-H34.9, H35-H36, H40, H43, H47, H54
5	Osteoarthrosis	M15-M19
6	Diabetes mellitus	E10-E14
7	Chronic ischemic heart disease	I20, I21, I25
8	Thyroid dysfunction	E01-E05, E06.1-E06.3, E06.5, E06.9, E07
9	Cardiac arrhythmias	I44-I45, I46.0, I46.9, I47-I48, I49.1-I49.9
10	Obesity	E66
11	Hyperuricemia/Gout	E79, M10
12	Prostatic hyperplasia	N40
13	Lower limb varicosis	I83, I87.2
14	Liver disease	K70, K71.3-K71.5, K71.7, K72.1, K72.7, K72.9, K73-K74, K76
15	Depression	F32-F33
16	Asthma/COPD	J40-J45, J47
17	Noninflammatory gynecological problems	N81, N84-N90, N93, N95
18	Atherosclerosis/PAOD	I65-I66, I67.2, I70, I73.9
19	Osteoporosis	M80-M82
20	Renal insufficiency	N18-N19
21	Cerebral ischemia/Chronic stroke	I60-I64, I69, G45
22	Cardiac insufficiency	I50
23	Severe hearing loss	H90, H91.0, H91.1, H91.3, H91.8, H91.9
24	Chronic cholecystitis/Gallstones	K80, K81.1
25	Somatoform disorders	F45
26	Hemorrhoids	I84
27	Intestinal diverticulosis	K57
28	Rheumatoid arthritis/Chronic polyarthritis	M05-M06, M79.0
29	Cardiac valve disorders	I34-I37
30	Neuropathies	G50-G64
31	Dizziness	H81-H82, R42
32	Dementia	F00-F03, F05.1, G30, G31, R54
33	Urinary incontinence	N39.3-N39.4, R32
34	Urinary tract calculi	N20
35	Anemia	D50-D53, D55-D58, D59.0-D59.2, D59.4-D59.9, D60.0, D60.8, D60.9, D61, D63-D64
36	Anxietiy	F40-F41
37	Psoriasis	L40
38	Migraine/chronic headache	G43, G44
39	Parkinson's disease	G20-G22

40	Cancers	C00-C14, C15-C26, C30-C39, C40-C41, C43-C44, C45-C49, C50, C51-C58, C60-C63, C64-C68, C69-C72, C73-C75, C81-C96, C76-C80, C97, D00-D09, D37-D48
41	Allergies	H01.1, J30, L23, L27.2, L56.4, K52.2, K90.0, T78.1, T78.4, T88.7
42	Chronic gastritis/GERD	K21, K25.4-K25.9, K26.4-K26.9, K27.4-K27.9, K28.4-K28.9, K29.2-K29.9
43	Sexual dysfunction	F52, N48.4
44	Insomnia	G47, F51
45	Tobacco abuse	F17
46	Hypotension	I95

ICD = International Classification of Diseases (10th edition)

9.2 Muster der Patienteninformation und -einverständniserklärung

9.3 Muster der Dokumentationsbögen

10 UNTERSCHRIFTEN
